# *In silico* optimization of regenerative cell therapy in the infarcted human ventricles to mitigate arrhythmic burden

**DOI:** 10.1016/j.stemcr.2026.103007

**Published:** 2026-07-09

**Authors:** Leto Luana Riebel, Zhinuo Jenny Wang, Xin Zhou, Lucas Arantes Berg, Cristian Trovato, Blanca Rodriguez

**Affiliations:** 1 Department of Computer Science, British Heart Foundation Centre of Research Excellence, University of Oxford, Oxford, UK; 2 Systems Medicine, Clinical Pharmacology & Safety Science, R&D, AstraZeneca, Cambridge, UK

**Keywords:** in silico trials, computer modelling and simulation, cell therapy, myocardial infarction, heart failure, arrhythmias

## Abstract

Myocardial infarction remains a frequent cause of heart failure and mortality. Cell therapy has shown promise in regenerating the damaged tissue, but delivered cells may beat spontaneously and produce ventricular arrhythmias, hindering clinical application. Here, we conducted multiscale computer simulations of the electrical activity of the infarcted human ventricles including the cardiac conduction system to identify and mitigate pro-arrhythmic mechanisms following cell delivery. Firstly, our simulations show how arrhythmic risk increases from before to after cell injection and further during the first two weeks post-delivery. Secondly, we suggest that concurrently targeting the funny current, the inward rectifier potassium current, the sodium-potassium pump current, and the rapid delayed outward rectifier potassium current may reduce automaticity and re-entry while maximizing calcium transient amplitude and thus contractility. Our study demonstrates how modeling and simulation enables the design of anti-arrhythmic strategies to improve therapy safety while preserving efficacy.

## Highlights

• Computer simulations assess cell therapy safety after infarction
• Automaticity-induced arrhythmias after virtual cell delivery are modulated by scar size
• Re-entry risk increases after virtual cell injection and is highest two weeks post delivery
• Computer modelling and simulation identifies ionic targets to minimise arrhythmic risk

## Graphical abstract

**Figure:**
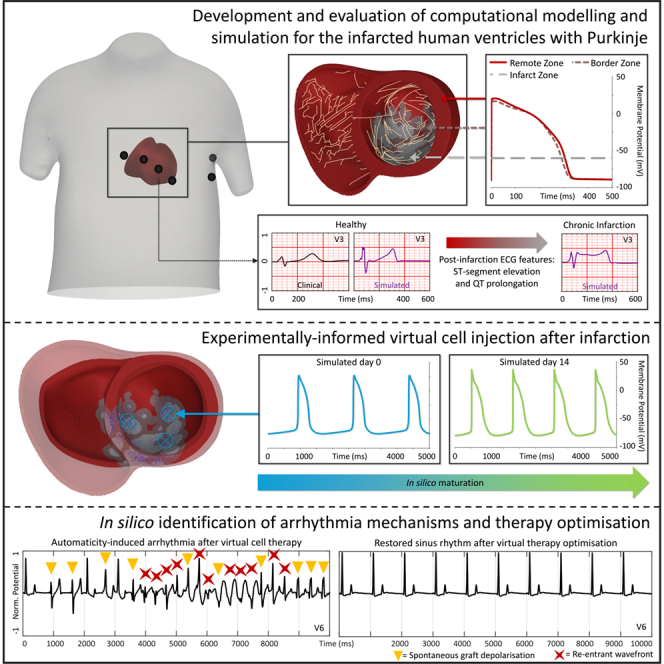

## Teaser

In their computer simulations of the infarcted human ventricles, Riebel and colleagues demonstrate that cell delivery may lead to arrhythmias through two mechanisms, automaticity and re-entry, which are modulated by scar size. The authors further design and evaluate anti-arrhythmic strategies following virtual cell delivery and identify combinations of ionic current modulation that improve therapy safety while maximizing efficacy.

## Introduction

Myocardial infarction (MI) and its long-term consequences, particularly heart failure, remain among the leading causes of death worldwide. Since adult human hearts do not sufficiently regenerate on their own, myocardial damage is largely irreversible. To ease the global burden of heart failure and improve cardiac function post-MI, delivery of stem cells has been explored (Jebran et al., 2025; Liu et al., 2018; Shiba et al., 2016). While pre-clinical studies have shown promising effects, ventricular arrhythmias, particularly during the first weeks after cell injection, have been observed and are suspected to arise from the immature cells’ spontaneous beating (Chong et al., 2014; Liu et al., 2018; Marchiano et al., 2023; Romagnuolo et al., 2019).

Previous studies have identified upregulation of the inward rectifier potassium current (I_K1_) and suppression of the funny current (I_f_) as crucial anti-automaticity targets (Goversen et al., 2018; Kim et al., 2015; Verkerk and Wilders, 2023). One recent study found a combination of these together with a block of the T-type calcium current and the sodium-calcium exchanger current (I_NaCa_) necessary to suppress spontaneous beating (Marchiano et al., 2023), highlighting a complex mechanism behind the cells’ automaticity. While these strategies offer a vital way to reduce arrhythmias, it is unclear how they might impact the cells’ calcium dynamics and, consequently, their efficacy. Particularly the sodium-calcium exchanger is crucial for regulating intracellular calcium levels throughout the action potential and may hence affect physiological calcium-induced calcium release and contractility (Henderson et al., 2004; Neco et al., 2010). In addition to automaticity-induced arrhythmias, previous human-based *in silico* studies have highlighted a variety of re-entry pathways caused by the cells’ immature tissue conductivity and action potential morphology (Fassina et al., 2023; Riebel et al., 2024; Yu et al., 2022); however, due to limited clinical trial data, these findings remain to be corroborated. To enable safe clinical application of cell therapy post-MI, effective anti-automaticity as well as anti-re-entry targets need to be identified.

The development and testing of new treatments, whether they reach the clinic or not, is expensive (Sertkaya et al., 2024). Multiscale human modeling and simulation offers a powerful technology to augment the drug discovery and development process by identifying safety risks early on (Passini et al., 2021; Viceconti et al., 2021). Furthermore, simulation studies present an efficient tool to design anti-arrhythmic strategies that improve therapy success (Dasí et al., 2024; Rosales et al., 2024).

In this study, we harness the powers of human-based modeling and simulation to identify and optimize ionic targets that reduce both automaticity-induced and re-entrant arrhythmias after cell delivery, as a post-MI therapy. For this, we conduct computer simulations from the action potential to the electrical excitation and relaxation of the ventricles to evaluate pro-arrhythmic mechanisms following cell delivery in the infarcted human ventricles including the Purkinje conduction system (Riebel et al., 2024). To computationally model electrical maturation of injected cells from day 0 (D0) to day 14 (D14), we consulted experimentally reported ion channel gene expression data of *in vivo* maturing injected cells from Marchiano et al. (2023). We then set out to determine: (1) the risk of automaticity-induced arrhythmias at D0 and D14 after cell injection, also considering variability in scar size, (2) the risk of re-entry before and at D0 and D14 after delivery, (3) novel ionic targets that supress spontaneous beats while maintaining physiological calcium transients, and (4) a combination of ionic targets to optimize therapy safety by reducing automaticity-induced as well as re-entrant arrhythmias.

## Results

### Establishing credibility: Computer simulations of automaticity-induced arrhythmias two weeks after cell delivery

As expected from experimental observations (Chong et al., 2014; Marchiano et al., 2023; Romagnuolo et al., 2019) and in line with our previous study (Riebel et al., 2024), human stem cell-derived cardiomyocytes (hPSC-CMs) immediately after delivery (D0) did not produce spontaneous depolarizations in the ventricles (see Figure S1A). To investigate variability in hPSC-CM electrophysiology and corresponding automaticity, we created a rapid hPSC-CM model by upregulating the sodium-calcium exchanger, the funny current, and the sarco-endoplasmic reticulum calcium ATPase (SERCA) in our baseline hPSC-CM model (see STAR Methods for further detail). While at D0 this rapid hPSC-CM model caused spontaneous beats at 46 beats per minute (bpm) in the ventricles, it was overridden by 1 Hz sinus pacing (see the lack of automaticity in Figure S1C).

**Figure 1:**
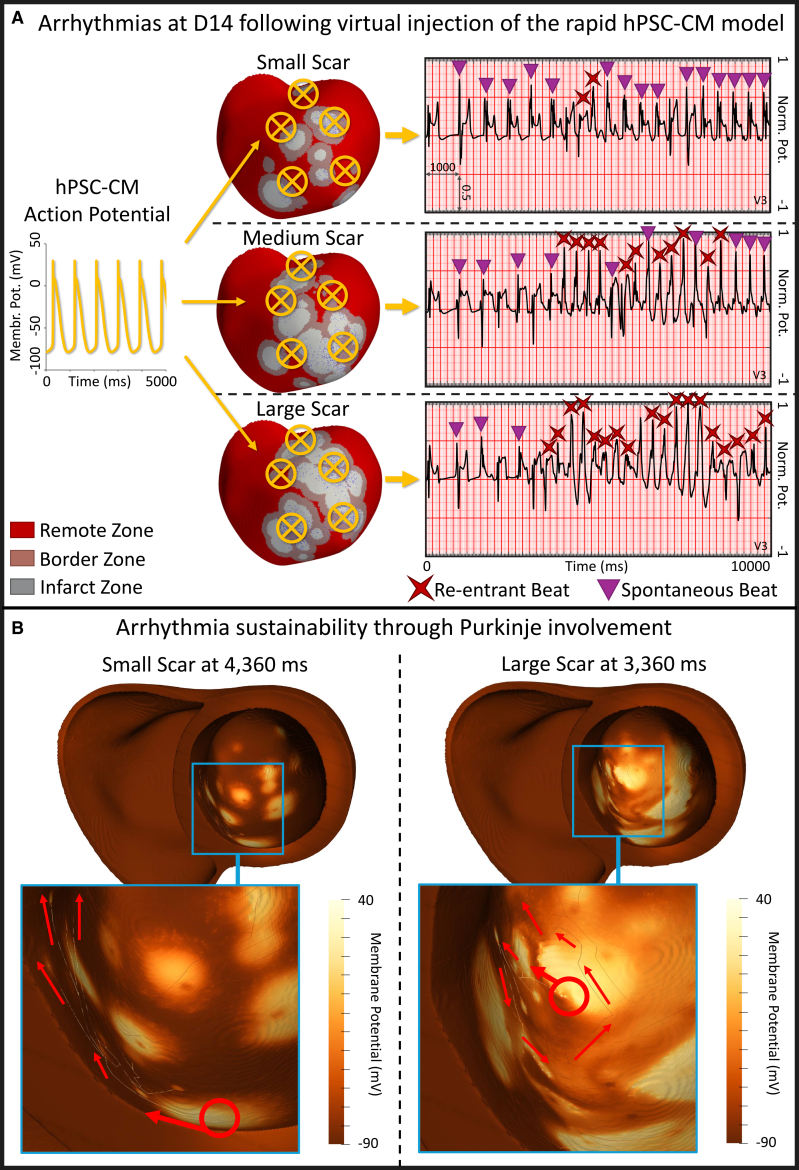
Automaticity-induced arrhythmia burden at D14 after virtual injection of rapidly beating hPSC-CMs in a human biventricular model with Purkinje and three different anteroseptal MI scars (A) Left: action potential traces of the rapid D14 hPSC-CMs beating spontaneously in virtual isolation. Middle: five out of six virtual hPSC-CM injection locations highlighted in the biventricular mesh (remaining one is located in the septum). Right: ECG lead V3 traces of arrhythmias after virtual cell injection during 1 Hz stimulation at the bundle of His. (B) Snapshots from Video S1 corresponding to the ECGs in A highlighting the involvement of the Purkinje system in sustaining the arrhythmias. The red circles identify the Purkinje-myocyte junctions through which the wavefront re-entered; red arrows indicate the subsequent path of the wavefront through the Purkinje conduction network. Also see Video S1 and Figure S1.

At D14 post-delivery, the baseline hPSC-CM model caused ectopy in the ventricles with 42 bpm but, again, was suppressed by sinus pacing (see Figure S1B). However, as shown in Figure 1A, the rapid hPSC-CM model, which beat spontaneously with a frequency of 66 bpm at D14 in isolation, produced spontaneous ventricular depolarizations in all three investigated scars. This spontaneous beating increased over time and facilitated ventricular tachycardia-like arrhythmias with intermittent re-entrant wavefronts in all three scars. As highlighted in the annotated ECGs of Figure 1A, the proportion of re-entrant wavefronts compared to spontaneous depolarizations decreased with decreasing scar size (from 82% in the large scar over 52% in the medium scar to 12% in the small scar). While re-entry dominated over spontaneous activity in the large scar, automaticity appeared to reach a steady-state toward the end of the 10 s simulations in the medium and small scar (see Video S1). Spontaneous depolarizations occurred up to every 420 ms in the ventricles corresponding to a tachycardia frequency of approximately 140 bpm, similar to pre-clinical observations (Marchiano et al., 2023). hPSC-CM automaticity increased in the ventricles compared to isolation (140 versus 66 bpm); as we showed previously (Riebel et al., 2024), hPSC-CM automaticity in the ventricles is favored by depolarized chronic infarcts and may further increase under fast heartrates, which here were facilitated through re-entrant rotors (see ECGs and membrane potentials in Figure 1).

Video S1. Automaticity-induced arrhythmias in the small (left) and large scar (right) at day 14 (D14) after virtual delivery of rapid stem cell-derived cardiomyocytes Note, automaticity reaches a steady state at the end of the 10 s simulation in the small scar with a frequency of approximately 140 beats per minute, while in the large scar, automaticity causes re-entry and polymorphic tachycardia. Related to main text Figure 1.

### Quantifying re-entry risk: Arrhythmic burden increases from before to after virtual cell delivery and from day 0 to day 14

**Figure 2:**
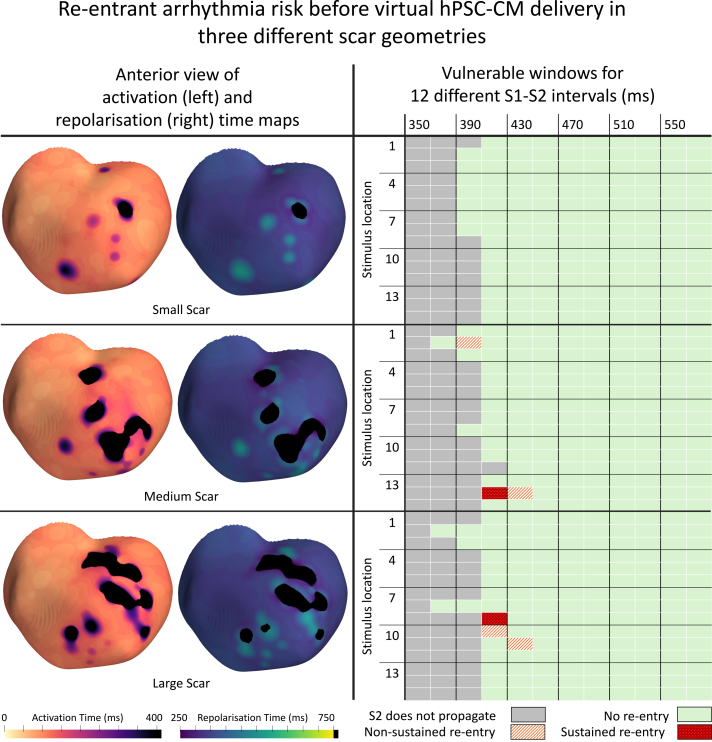
Re-entry risk in a human-based biventricular model with three scars before virtual delivery of baseline hPSC-CMs Left: activation and repolarization time maps. Right: vulnerable windows for different S1-S2 intervals and S2 stimulus locations. Note, ectopic stimulus locations differ in each scar and were ordered from most anterior-basal to most inferior-apical.

We next set out to quantify the re-entry risk after cell delivery by applying ectopic stimuli in the host border zone (S1-S2 stimulus protocol, further described in the STAR Methods). As shown in Figure 2, we observed few re-entries before virtual injection of the baseline hPSC-CM model. In the small scar, which contained spread out scar islands, no re-entries were observed. In the medium and large scar each, two non-sustained and one sustained re-entry occurred. All re-entries in the large scar and two out of three in the medium scar were induced by ectopic stimulus locations close to the apex and followed a regular pattern. As the scar cores were unexcitable and only slowly conducting, the wavefront traveled through isthmuses between the dense scar cores and re-entered at the site of the applied ectopic stimulus. The third re-entry in the medium scar was facilitated through a Purkinje-myocyte junction on the anterior left ventricular wall on the edge between infarct and border zone.

**Figure 3:**
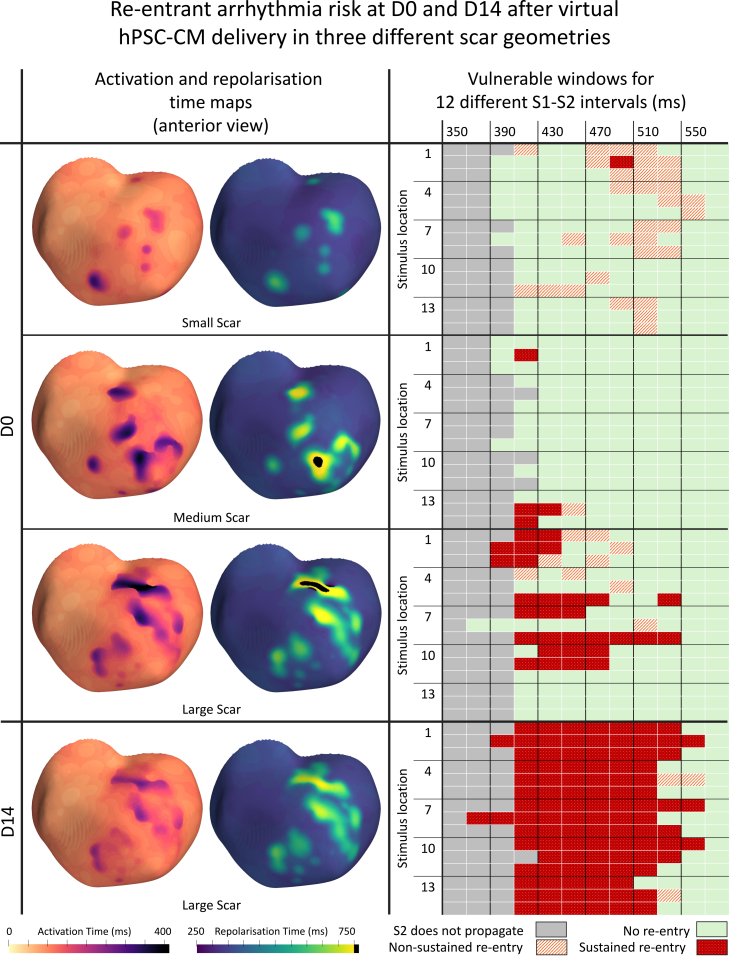
Re-entry risk in a human-based biventricular model with three different scars at D0 and D14 after virtual delivery of the baseline hPSC-CM model Left: activation and repolarization time maps. Right: vulnerable windows for different S1-S2 intervals and S2 stimulus locations. Note, ectopic stimulus locations differ in each scar and were ordered from most anterior-basal to most inferior-apical. Also see Videos S2 and S3.

As shown in Figure 3, the vulnerable windows (i.e., the range of time intervals between sinus rhythm and applied ectopic stimulus during which re-entry was inducible), increased from before to after virtual cell delivery in all three investigated scars: from 0 to 640 ms in the small scar, from 60 to 100 ms in the medium scar, and from 60 to 760 ms in the large scar (compare Figures 2 and 3). While re-entry risk rose in all three scars, it did not linearly increase with larger scar size—i.e., re-entry risk at D0 was highest in the large scar but lowest in the medium scar. Re-entry sustainability, however, was substantially lower in the small scar (3%, i.e., 1 out of 32 re-entries after cell delivery) than in the medium (33% before and 80% after delivery) and large scar (33% before and 76% after delivery). We observed that re-entries in the small scar relied on Purkinje involvement with two Purkinje-myocyte junctions isolated in the center of the scar cores emerging as particularly pro-arrhythmic (see Video S2). In the medium and large scar, the infarct cores were larger and instead, re-entries relied on a mix of Purkinje and tissue pathways. Thus, the large scar provided several routes for conduction block and re-entry, with no single pro-arrhythmic area emerging. In the medium scar, the tissue pathways for re-entry were more limited and 80% of re-entries involved the same large apical scar core (visible as not fully repolarizing in the repolarization map of Figure 3). As highlighted when comparing the activation time maps in Figures 2 and 3, hPSC-CMs improved conductivity and excitability of the infarct and allowed for wavefronts to travel through scar cores, which before cell delivery were inaccessible (see Video S3).

Video S2. The only sustained re-entry at day 0 (D0) after cell delivery in the small scar This was caused by stimulating 490 ms after the last sinus beat at S2 location 2 (basal septum). Note, that the re-entry is sustained through two Purkinje-myocyte junctions on the anterior wall. Related to main text Figure 3.

Video S3. Re-entry before (left) and at D0 after (right) cell delivery in the large chronic scar Induced by stimulating 430 ms after the last sinus beat from S2 location 11 (apical). Note, that the re-entry in the control case lasts only for a single cycle, while the one at D0 after cell delivery is sustained. Related to main text Figure 3.

Next, we investigated the re-entry risk at D14 post-delivery in the large scar (as this was the most pro-arrhythmic scenario at D0); the vulnerable window increased from 760 to 2,080 ms, as shown in the bottom of Figure 3. 97% (101 out of 104) of those D14 re-entries sustained for at least two re-entry cycles. As highlighted by the repolarization time maps in Figure 3, hPSC-CMs further improved conductivity within the infarct from D0 to D14. This was due to an increase of graft conduction velocity from 10 cm/s at D0 to 14.5 cm/s at D14—as conductivities were maintained, this was caused by maturation of ionic currents, particularly a 2.5-fold increase in the fast sodium current’s (I_NaF_) conductance (see STAR Methods section). The improved excitability within the infarct enabled new paths for the re-entrant wavefront; slow transmural conduction pathways emerged as particularly pro-arrhythmic at D14.

In summary, while virtually delivered cells improved conduction through the infarct and reduced activation time gradients across the ventricles, they also enabled new re-entry pathways transmurally and across the scar, particularly at D14 after delivery. This pro-arrhythmic substrate was due to the virtually injected cells having a longer action potential duration than the host cardiomyocytes, thus promoting heterogeneous ventricular repolarization, refractoriness, and pro-arrhythmic conduction block.

### Virtual target discovery: Regulating the sodium-potassium pump to suppress automaticity-induced arrhythmias

**Figure 4:**
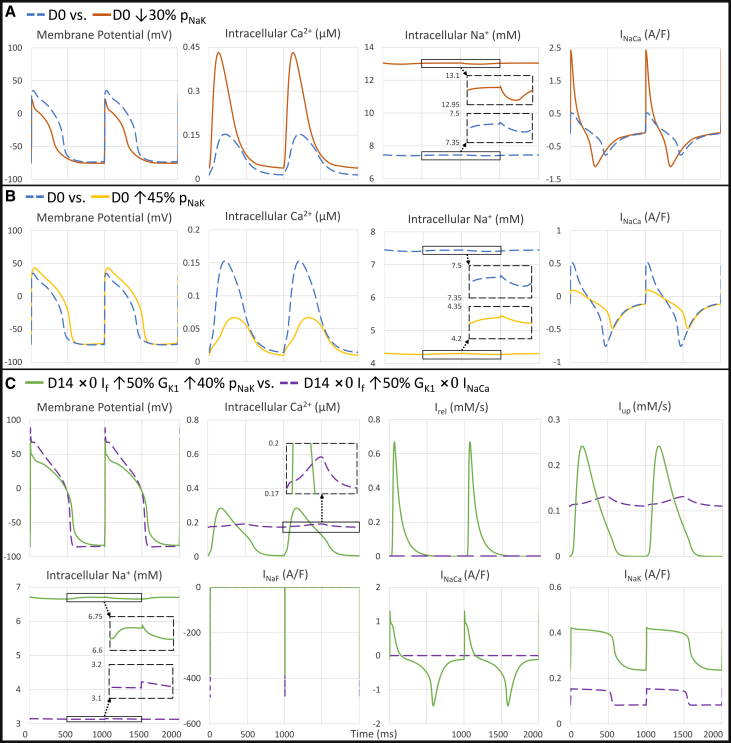
Action potential and ionic current traces of different hPSC-CM models virtually beating in isolation under 1 Hz pacing; beat 1,000 shown (A) hPSC-CMs at D0 with and without downregulating the maximum sodium-potassium pump current (p_NaK_). (B) hPSC-CMs at D0 with and without upregulating p_NaK_. (C) hPSC-CMs at D14 with full funny current (I_f_) block, upregulation of potassium currents’ conductances (G_K1_ and G_Kr_) and either full block of the sodium-calcium exchanger or p_NaK_ upregulation. Also shown are intracellular calcium (Ca^2+^) and sodium (Na^+^), the ryanodine receptor-sensitive release current (I_rel_), the sarco-endoplasmic reticulum calcium ATPase (SERCA) current (I_up_), and the fast sodium current (I_NaF_). Also see Figures S3–S5.

To identify ionic targets that suppress hPSC-CM automaticity, we carried out a cellular sensitivity analysis by scaling ion channel conductances and recording their effects on the cells’ spontaneous beating rate in virtual isolation. Our results, shown in Figure S2, identified the spontaneous beating frequency to be most sensitive to the sodium-potassium pump, with both a 45% increase as well as a 30% decrease in its maximum value (model parameter p_NaK_) abolishing automaticity at D0. As shown in Figure 4A, reducing the maximum pump current caused intracellular sodium accumulation leading to an increase in the reverse mode of the sodium-calcium exchanger (I_NaCa_, first spike in Figure 4A, right panel) and, ultimately, intracellular calcium accumulation and action potential duration shortening, as discussed in other studies (Bueno-Orovio et al., 2014; Faber and Rudy, 2000). Counterintuitively, and as depicted in Figure S3A, intracellular sodium accumulation over time resulted in an ultimately increased pump current, even though its initial maximum value was reduced. Further investigation in our model also showed an increase in the sarcoplasmic reticulum’s calcium concentration. In the absence of applied pacing, this resulted in an impaired recovery of the ryanodine receptor-sensitive release current’s (I_rel_) inactivation gate (model parameter RyRc) leading to a reduced calcium-induced calcium release through the ryanodine receptors and a lack of spontaneous activity. While 1 Hz pacing caused physiological action potentials at D0 (see Figure S3A), calcium overload occurred in the sarcoplasmic reticulum at D14, where only diminished calcium transients and short action potentials could be produced (see Figure S4A). As such action potential heterogeneity may be pro-arrhythmic, we discarded lowering the maximum sodium-potassium pump current and considered its upregulation further.

Upregulation of the maximum sodium-potassium pump current suppressed spontaneous beats through the opposite effect, i.e., intracellular sodium and the reverse mode of the sodium-calcium exchanger were reduced (I_NaCa_, first spike in Figure 4B, right panel) therefore leading to lower intracellular and sarcoplasmic reticulum calcium concentrations impairing the calcium clock (see Figures 4B and S3B). While increasing the maximum pump current produced physiological (but longer) action potentials in virtual isolation, the calcium transient amplitude was reduced by approximately 55% (see Figure S3B). At D14, increased maximum pump current on its own was not sufficient to abolish automaticity; hence, we combined it with a full block of the funny current and upregulation of the inward rectifier potassium current, as suggested also in other studies (Goversen et al., 2018; Marchiano et al., 2023). For a 50% increase in the conductance of the inward rectifier potassium current, a 40% and 65% increase in maximum sodium-potassium pump current was enough to stop spontaneous beating in the baseline and rapid hPSC-CM model, respectively. Pacing produced physiological action potentials and calcium transients in virtual isolation at D0 and D14, as shown in Figures S5 and S4B, respectively.

We further compared our suggested upregulation of maximum sodium-potassium pump current with the state-of-the-art approach of knocking out the sodium-calcium exchanger to inhibit stem cell automaticity (Marchiano et al., 2023). As depicted in Figure 4C, this comparison showed that (together with an increased inward rectifier potassium current and blocked funny current) fully blocking the exchanger caused intracellular calcium accumulation and small calcium transient amplitudes. We identified the main mechanism behind this as a substantially reduced ryanodine receptor-sensitive calcium release current, similar to when we reduced the maximum sodium-potassium pump current. Knock-out of the sodium-calcium exchanger, which is the main mechanism for calcium extrusion, caused accumulation of intracellular but also sarcoplasmic reticulum calcium. This led to insufficient relaxation of the ryanodine receptors’ activation and inactivation gates (RyRo and RyRc model parameters). In comparison, even though the calcium transient amplitude was reduced compared to the baseline hPSC-CM model with no ionic current modifications, upregulating the maximum sodium-potassium pump current produced more physiological calcium transients at both D0 and D14 (see Figures S5 and 4C, respectively).

### Virtual target optimization: Regulating multiple potassium currents to reduce automaticity-induced as well as re-entrant arrhythmias

**Figure 5:**
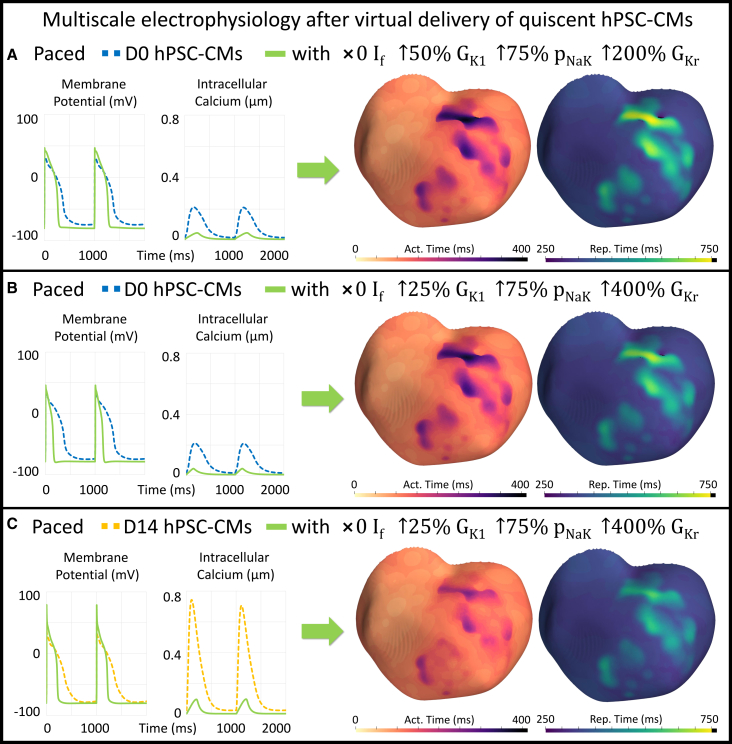
Left: action potentials and calcium transients of the baseline hPSC-CM model before and after optimisation in virtual isolation under 1 Hz pacing. Right: activation and repolarisation time maps after virtual delivery of optimized hPSC-CMs (green traces from the left) into a human-based biventricular chronic post-MI model Compare to activation and repolarization time maps before optimization in Figure 3. (A and B) Optimised hPSC-CM model at D0. (C) Optimised hPSC-CM model at D14. Note, the optimized hPSC-CMs (green traces on the left) did not beat spontaneously. I_f_: funny current; G_K1_: conductance of the inward rectifier potassium current; p_NaK_: maximum sodium-potassium pump current; G_Kr_: conductance of the rapid delayed outward rectifier potassium current.

**Figure 6:**
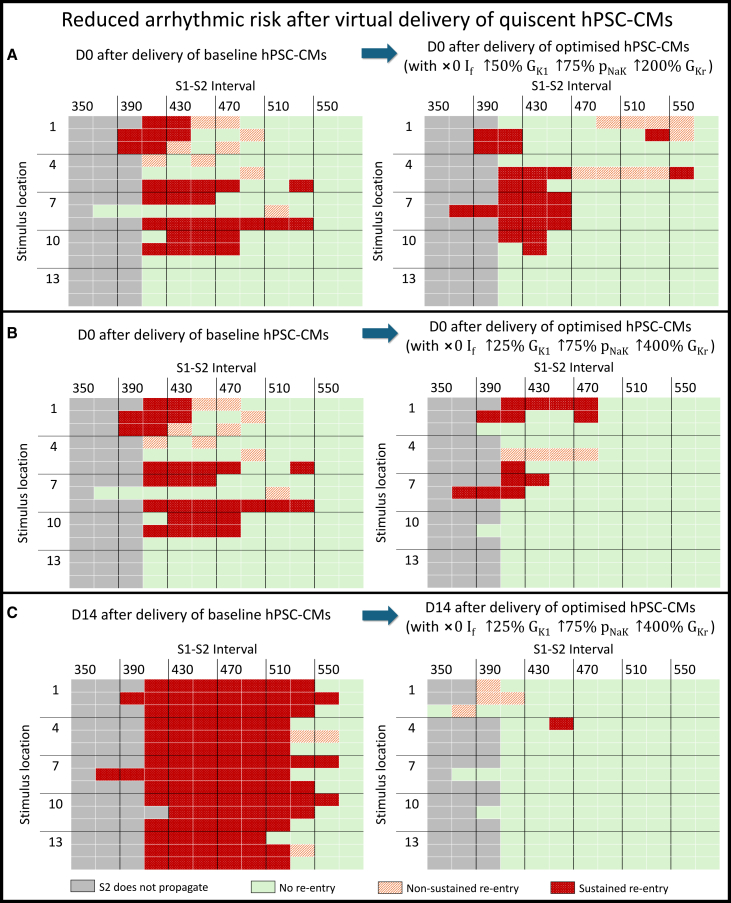
Comparison of re-entry risk after virtual delivery of baseline versus optimized hPSC-CMs into a human-based biventricular model with a large chronic infarct (A and B) Vulnerable windows for different S1-S2 intervals and S2 stimulus locations at D0 after virtual cell delivery. (C) Vulnerable windows for different S1-S2 intervals and S2 stimulus locations at D14 after virtual cell delivery. The ectopic stimulus for location 3 and S1-S2 interval of 330 ms (not shown in the right table) did not propagate. Note, ectopic stimulus locations were ordered from most anterior-basal to most inferior-apical. Also see Video S4. I_f_: funny current; G_K1_: conductance of the inward rectifier potassium channel; p_NaK_: maximum sodium-potassium pump current; G_Kr_: conductance of the rapid delayed outward rectifier potassium current.

Next, we investigated how to combine the identified anti-automaticity targets, analyzed above, with anti-re-entry targets. Our cellular hPSC-CM sensitivity analysis, shown in Figure S2, as well as previous studies (Fassina et al., 2023; Riebel et al., 2021), highlighted the rapid delayed outward rectifier potassium current (I_Kr_) as a promising target to modulate the longer hPSC-CM action potential toward adult cardiomyocyte values. Hence, we created an automated algorithm aiming to determine scaling factors for three potassium currents (inward rectifier potassium, rapid delayed outward rectifier potassium, and sodium-potassium pump current), in addition to full funny current block. As described in the STAR Methods, this algorithm aimed to optimize calcium transient amplitude as well as action potential duration by maximizing an optimization index that considers both. First, we used a target hPSC-CM action potential duration at 90% repolarization of 330 ms (approximately ¾ of the action potential at D0). Achieving the closest match to this target (while optimizing calcium transient amplitude), our algorithm suggested scaling the maximum sodium-potassium pump current and the conductances of the inward and rapid delayed outward rectifier potassium currents by 1.75, 1.5, and 3.0, respectively (see Figure S6). At D0, this ionic current scaling combination decreased hPSC-CM action potential duration at 90% repolarization to 284 ms in virtual isolation (see Figure 5A) and increased graft conduction velocity in tissue from 10 cm/s to 12.5 cm/s. Higher conduction improved activation of the scar (see repolarization time maps in Figures 3 and 5A) and, similarly to the unoptimized hPSC-CM model at D14, enabled new re-entry pathways. Accordingly, in our biventricular simulations, this set of ionic scalings reduced the vulnerable window only marginally from 760 to 680 ms at D0 (see Figure 6A).

As ventricular repolarization was not sufficiently homogenized, we reduced the target hPSC-CM action potential duration in our algorithm by approximately half (see Figure S7). As shown in Figures 6B and 6C, after scaling the maximum sodium-potassium pump current and potassium currents’ conductances (both inward and rapid delayed outward rectifier) by 1.75, 1.25, and 5 (respectively, and in addition to full funny current block), vulnerable windows decreased from 760 to 340 ms at D0 and from 2,080 to 100 ms at D14. Decreased arrhythmogenicity was the result of an increased graft conduction and reduced hPSC-CM action potential duration, which homogenized both activation and repolarization gradients, respectively (see Figures 5B and 5C and Video S4). Optimization increased graft conduction velocity by 25% and 24% at D0 and D14, respectively. Since conductivities were kept constant, this was caused by regulating these four potassium currents, all of which we previously showed to individually improve action potential upstroke velocity in the computational hPSC-CMs (Riebel et al., 2021). As shown in the calcium transient traces in Figures 5B and 5C, while our algorithm aimed to maximize the calcium transient amplitude, it was still substantially lower than before optimization.

Video S4. Comparing a re-entry before (left) and after optimization (right) of hPSC-CMs at D14 in the large scar Re-entry was induced by stimulating 410 ms after the last sinus beat at S2 location 2 (anteroseptal toward the base). Note, that the re-entry after delivery of the non-optimized hPSC-CMs is sustained, while after optimization, it lasts only for a single cycle. Related to main text Figure 6.

## Discussion

In this study we present a novel *in silico* multiscale human electrophysiological framework to optimize new treatments, such as regenerative cell therapy, through biophysically detailed computer simulations. First, in a human biventricular model with three different post-MI scars, validated against experimental and clinical data, we showed that while cells remained quiescent immediately after delivery, more rapidly beating cells produced automaticity two weeks later, causing monomorphic ectopy-driven ventricular tachycardia in the small and medium scar, and polymorphic re-entry driven tachycardia in the large scar. Next, in the absence of spontaneous beats, we quantified the re-entry risk, which increased from before to after cell delivery and further from day 0 to day 14. Finally, we demonstrated how our framework enables identifying and optimizing anti-arrhythmic ionic target combinations—blocking the funny current and upscaling the maximum sodium-potassium pump current as well as the inward and rapid delayed outward rectifier potassium currents emerged as a novel strategy to suppress spontaneous beating and reduce re-entrant arrhythmia susceptibility while maximizing calcium transient amplitude.

Our *in silico* framework of MI, including also the Purkinje conduction network, was constructed, calibrated, and evaluated using human-based experimental and clinical evidence and incorporates extensive biophysical knowledge gained throughout the development of modeling and simulation technologies (Camps et al., 2024; Mincholé et al., 2019; Tomek et al., 2019; Zhou et al., 2024). As described in the STAR Methods section, credibility of our human-based simulation framework is provided by addressing verification, validation, and uncertainty quantification principles (Musuamba et al., 2021; Viceconti et al., 2021) in the present and previous studies, specifically in Riebel et al. (2024).

In our simulations, fast-beating virtual hPSC-CMs produced spontaneous depolarizations in the ventricles two weeks after delivery and facilitated ventricular tachycardia-like arrhythmias with a frequency of up to 140 bpm, as observed experimentally (Marchiano et al., 2023). While, hPSC-CM automaticity has been identified as the main driver of ventricular tachycardia in non-human primate studies (Liu et al., 2018), it has been noted that arrhythmias after cell delivery are rarely observed in small animal models and that heartrate and size may be a crucial factor (Chong et al., 2014). In our study, hPSC-CM automaticity initiated ventricular tachycardia but wave breakup and re-entry rotors were involved in sustaining it. In our small scar, automaticity prevailed as the dominant arrhythmia mechanism, highlighted by largely monomorphic tachycardia patterns in the ECG. With increasing scar size, wave breakup into re-entry rotors increased; in our large scar, the ECG was more chaotic, highlighting the absence of a single ectopic tachycardia source. Overall, our human-based model with clinically reported infarct masses suggests that while automaticity may indeed produce regular ventricular tachycardia-like patterns in small scars, spontaneous depolarizations may break into re-entrant rotors in large scars and facilitate more chaotic arrhythmia patterns—which may not surface in animal models with smaller scars. Delivery modality may also affect arrhythmic risk; recent experiments of cell sheet transplantation, rather than cell injection, showed no arrhythmias in non-human primates (Jebran et al., 2025). Additionally, the Purkinje conduction system has been shown crucial in ventricular tachycardia sustainability in human (Bayer et al., 2025; Haissaguerre et al., 2016) and also played a critical role in arrhythmia sustainability in our study.

Maturation of delivered cells was a crucial factor for arrhythmic risk in our study (which showed no spontaneous depolarizations at day 0 after virtual delivery) and has also been highlighted experimentally (Marchiano et al., 2023). In another *in silico* study, Paci et al. (2012) used experimental evidence to construct early and late maturation cells. Similar to our study, their matured model had increased inward rectifier and transient outward potassium, fast sodium, sodium-calcium exchanger, and L-type calcium currents but, contrarily, decreased funny and rapid delayed outward rectifier potassium currents (Paci et al., 2012). According to Marchiano et al. (2023), while the genes encoding the funny and rapid delayed outward rectifier potassium currents increase initially, possibly contributing to the surge of arrhythmias two weeks after injection, they ultimately decrease by day 84. It should be noted, that Marchiano et al. (2023) investigated gene expression of hPSC-CMs that had matured for 18–20 days *in vitro* before delivery. Furthermore, to estimate ion channel conductances in our hPSC-CM models from experimental hPSC-CM gene expression data (Marchiano et al., 2023), we consulted experimental insights relating gene expression to functional change in human cell line studies (Stevens and Brown, 2013). However, as other factors may impact the final ion channel function, future work may create D0 and D14 hPSC-CM populations or consult multi-omics data to assess this uncertainty. While we scaled the sarco-endoplasmic reticulum calcium ATPase, sodium-calcium exchanger, and funny currents by 2.5 to create rapidly beating D0 and D14 hPSC-CM models, Gibbs and Boyle (2026) simulated fast hPSC-CM automaticity by increasing the funny, rapid and slow delayed outward rectifier potassium, and T-type calcium current conductances 11.5-fold and by blocking the inward rectifier potassium current. Their fast hPSC-CM model beat every 250 ms and produced automaticity-induced tachycardia-like arrhythmias in human ventricular slice models (Gibbs and Boyle, 2026).

Electrophysiological maturation, particularly of the fast sodium current, also caused an increase in our hPSC-CM model’s conduction velocity from 10 cm/s at D0 to 14.5 cm/s at D14. It should be noted that while experimental studies record similar D14 conduction velocities, they may show substantially slower conduction at earlier time points; however, they often investigate cell fibers without transplanting them (Hansen et al., 2018). In addition to electrophysiological maturation, maturing connectivity between the graft and host, as suggested experimentally (Dhahri et al., 2022), may further increase conduction. *In silico* studies have suggested a link between this evolving graft-to-host coupling and arrhythmic risk (Fassina et al., 2023; Gibbs et al., 2023; Rosales et al., 2024; Yu et al., 2022).

To suggest anti-arrhythmic strategies, we utilized the mechanistic nature of modeling and simulation with the sodium-potassium pump emerging as a novel target. Interestingly, experimental studies have suggested that the sodium-potassium pump current may increase as hPSC-CMs mature (Otsu et al., 2005), which could contribute to the reduction of automaticity after three weeks post injection and support our findings. The effects of regulating the sodium-potassium pump on spontaneous beating, although to a lesser extent, have also been highlighted in a sensitivity analysis by Paci et al. (2012). While our results indicate that for some hPSC-CM phenotypes targeting the sodium-potassium pump may be sufficient, modulating also the inward rectifier potassium and funny currents may be required in others. The latter two currents have long been known to impact the immaturity and automaticity of hPSC-CMs (Goversen et al., 2018; Marchiano et al., 2023; Verkerk and Wilders, 2023). While in our D0 (but not D14) model, funny current block and sufficient inward rectifier potassium current upregulation stopped automaticity, this may be insufficient in some cell types due to the calcium clock acting as a second depolarizing mechanism (Kim et al., 2015; Marchiano et al., 2023).

Next, we systematically identified ionic current combinations that minimize arrhythmic risk while allowing for physiological calcium transients. Current state-of-the-art agrees to block the funny current and upregulate the inward rectifier potassium current but, instead of targeting the sodium-potassium pump, further suggests knockout of the T-type calcium channel and the sodium-calcium exchanger (Marchiano et al., 2023). In our simulations, a full sodium-calcium exchanger block resulted in calcium accumulation and a reduced calcium transient amplitude, which, although to a lesser extent, has been suggested also in the supplemental material of Marchiano et al. (2023). However, as it is a main source of calcium extrusion (Ottolia et al., 2013), suppressing the sodium-calcium exchanger is generally associated with an increase in the calcium transient amplitude despite a decreased L-type calcium current (Ismaili et al., 2022; Ozdemir et al., 2008). Nevertheless, full sodium-calcium exchanger knock-out can facilitate intracellular and sarcoplasmic reticulum calcium accumulation and may ultimately hinder effective contractility (Henderson et al., 2004; Koivumäki et al., 2018; Figure 3; Koushik et al., 2001). Ultimately, the sodium-calcium exchanger and sodium-potassium pump depend on each other’s regulation of sodium concentrations—modulating either will affect the other. Interestingly, amiodarone has been suggested as an anti-arrhythmic strategy in pre-clinical trials of cell delivery (Nakamura et al., 2021) and is known to impact both, the sodium-calcium exchanger and the sodium-potassium pump (Gray et al., 1998; Watanabe and Kimura, 2000).

Finally, our simulations showed that ventricular arrhythmias may also be induced in the absence of spontaneous hPSC-CM depolarizations. Due to increasing conduction and action potential heterogeneity, re-entry risk increased from D0 to D14 after virtual cell delivery. To reduce this re-entry burden, in our previous suggested quiescent hPSC-CMs (blocking the funny current and upregulating inward rectifier potassium and maximum sodium-potassium pump currents), we further suggest an increase of the rapid delayed outward rectifier potassium current. Targeting this current to make hPSC-CM action potentials more adult-like has been put forward also in other *in silico* studies, which further identified the fast sodium channel as a potential target (Fassina et al., 2022, 2023). It should be noted that while we aimed to maximize the calcium transient amplitude, some reduction appeared necessary to avoid automaticity-induced arrythmias, linked to the cells’ calcium clock (Kim et al., 2015). Hence, our virtual hPSC-CMs showed a reduced calcium transient after optimization. This was largely caused by a block in the funny current, which on its own reduced the calcium transient amplitude by approximately 40%. Furthermore, blocking the inward rectifier potassium channel has been linked to larger calcium transients and active tension (Telle et al., 2025)—hence, increasing this current may further reduce contractility. While our suggested optimized ionic target combination includes such an increase in the inward rectifier potassium current, our results show that spontaneous beating may also be inhibited by funny current block and sufficient rapid delayed outward rectifier potassium and maximum sodium-potassium pump current upregulation alone, but will likely be affected by the underlying hPSC-CM type.

Overall, our study proposes targeting several potassium currents (including the funny, inward rectifier potassium, rapid delayed outward rectifier potassium, and sodium-potassium pump currents) to produce quiescent hPSC-CMs, which are also less likely to facilitate re-entries. These ion channels may be regulated through gene editing before cell delivery, which has shown successful in modulating several ionic currents concurrently in hPSC-CMs (Marchiano et al., 2023). Ultimately, optimal scaling factors will vary between cell types, maturation protocols, and the patient, e.g., patients with a slower repolarization may require the hPSC-CMs’ action potential to be shortened less. The computational pipeline we have presented here allows for fast and efficient optimization and testing of new treatments for a range of different cell lines, delivery modalities, and patient substrates.

## Resource availability

### Lead contact

Requests for further information and resources should be directed to the lead contact, Leto Luana Riebel (leto.riebel@cs.ox.ac.uk).

### Materials availability

This study did not generate new unique reagents.

### Data and code availability

Customized code for the 3D model solver is available at https://github.com/LLRiebel/MonoAlg3D_C-2023. The Purkinje network, biventricular meshes before and after virtual cell delivery, and ECG electrode locations are as in our previous study Riebel et al. (2024) and can be found in https://doi.org/10.5281/zenodo.14699735. Requests for any additional information and resources should be directed to the lead contact, Leto Luana Riebel (leto.riebel@cs.ox.ac.uk).

## Acknowledgments

This project was funded by: a 10.13039/501100000268BBSRC PhD iCASE (BB/V509395/1) and Russell Studentship Agreement with 10.13039/100004325AstraZeneca (R67719/CN001) for L.L.R. awarded to B.R., an Oxford-Bristol Myers Squibb Fellowship to X.Z. (R39207/CN063), a grant from the 10.13039/501100003593National Council for Scientific and Technological Development (10.13039/501100003593CNPq) to L.A.B. (446127/2024-8), a 10.13039/100010269Wellcome Trust Senior Research Fellowship in Basic Biomedical Sciences to B.R. (214290/Z/18/Z), an 10.13039/501100000266EPSRC Impact Acceleration Account Technology Fund (0016113), the Oxford 10.13039/501100000274BHF Center of Research Excellence (RE/24/130024), and the 10.13039/501100000266EPSRC project CompBioMed X (EP/X019446/1).

The Paci2020 model was provided by Dr. Michelangelo Paci and the Computational Biophysics and Imaging Group (CBIG) at Tampere University Foundation. An award for computer time was provided by the U.S. Department of Energy’s (DOE) Innovative and Novel Computational Impact on Theory and Experiment (INCITE) Program; this research used supporting resources at the 10.13039/100006224Argonne Leadership Computing Facilities at 10.13039/100006224Argonne National Laboratory, which is supported by the 10.13039/100006132Office of Science of the U.S. DOE under contract no. DE-AC02-06CH11357. Simulations were further enabled by a grant from the 10.13039/501100021847Swiss National Supercomputing Centre (10.13039/501100021847CSCS) under project ID lp107 on Alps.

We would like to thank Dr. Max Cumberland and Dr. Albert Dasi for feedback and discussions.

Parts of this study were included in L.L.R’s DPhil thesis at the University of Oxford, UK, titled Human-Based Multiscale Modeling and Simulation to Investigate Arrhythmias and Treatments in Ischemic Heart Disease (2024).

For the purpose of open access, the author has applied a Creative Commons Attribution (CC BY) public copyright license to any Author Accepted Manuscript version arising from this submission.

## Author contributions

Conceptualization, methodology, investigation, writing – original draft, L.L.R.; conceptualization, methodology, writing – review and editing, Z.J.W.; methodology, writing – review and editing, X.Z.; software, writing – review and editing, L.A.B.; conceptualization, writing – review and editing, supervision, C.T. and B.R.

## Declaration of interests

C.T. is an employee of AstraZeneca and may hold shares and/or stock options in the company.

## STAR★Methods

### Key resources table

**Table:** 

REAGENT or RESOURCE	SOURCE	IDENTIFIER
**Deposited data**		

Healthy biventricular human mesh with Purkinje tree and fiber directions	Camps et al. (2024)	https://doi.org/10.5281/zenodo.14046617
Human biventricular meshes with synthetic scars	Riebel et al. (2024)	https://doi.org/10.5281/zenodo.14699735
Human biventricular meshes after virtual cell injection	Riebel et al. (2024)	https://doi.org/10.5281/zenodo.14699735 (using the mesh with homogeneous ventricular-like virtual cell delivery)

**Software and algorithms**		

MATLAB	MathWorks	Version R2022b
ToR-ORd model of human ventricular cardiomyocytes	Tomek et al. (2019)	https://github.com/jtmff/torord (version model_Torord_dynCl_updatedPhiCaL.m)
Post-MI ToR-ORd model of human ventricular cardiomyocytes	Zhou et al. (2024)	https://github.com/xinzhoujoy/HumanPostMIVentricularCellElectromechanics
Trovato model of human Purkinje cells	Trovato et al. (2020)	https://www.cs.ox.ac.uk/insilicocardiotox/purkinje-models and https://github.com/rsachetto/MonoAlg3D_C/tree/master/src/models_library/trovato
Paci2020 model of stem cell-derived cardiomyocytes	Paci et al. (2020)	www.mcbeng.it/en/category/software.html
MonoAlg3D simulation software	Berg et al., 2026; Sachetto Oliveira et al. 2018	https://github.com/rsachetto/MonoAlg3D_C
Custom MonoAlg3D functions	This study and Riebel et al. (2024)	https://github.com/LLRiebel/MonoAlg3D_C-2023
Digital twinning pipeline to add fiber fields, transmurality, and apicobasal gradient in the mesh	Doste et al., 2026	https://github.com/rdoste/InSilicoHeartGen
Purkinje network generation tool	Berg et al. (2023)	https://github.com/bergolho/Shocker
ParaView	Kitware Inc	Version 5.13.2

### Method details

#### Multiscale electrophysiological modelling and simulation of the chronically infarcted human ventricles with Purkinje

Electrophysiological activity of the infarcted human ventricles was simulated from subcellular dynamics to the ECG as in Riebel et al. (2024), see Figure S8. Briefly, a healthy human biventricular geometry was reconstructed from MRI by Mincholé et al. (2019). Next, fibre orientations were introduced as described in Doste et al. (2019) and conduction velocity in fibre orientation calibrated to 65 cm/s, as observed clinically (Taggart et al., 2000). Sheet and sheet-normal conduction velocity was inferred (38 and 47 cm/s, respectively) and a Purkinje network created to reproduce the patient’s QRS complex (Camps et al., 2024), see Figure S8A.

Membrane kinetics were simulated with the state-of-the-art human ventricular cardiomyocyte and Purkinje cell ToR-ORd and Trovato models, respectively (Tomek et al., 2019; Trovato et al., 2020). Based on clinical observations (Boukens et al., 2015; Franz et al., 1987), biventricular electrophysiological gradients of 35 ms transmurally and 10 ms from apex to base were introduced by scaling key ionic current conductances (summarised in Table S1).

To model MI, three physiological scar geometries (available at https://doi.org/10.5281/zenodo.14699735) were introduced into the healthy geometry using an established algorithm (Cardone-Noott et al., 2014; Hill et al., 2016). Briefly, to model anteroseptal infarction, six landmarks were selected to model the path of the left anterior descending artery along the anterior and septal walls and the apex. By keeping these landmark coordinates constant but changing the volume of infarction in the mesh, we created three different geometries of anteroseptal infarction. In line with clinically reported scar sizes (Reindl et al., 2020; Spath et al., 2021), resulting scars and border zones composed 14%, 23%, and 27% of the ventricles (referred to as the small, medium, and large scar, respectively).

For remote zone and border zone electrophysiology, key ion channel conductances and time constants were scaled based on experimental observations as described by Zhou et al. (2024) and summarised in Table S2; resulting action potentials are shown in Figure S8A. The scar was modelled as electrically inactive but conducting (Rog-Zielinska et al., 2016). Hence, the membrane potential was initialised to -60 mV and allowed to change only through terms of diffusivity (Ringenberg et al., 2014). Electrophysiological changes caused remote zone conduction velocity to decrease to 49, 27, and 34 cm/s in fibre, sheet, and sheet-normal direction, respectively. Compared to the remote zone, conductivities were calibrated to achieve an approximately 60% and 80% reduced conduction velocity in the border and infarct zone, respectively (see Table S3), based on clinical observations (Aronis et al., 2020; Jamil-Copley et al., 2015). The resulting simulated ECG traces were compared to clinical data of post-MI subjects in our previous study (Riebel et al., 2024) and reproduced persistent ST-segment elevation, QT-interval prolongation, and normalised T-waves (see Figure S9A).

Multiscale, electrophysiological simulations were performed using the monodomain model with the finite volume GPU-enabled open-source solver MonoAlg3D (Berg et al., 2026; Sachetto Oliveira et al., 2018) with parameters listed in Table S4 and a customised version of the simulator available at https://github.com/LLRiebel/MonoAlg3D_C-2023.

#### Simulating injection of day 0, day 14, and rapid stem cell-derived cardiomyocytes

As shown in Figure S8B, to investigate transient automaticity-induced arrhythmias, we constructed a D14 hPSC-CM model by modifying ionic current conductances of the baseline Paci2020 model (Paci et al., 2020), which we consider the D0 model. For the D14 model, experimentally reported *in vivo* gene expression data (Marchiano et al., 2023) was consulted to derive ion channel scaling factors. As data to exactly relate ion channel conductance from gene expression is lacking, to qualitatively capture the cell’s maturation, we estimated the relative change in gene expression from Marchiano et al. (2023) and multiplied it by 0.25 to give a scaling factor for the corresponding ion channel conductance, as summarised in Table S5. This scaling factor of 0.25 accounts for the differences between the transcription and the translation level, considering factors such as translation efficiency, protein stability, and regulatory feedback (Stevens and Brown, 2013). Since large variability between spontaneous beating frequencies has been reported (He et al., 2003; Ma et al., 2011), we created an additional rapid beating hPSC-CM model by scaling the funny current, the sodium-calcium exchanger, and the sarco-endoplasmic reticulum calcium ATPase by 2.5 (resulting action potential biomarkers are shown in Table S6).

In 3D, cell injection was spatially modelled as in our previous study (Riebel et al., 2024), with meshes available at https://doi.org/10.5281/zenodo.14699735. Briefly, six injection locations were chosen within the infarct and maintained for the small, medium, and large scar. The probability for an adult ventricular cell to be replaced by an hPSC-CM decreased with increasing distance from the injection location, assuming low migration of hPSC-CMs as observed experimentally in chronic scars (Poch et al., 2022). The hPSC-CM distribution transmurally and in the infarct versus border zone was calibrated to experimental measurements (Poch et al., 2022) – particularly, hPSC-CMs were more likely to virtually settle in the subendocardium and in the infarct rather than the border zone. No hPSC-CMs were placed in the right ventricle or the remote zone. For each scar, the spatial hPSC-CM distribution was maintained across simulations including from D0 to D14. Conductivities were calibrated to achieve a 10 cm/s conduction velocity in hPSC-CMs at D0 and maintained for all other hPSC-CM models (see Table S3). This resulted in an increased conduction velocity of 14.5 cm/s at D14, similar to experimental observations (Hansen et al., 2018).

#### Identifying and optimizing anti-arrhythmic ionic targets

To identify suitable anti-arrhythmic targets, a sensitivity analysis of the spontaneously beating (i.e., unpaced) Paci2020 model was performed as previously described (Riebel et al., 2021). Briefly, each ion channel’s conductance was scaled by +/- 25% and the relative sensitivities on key action potential biomarkers including upstroke velocity, peak membrane potential, action potential duration at 90% repolarisation (APD_90_), maximum diastolic potential, and spontaneous beating frequency were computed as in Romero et al. (2009). The resulting relative sensitivities varied between -1 (strong negative correlation) and +1 (strong positive correlation).

Once targets to suppress automaticity were identified in the sensitivity analysis, we aimed to determine optimal combinations of ionic targets that also reduce re-entry susceptibility while preserving calcium transient amplitude. For this, a population of 1,458 D0 and D14 hPSC-CM models was created by simultaneously 1) blocking the funny current, 2) upregulating the inward rectifier potassium current’s conductance and maximum sodium-potassium pump current between 1 and 3 in steps of 0.25, and 3) upregulating the rapid delayed outward rectifier potassium current’s conductance between 1 and 5 in steps of 0.5. Spontaneously beating models were discarded and in the remaining population, an optimisation index incorporating both safety (measured by action potential duration) and efficacy (measured by calcium transient amplitude (CaT_Amp_)) was calculated as:

optimisationindex=

$$\frac{w_{safety}*\left(1-\frac{\left|{APD}_{90,target}-{APD}_{90}\right|}{{APD}_{90,target}}\right)+w_{efficacy}*\left(1-\frac{\left|{CaT}_{Amp,target}-{CaT}_{Amp}\right|}{{CaT}_{Amp,target}}\right)}{w_{safety}+w_{efficacy}}$$

Here, the weights w_safety_ and w_efficacy_ were set to 1.5 and 1, respectively, to prioritise safety. Target action potential duration can be used to create different hPSC-CM action potential models; here we aimed towards adult values to reduce repolarisation gradients. Target calcium transient amplitude was always set to the maximum calcium transient amplitude achieved across the population (excluding any spontaneously beating models).

Modifying ionic current expression in hPSC-CMs has been achieved through gene editing techniques before injection (Marchiano et al., 2023). Hence, we combined optimisation indices at D0 and D14 for each ionic scaling combination, using also the maximum optimisation indices index_D0,max_ and index_D14,max_:

$$combined\;optimisation\;index=\left(\frac{{index}_{D0}}{{index}_{D0,\max}}+\frac{{index}_{D14}}{{index}_{D14,\max}}\right)/\;2$$

#### Stimulation protocols to simulate sinus rhythm and quantify arrhythmic risk

The adult ventricular cardiomyocyte ToR-ORd model (Tomek et al., 2019), with remote, border, or infarct zone remodelling applied (Zhou et al., 2024), and the Purkinje cell Trovato2020 model (Trovato et al., 2020) were paced in virtual isolation at 1 Hz until steady state for 500 and 1,000 s, respectively, and then introduced into the biventricular model. As full coupling and therefore direct pacing of the hPSC-CMs in the ventricles could not be assumed, the hPSC-CM Paci2020 model (Paci et al., 2020), including D0, D14, rapid, and optimised variations, were simulated in isolation under spontaneous (i.e., unpaced) conditions for 1,000 s before being introduced into the ventricles. The biventricular model was then paced for three 1 Hz beats, applied at the top 25 elements of the bundle of His.

To assess automaticity-induced arrhythmias, biventricular models with either D0, rapid D0, D14, or rapid D14 hPSC-CMs were simulated without pacing for two seconds. If spontaneous beats occurred, ten 1 Hz beats were simulated to assess whether automaticity-induced arrhythmias would emerge under sinus pacing. To quantify re-entry burden, an S1-S2 protocol was employed: after the initial three 1 Hz beats (i.e., S1 stimuli), an S2 stimulus was applied in 15 different border zone locations between 350 to 570 ms after the S1. The different S2 locations were selected manually around the core of the small, medium, and large scars. Re-entry risk was measured as the vulnerable window (i.e., the range of S1-S2 intervals for which arrhythmias could be induced); considering S1-S2 intervals were increased in 20 ms increments, vulnerable windows were a multiple of 20.

This set up resulted in 1,458 single cell simulations to determine optimal ionic target combinations (9 scalings for the inward rectifier potassium current’s conductance ^∗^ 9 scalings of the maximum sodium-potassium pump current ^∗^ 9 scalings for the rapid delayed outward rectifier potassium current’s conductance ^∗^ 2 hPSC-CM models, i.e., D0 and D14) and 1,806 biventricular simulations (12 S1-S2 intervals ^∗^ 15 S2 locations ^∗^ (3 scars before cell injection + 3 scars at D0 + large scar at D14 + large scar with 3 (2 at D0, 1 at D14) optimised hPSC-CM models) + 6 simulations of spontaneous activity).

#### Verification, validation, and uncertainty quantification

To establish trust in our modelling and simulation framework to mechanistically investigate cell therapy in chronic MI, we considered verification, validation, and uncertainty quantification principles (Musuamba et al., 2021; Viceconti et al., 2021). Verification of the monodomain solver was provided through benchmark tests (Berg et al., 2026; Sachetto Oliveira et al., 2018) and comparison to cell model solvers (Riebel et al., 2024).

Validation of the cellular models was demonstrated through incorporation of biophysiological knowledge and experimental evidence of human cardiac cells in the original publications (Paci et al., 2020; Tomek et al., 2019; Trovato et al., 2020). Furthermore, predictive accuracy of these models has been established through *in silico* drug trials on contractility as well as arrhythmogenicity (Paci et al., 2021; Passini et al., 2021; Trovato et al., 2022, 2025). Electrophysiological and structural remodelling post-MI was informed through experimental and clinical evidence as outlined above. Additionally, trust in the biventricular post-MI model was provided through comparison of simulated ECGs with clinical data (Riebel et al., 2024; Zhou et al., 2024).

In addition to previous sensitivity and convergence analyses (Riebel et al., 2024), we carried out further uncertainty quantification into the effects of steady state pacing duration and ionic concentrations on hPSC-CM action potentials at D0 and D14, as shown in Figures S10 and S11. These analyses showed small changes (less than 10%) in action potential duration at 90% repolarisation.

## Supplemental information

Document S1. Figures S1–S11 and Tables S1–S6

Document S2. Article plus supplemental information
