# Supplementary material for: In silico optimization of regenerative cell therapy in the infarcted human ventricles to mitigate arrhythmic burden
Source: Stem Cell Reports. 2026 Jul 9;21(8):103007. doi: 10.1016/j.stemcr.2026.103007 (PMC13476901; doi:10.1016/j.stemcr.2026.103007)
Supplement: Supplementary file 1 [file mmc1.pdf]

Stem Cell Reports, Volume *21*

Supplemental Information

In silico optimization of regenerative cell therapy in the infarcted human ventricles to mitigate arrhythmic burden

Leto Luana Riebel, Zhinuo Jenny Wang, Xin Zhou, Lucas Arantes Berg, Cristian Trovato, and Blanca Rodriguez

Supplementary Material

Contents

S1. Arrhythmic risk after virtual cell delivery 1
S2. Identifying anti-arrhythmic targets for hPSC-CMs 2
S3. Human electrophysiological modelling and simulation of chronic MI 8
S4. Modelling and simulation of cell therapy 9
S5. Verification, validation, and uncertainty quantification... 12
Supplemental References 14

S1. Arrhythmic risk after virtual cell delivery

Figure S1 below supplements Figure 1 in the main text to highlight that no automaticity-induced arrhythmias were observed after virtual injection of the baseline hPSC-CMs at D0 or D14 as well as the rapidly beating hPSC-CMs at D0.

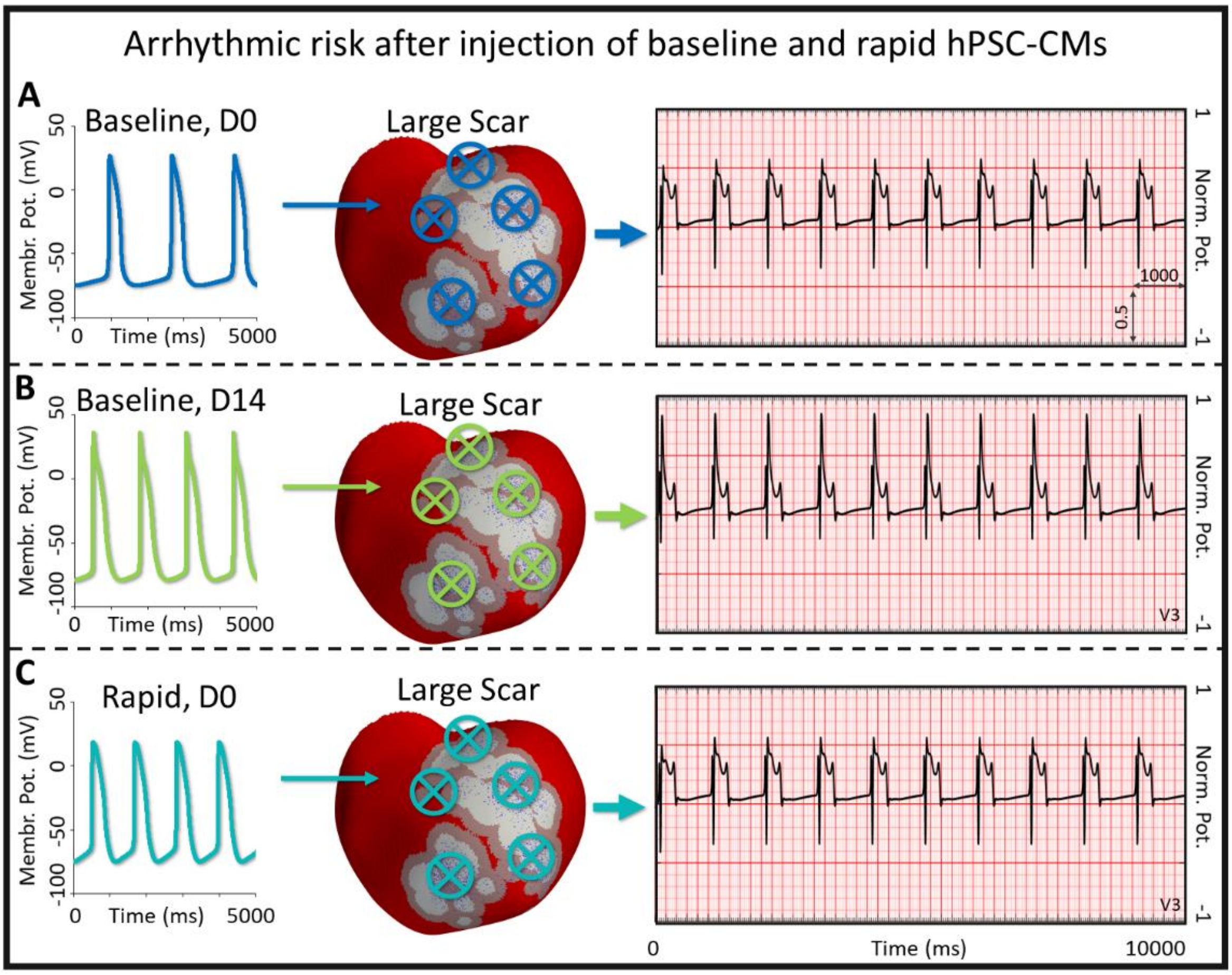

Figure S1. Absence of automaticity-induced arrhythmia burden after virtual injection of baseline hPSC-CMs at D0 (panel A) and D14 (panel B) as well as rapid hPSC-CMs at D14 (panel C). Simulations were performed in a human biventricular model

with Purkinje and a large synthetic anteroseptal MI scar. Left: AP traces of the different hPSC-CM models beating spontaneously in virtual isolation. Middle: five out of six virtual hPSC-CM injection locations highlighted with crosses in the biventricular mesh (the remaining one is located in the septum). Right: ECG lead V3 traces after virtual cell injection during 1 Hz stimulation at the bundle of His (note the absence of arrhythmias). Related to main text Figure 1.

S2. Identifying anti-arrhythmic targets for hPSC-CMs

As shown in Figure S2 below, sensitivity analyses showed the D0 model's spontaneous beating rate most sensitive to p_{NaK} (maximum sodium potassium pump current). Scaling p_{NaK} by 0.7 and 0.85 was sufficient to abolish spontaneous activity in the D0 and D14 phenotype, respectively.

Sensitivity analysis of unpaced hPSC-CMs

Parameter	MDP	dV/dt_{max}	V_{peak}	APD_{90}	Frequency
$V_{max,up}$		0.67			0.51
G_{CaL}		0.84	0.89	1.00	
k_{NaCa}			0.26		0.37
G_{NaF}		0.99			
G_{NaL}					
P_{NaK}		-1.00	1.00	0.25	-1.00
G_{to}					
G_{Kr}			-0.29	-0.90	
G_{Ks}					
G_{K1}	1.00	0.40	-0.41	-0.74	
G_f		-0.46		-0.29	0.35

Relative sensitivity < ±0.25 | Strong negative relation | Strong positive relation

Figure S2. Sensitivity analysis of the spontaneous computational D0 (i.e., baseline Paci2020 (Paci et al., 2020)) hPSC-CM model to changes in ionic current conductances. Shown are relative sensitivities (1: strong positive relation, -1: strong negative relation), calculated as in Romero et al. (2009). Conductances were scaled by +/- 25% and hPSC-CMs simulated to beat spontaneously for 1,000 s.

Figure S3, Figure S4, and Figure S5 below show ionic current and concentration traces underlying hPSC-CMs at D0 and D14 with and without scaling p_{NaK}, G_{K1}, and G_f. Note, in the hPSC-CM model with reduced p_{NaK} at D14 (Figure S4 A), the model had to be paced for 2000 rather than 1000 beats to reach steady state.

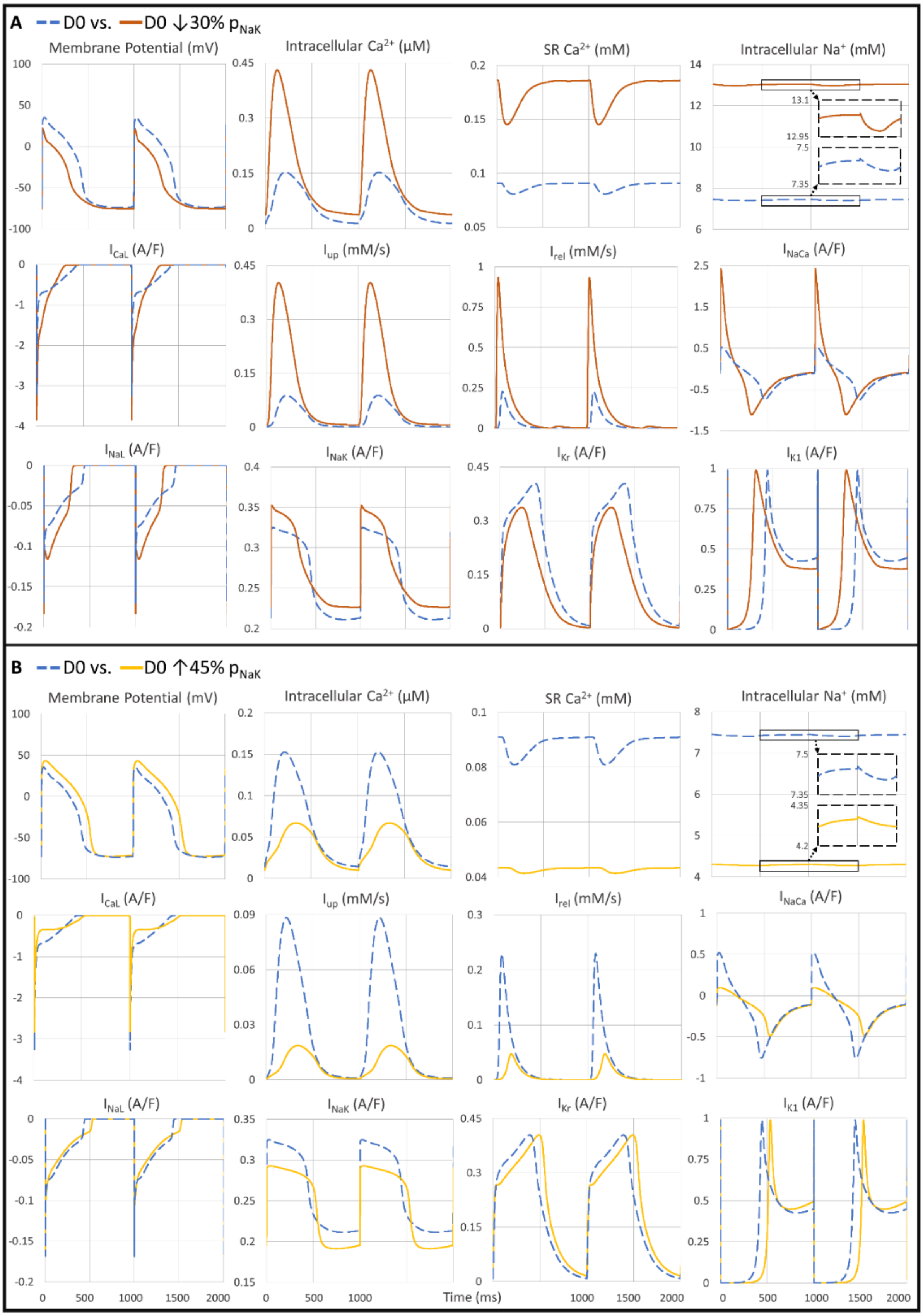

Figure S3. Simulated action potential and ionic current traces of baseline D0 hPSC-CMs in isolation after 1,000 paced 1 Hz beats. A) With and without downregulation of p_{NaK} (maximum Na^+-K^+ pump current (I_{NaK})). B) With and without upregulation of p_{NaK}. Further shown are intracellular and SR Ca^{2+} concentrations, intracellular Na^+ concentrations, I_{CaL}: L-type Ca^{2+} current, I_{up}: SERCA pump current, I_{rel}: SR Ca^{2+} release current, I_{NaCa}: Na^+-Ca^{2+} exchanger current, I_{NaL}: late Na^+ current, I_{Kr}: rapid delayed outward K^+ current, and I_{K1}: inward rectifier K^+ current. Related to main text Figure 4.

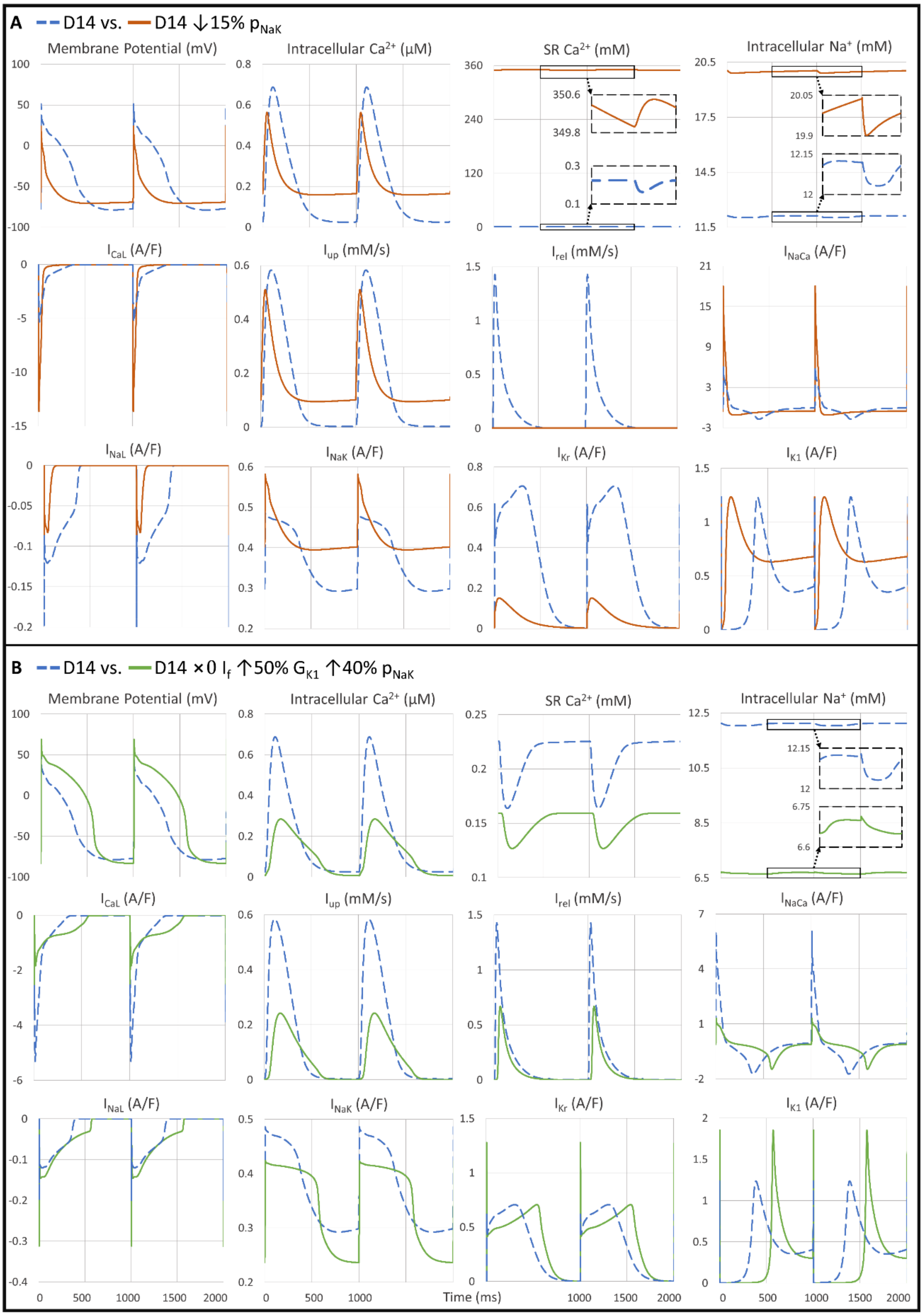

Figure S4. Simulated action potential and ionic current traces of baseline D14 hPSC-CMs in isolation after 1,000 paced 1 Hz beats. A) With and without downregulation of p_{NaK} (maximum Na^{+}-K^{+} pump current (I_{NaK})); note that 2,000 beats were needed to achieve steady state in the D14 model with low p_{NaK} with the last beat shown here. B) With and without upregulation of p_{NaK} plus full block of the funny current (I_f) and upregulation of the inward rectifier K^{+} current's (I_{K1}) conductance. Further shown are intracellular and SR Ca^{2+} concentrations, intracellular Na^{+} concentrations, I_{CaL}: L-type Ca^{2+} current, I_{up}: SERCA pump current, I_{rel}: SR Ca^{2+} release current (effectively 0 when reducing p_{NaK}), I_{NaCa}: Na^{+}-Ca^{2+} exchanger current, I_{NaL}: late Na^{+} current, and I_{Kr}: rapid delayed outward K^{+} current. Related to main text Figure 4.

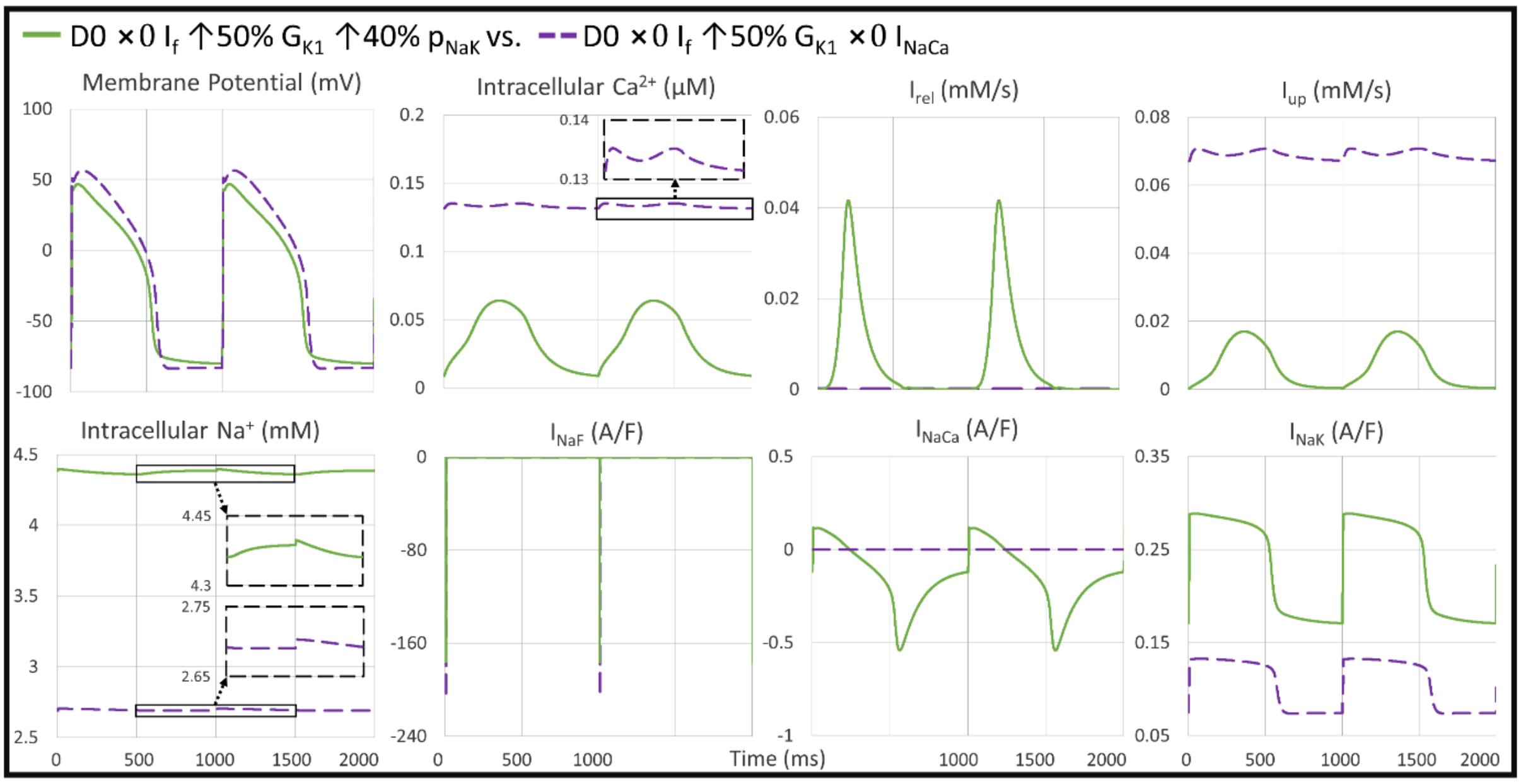

Figure S5. Simulated action potential and ionic current traces after 1,000 paced 1 Hz beats of baseline D0 hPSC-CMs with full block of the funny current (I_f) and upregulation of the inward rectifier K^+ current's (I_{K1}) conductance plus either upregulation of p_{NaK} (maximum Na^+-K^+ pump current (I_{NaK})), green solid traces, or full block of the Na^+-Ca^{2+} exchanger current (I_{NaCa}, as suggested by Marchiano et al. (2023)), purple dotted traces. Further shown are intracellular Ca^{2+} and Na^+ concentrations, I_{rel}: SR Ca^{2+} release current (effectively 0 for I_{NaCa} block), I_{up}: SERCA pump current, and I_{NaF}: fast Na^+ current. Related to main text Figure 4.

From the results presented in Figure S6, we chose the best performing ionic target combination. For Figure S7, we chose the ionic scaling combination with the third best result: fully blocking I_f together with upregulating G_{K1} by 1.25, p_{NaK} by 1.75, and G_{Kr} by 5, referred to in the text as "optimised" hPSC-CMs. We eliminated the top performing combination because it showed small beat-to-beat variations. We further discarded the second-best combination as it suggested that no G_{K1} scaling was necessary, which may be specific to our virtual cell types. As the differences in action potential duration at 90% repolarisation (APD_{90}) and calcium transient amplitude between the second and third-best options were only 2 ms and 0.001 µM, respectively, we finally chose to proceed with the third option that maintained G_{K1} scaling at 1.25. The optimised cells at D0 and D14 had an APD_{90} of 169 ms and 230 ms, respectively.

	PNaK	1.5	1.75	2	2.25	2.5	2.75	3
GK1	GKr							
	1							
	1.5							
	2							
	2.5		0.86	0.84	0.83	0.81	0.81	0.80
1	3		0.872	0.86	0.85	0.84	0.84	0.83
	3.5		0.85	0.84	0.84	0.83	0.82	0.82
	4		0.80	0.79	0.78	0.78	0.77	0.77
	4.5	0.77	0.75					
	5							
	1							
	1.5							
	2							
	2.5		0.86	0.83	0.82	0.81	0.80	0.80
1.25	3		0.872	0.86	0.85	0.84	0.84	0.83
	3.5		0.86	0.84	0.83	0.83	0.83	0.82
	4		0.80	0.79	0.78	0.78	0.77	0.77
	4.5		0.75					
	5							
	1							
	1.5							
	2	0.79						
	2.5		0.86	0.84	0.82	0.81	0.80	0.79
1.5	3		0.874	0.86	0.85	0.84	0.84	0.83
	3.5		0.86	0.84	0.84	0.83	0.83	0.82
	4			0.79	0.78	0.78	0.78	0.77
	4.5			0.75				
	5							
	1							
	1.5							
	2	0.80	0.75					
	2.5		0.86	0.84	0.82	0.81	0.80	0.79
1.75	3			0.86	0.85	0.84	0.84	0.84
	3.5			0.84	0.84	0.83	0.83	0.83
	4				0.79	0.78	0.78	0.77
	4.5							
	5							
	1							
	1.5							
	2		0.75					
	2.5			0.84	0.82	0.81	0.80	0.79
2	3				0.85	0.84	0.84	0.84
	3.5					0.83	0.83	0.83
	4					0.78	0.78	0.77
	4.5							
	5							
	1							
	1.5							
	2							
	2.5							
2.25	3							
	3.5							
	4							
	4.5							
	5							
	1							
	1.5							
	2							
	2.5							
2.5	3							
	3.5							
	4							
	4.5							
	5							

Figure S6. Optimisation index for baseline D0 and D14 hPSC-CMs with a target APD_{90} of 330 ms, computed for full block of the funny current (I_f) and different combinations of upscaling the inward rectifier K^+ current conductance (G_{K1}), the maximum Na^+-K^+ pump current (P_{NaK}), and the rapid delayed outward rectifier K^+ current conductance (G_{Kr}). Dark green indicates a high scoring. Scores below 0.75 are not numbered. Note, no score was computed if the cells were still beating spontaneously or could not be paced. Highlighted is the best performing scaling combination.

GK1	GKr \ PNaK	1.5	1.75	2	2.25	2.5	2.75	3
1	1							
	1.5							
	2							
	2.5							
	3							
	3.5							
	4							
	4.5	0.87	0.84	0.82	0.81	0.80	0.79	0.79
	5	0.95	0.9270	0.92	0.91	0.90	0.89	0.89
1.25	1							
	1.5							
	2							
	2.5							
	3							
	3.5							
	4							
	4.5		0.83	0.81	0.80	0.79	0.78	0.77
	5		0.9266	0.91	0.90	0.89	0.88	0.87
1.5	1							
	1.5							
	2							
	2.5							
	3							
	3.5							
	4							
	4.5			0.81	0.79	0.78	0.77	0.76
	5			0.90	0.89	0.88	0.87	0.87
1.75	1							
	1.5							
	2							
	2.5							
	3							
	3.5							
	4							
	4.5				0.79	0.78	0.77	0.76
	5				0.89	0.88	0.87	0.86
2	1							
	1.5							
	2							
	2.5							
	3							
	3.5							
	4							
	4.5						0.77	0.76
	5							0.86
2.25	1							
	1.5							
	2							
	2.5							
	3							
	3.5							
	4							
	4.5							
	5							
2.5	1							
	1.5							
	2							
	2.5							
	3							
	3.5							
	4							
	4.5							
	5							

Figure S7. Optimisation index for baseline D0 and D14 hPSC-CMs with a target APD_{90} of 170 ms, computed for full block of the funny current (I_f) and different combinations of upscaling the inward rectifier K^+ current conductance (G_{K1}), the maximum Na^+-K^+ pump current (p_{NaK}), and the rapid delayed outward rectifier K^+ current conductance (G_{Kr}). Dark green indicates a high score. Scores below 0.75 are not numbered. Note, no score was computed if the cells were still beating spontaneously or could not be paced. Highlighted are the top three performing scalings with the selected one in yellow.

S3. Human electrophysiological modelling and simulation of chronic MI

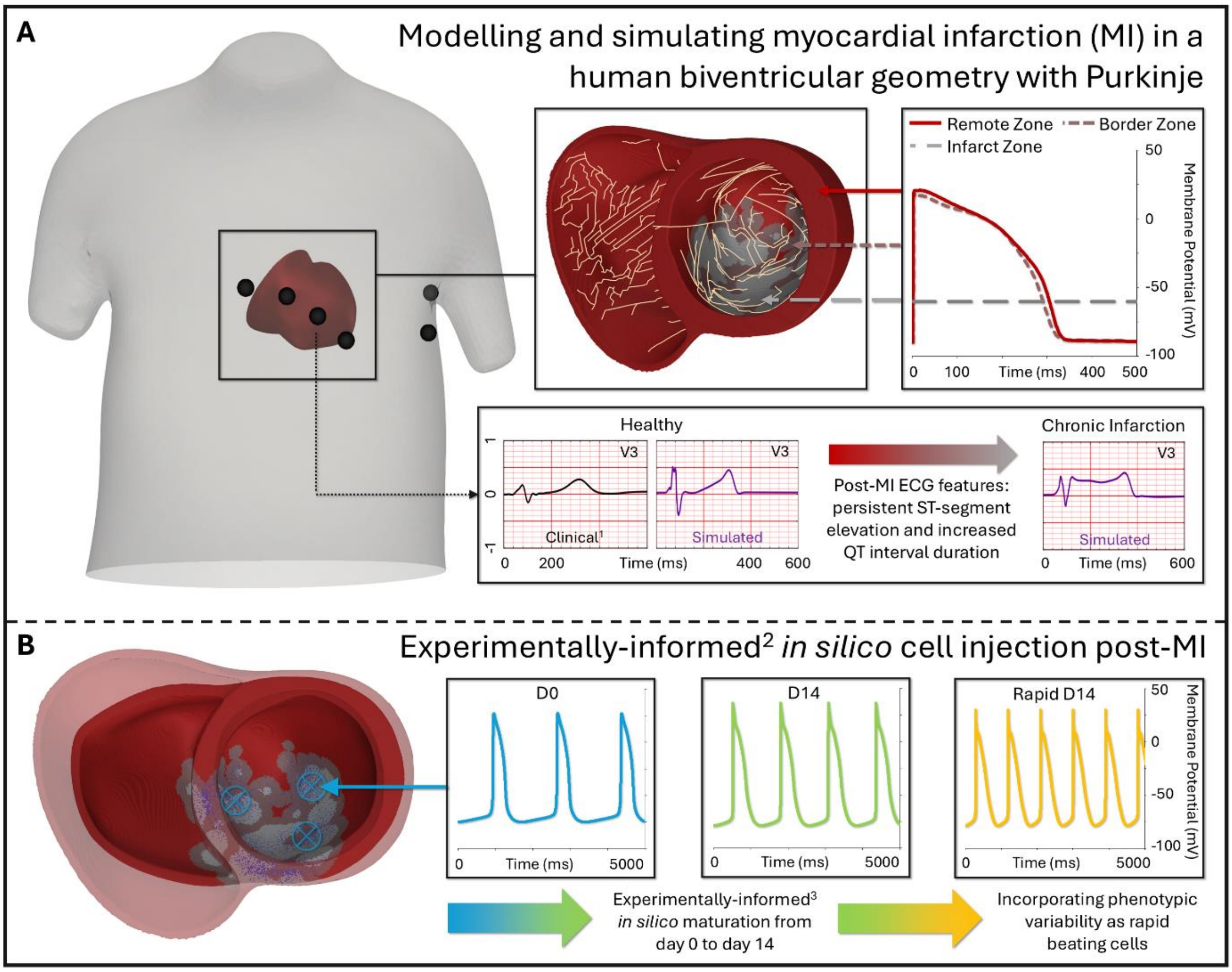

Figure S8. Multiscale modelling and simulation of human ventricular electrophysiology in A) chronic MI and B) cell therapy post-MI using a D0, D14, and rapid beating hPSC-CM model. [1]Biventricular geometry and ECG recordings from Mincholé et al. (2019). [2]Spatial distribution of hPSC-CMs was informed by Poch et al. (2022). [3]Maturation from D0 to D14 was simulated based on in vivo gene expression data from Marchiano et al. (2023).

Table S1 below summarises ionic scaling factors that were applied to model epicardial (based on the baseline endocardial) human ventricular cardiomyocytes and an apicobasal gradient.

Table S1. Ionic current scaling factors to produce I) epicardial human ventricular cardiomyocytes from the baseline endocardial phenotype as in [1]Tomek et al. (2019, 2020) resulting in a transmural action potential duration gradient of 35 ms, and II) a ventricular apex to base gradient as established by [2]Okada et al. (2011) and Mincholé et al. (2019) resulting in an apicobasal repolarisation gradient of 10 ms. V_m denotes the membrane voltage.

**Model parameter**	**Epicardial cell type[1]**	**Apex to base gradient[2]**
G_{NaL}	*0.6	
P_{NaK}	*0.9	
G_{to}	*2.0	
$delta_{epi}$	$= 1 - \frac{0.95}{1+e^{\frac{V_m+70}{5}}}$	
G_{Kr}	*1.3	
G_{Ks}	*1.4	*gradient between 0.2 at the base and 5.0 at the apex
G_{K1}	*1.2	

G_{Kb}	*0.6	
G_{NaCa}	*1.1	
P_{Ca}	*1.2	
J_{upnp}	*1.3	
J_{upp}	*1.3	
$cmdn_{max}$	*1.3	

Table S2 below outlines remodelling of cellular ion channel conductances and time constants that was applied to simulate chronically infarcted human cardiomyocytes in the infarct, border, and remote zone.

Table S2. Border and remote zone remodelling as established by Zhou et al. (2024). Infarct zone modelling as suggested by [7]Ringenberg et al. (2014). Supporting experimental data from [1]Valdivia et al. (2005), [2]Hegyi et al. (2018), [3]Jiang et al. (2002), [4]Høydal et al. (2018), [5]Maier et al. (2003), and [6]Hoch et al. (1999).

**Model Parameter**	**Remote Zone**	**Border Zone**	**Infarct Zone**
V_m (set not scaled)			-60 mV[7]
G_{Na}	0.43[1]	0.43[1]	
G_{NaL}	1.413[2]	1.275[2]	
G_{Kr}	0.87[2]	0.89[2]	
G_{K1}		0.76[2]	
G_{KCa}	2[2]	2[2]	
P_{Ca}		0.7[2]	
J_{up}	0.4[3,4]	0.4[3,4]	
tau_{relp}	6[5]	6[5]	
aCaMK	1.5[6]	1.5[6]	
G_{ClCa}	1.25[2]	1.25[2]	

S4. Modelling and simulation of cell therapy

Table S3 below shows conduction velocities that were achieved in the adult myocardium as well as the virtually injected hPSC-CMs.

Table S3. Conduction velocities of adult ventricular tissue and hPSC-CMs. Using conductivity tensors of (2.60, 1.05, 1.60) mS/cm in the remote zone, (0.60, 0.35, 0.45) mS/cm in the border and infarct zone, and (0.2, 0.2, 0.2) mS/cm in hPSC-CMs. Measured in a 20x7x3 cm tissue slab stimulated in the bottom corner, as suggested in benchmark tests by Niederer et al. (2011). Note, hPSC-CM connectivity was modelled as isotropic.

**Cell type**		**Conduction Velocity (cm/s)**		
		Fibre	Sheet	Normal
Adult ventricular myocardium	Remote Zone	49	27	34
	Border Zone	19	12	15
	Infarct Zone	9	7	8
Stem cell-derived cardiomyocytes	D0 (baseline Paci2020 model)	10		
	Rapid D0	9		
	D0 with ×0 I_f ↑50% G_{K1} ↑75% p_{NaK} ↑200% G_{Kr}	12.5		

D0 with ×0 I_f ↑25% G_{K1} ↑75% p_{NaK} ↑400% G_{Kr}	12.5
D14	14.5
Rapid D14	12.5
D14 with ×0 I_f ↑25% G_{K1} ↑75% p_{NaK} ↑400% G_{Kr}	18

Simulated ECGs from before and after virtual cell injection are shown in Figure S9 below. Delivery of hPSC-CMs at D0 caused ST-elevation in leads V1 to V3 as well as T-wave depression in leads V4 and V5. Virtual injection of hPSC-CMs at D14 increased QRS fragmentation and J-point elevation in leads V1 to V3 as well as depressed T-waves in leads V4 and V5. Furthermore, as conduction velocity increased in hPSC-CMs from D0 to D14 (see Table S3), activation of the scar improved, which is visible in the ECG as reduced QRS-prolongation. Virtual delivery of optimised hPSC-CMs at D0 and D14 further depressed the T-wave, also in leads V2 and V3.

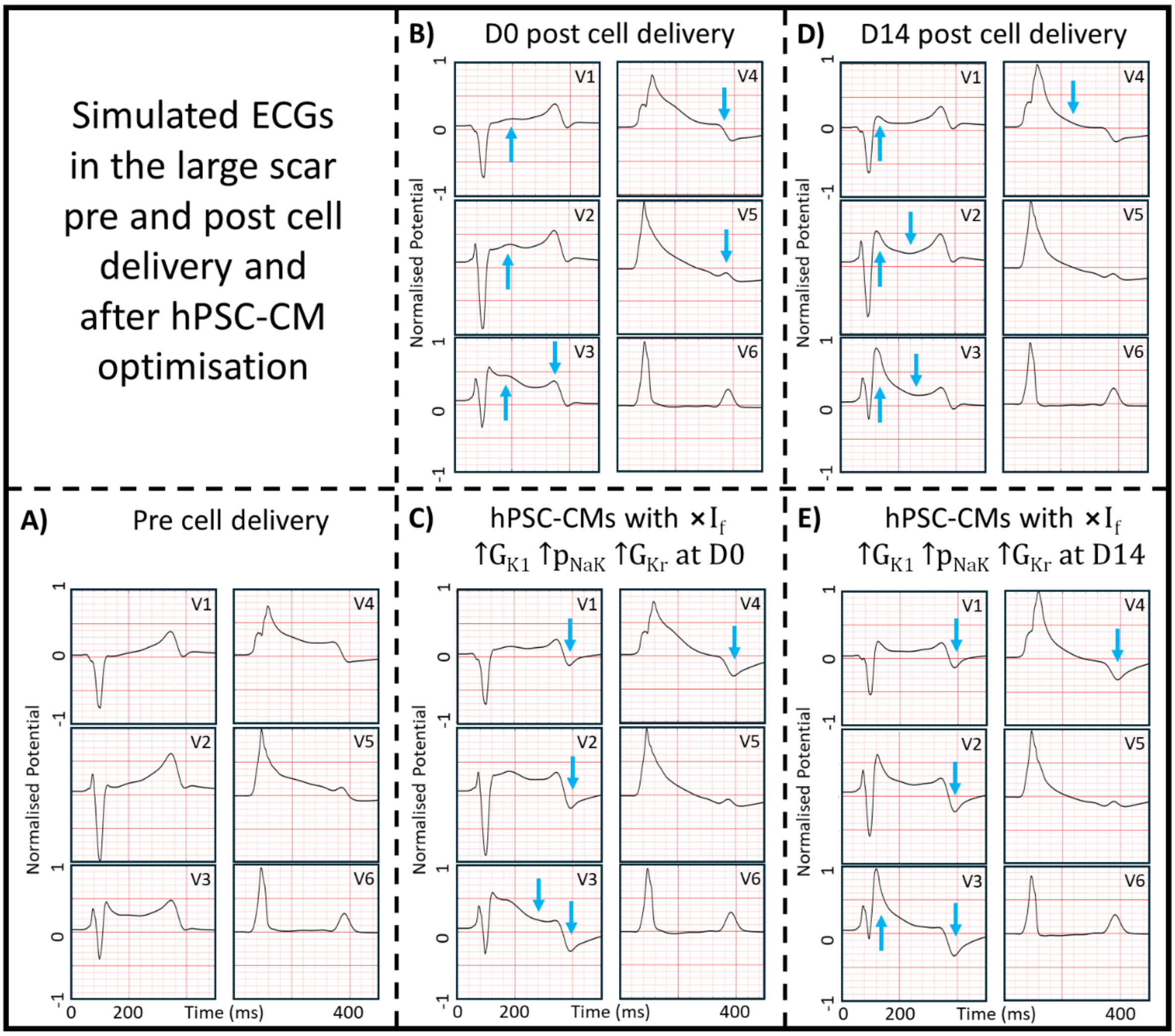

Figure S9. Simulated ECG before and at D0 and D14 after virtual delivery of stem cell-derived cardiomyocytes (hPSC-CMs) before and after optimisation (i.e., I_f block and G_{K1}, p_{NaK}, and G_{Kr} upregulation). Blue arrows indicate prominent changes from panels A) to B), B) to C), B) to D), and D) to E).

Table S4 below details input parameters for the Monodomain solver MonoAlg3D (Berg et al., 2026; Sachetto Oliveira et al., 2018).

Table S4. Parameter files to configure MonoAlg3D (Berg et al., 2026; Sachetto Oliveira et al., 2018) for 3D simulations.

MonoAlg3D Parameter	ToR-ORd	Paci2020	Trovato
Space adaptivity	false		
PDE timestep	0.01		
PDE solved in the…	GPU		
ODE time step	0.01		
ODE solver method	Rush-Larsen + Euler		
ODE adaptivity	false		
ODE solved in the…	GPU		CPU
Linear system solver gradient function	Biconjugate		Conjugate
Linear system solver tolerance	1e-12		1e-16

Table S5 and Table S6 below introduce scaling factors for ionic current conductances to simulate baseline and rapid hPSC-CM phenotypes at D0 and D14 and further show their resulting action potential biomarkers.

Table S5. Ion channel conductance scaling factors to produce D14 (based on experimental gene expression data from Marchiano et al. (2023, Figure 1F)) and rapid beating hPSC-CMs using Paci2020 (Paci et al., 2020) as the baseline D0 model.

Model Parameter	D14	Rapid D0	Rapid D14
G_f	1.25	2.50	3.125
G_{K1}	1.25		1.250
G_{to}	1.50		1.500
G_{Ks}	2.00		2.000
G_{Kr}	1.75		1.750
G_{NaF}	2.50		2.500
k_{NaCa}	2.00	2.50	5.000
G_{CaL}	3.00		3.000
$V_{max,up}$		2.50	2.500

Table S6. AP biomarkers for D0, D14, and rapid hPSC-CM phenotypes after 1,000 seconds of spontaneous activity using Paci2020 (Paci et al., 2020). DDR: diastolic depolarisation rate, MDP: maximum diastolic potential, dV/dt_{max}: maximum upstroke velocity, $V_{m,peak}$: peak V_m, APD_{90}: APD at 90% repolarisation, F: spontaneous beating frequency..

hPSC-CM phenotype	DDR (V/s)	MDP (mV)	dV/dt_{max} (V/s)	$V_{m,peak}$ (mV)	APD_{90} (mV)	F (bpm)
D0	0.01	-74.87	20.54	27.14	402.42	35
Rapid D0	0.02	-74.90	26.49	18.76	424.27	52
D14	0.02	-78.79	172.26	36.53	446.31	47
Rapid D14	0.03	-78.57	168.01	29.94	414.57	66

S5. Verification, validation, and uncertainty quantification

To quantify uncertainty, we carried out further sensitivity analyses. First, as mentioned in the stimulation protocols in the main text, biventricular simulations used the unpaced hPSC-CM models after 1,000 s as a starting state. We chose unpaced rather than paced activity, as coupling between hPSC-CMs and surrounding tissue, particularly within the scar, may be incomplete impacting their pacing. However, in isolation, the optimised hPSC-CMs' spontaneous beating was suppressed and only a few APs occurred at the start of the unpaced 1,000 s. This leads to uncertainty regarding the models' action potential after possible pacing in the ventricles, especially as we changed I_{NaK}, which may substantially affect ionic concentrations. Hence, we compared the action potentials of optimised hPSC-CMs (full I_f block and 25%, 75%, and 400% I_{K1} I_{NaK}, and I_{Kr} upregulation, respectively) after 1,000 s of unpaced activity followed by 3 paced beats (mimicking the biventricular simulation setting) versus 1,000 paced beats. As shown in Figure S10 A) below, this produced small changes in the D0 model (APD_{90} difference of approximately 10 ms). At D14 (panel B), the calcium transient amplitude increased about 3-fold over time and the action potential shape changed more substantially (reduced peak and steeper repolarisation). However, APD_{90} changed only from 230 to 245 ms with increased pacing duration.

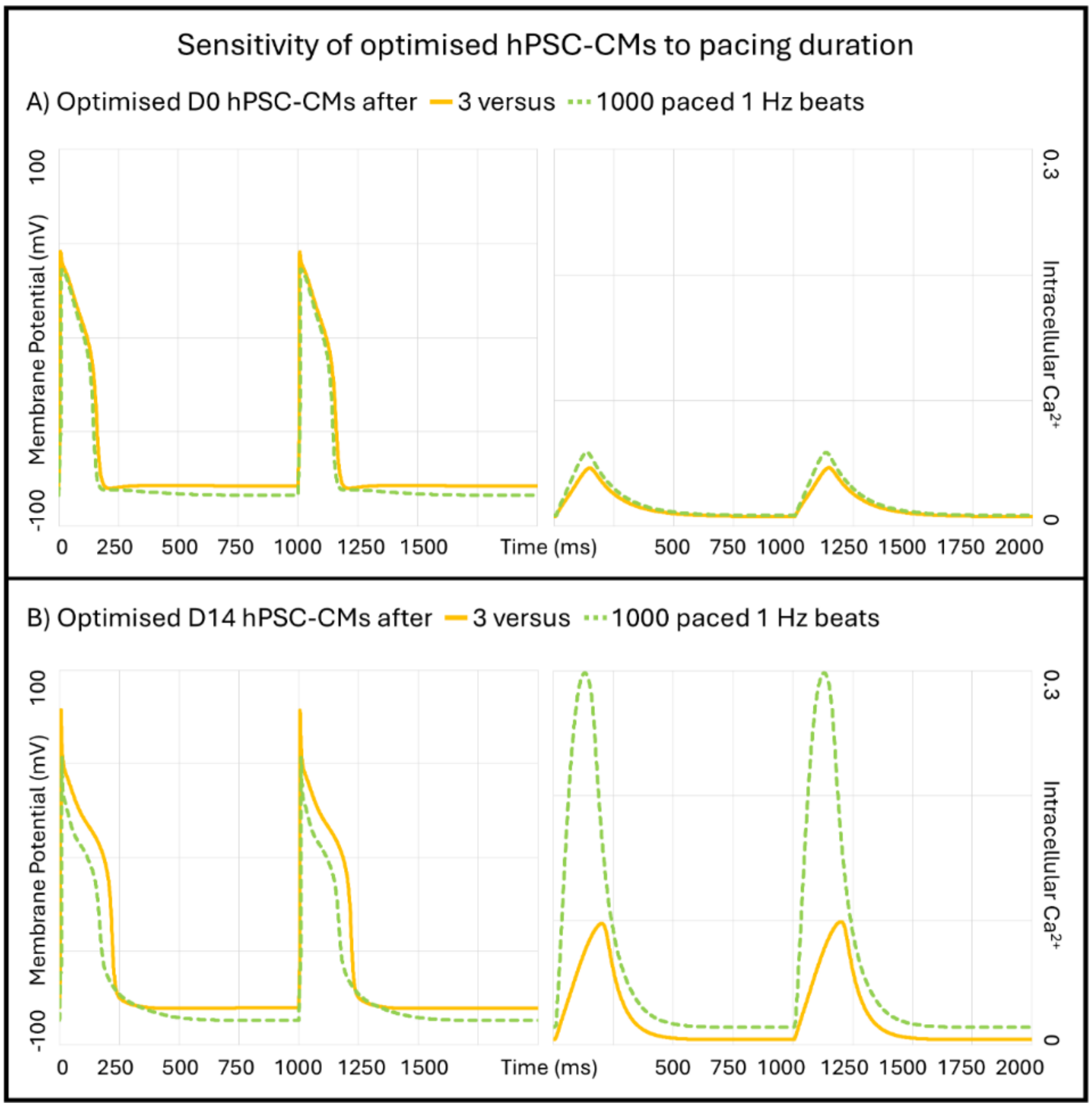

Figure S10. Sensitivity of optimised stem cell-derived cardiomyocytes (hPSC-CMs) to pacing duration. Isolated hPSC-CMs at A) D0 and B) D14 with full block of the funny current, 25% upregulation of the inward rectifier K^+ current's conductance, 75%

upregulation of maximum Na^+-K^+ pump current, and 400% upregulation of the rapid delayed outward rectifier K^+ current's conductance. Simulated in single cell for 1,000 s of unpaced activity followed by 3 s (solid yellow traces) or 1,000 s (dashed green traces) of 1 Hz pacing.

Next, we investigated the sensitivity to differences in ionic concentrations, as these differ slightly in the hPSC-CM Paci2020 model and the adult ventricular cardiomyocyte ToR-ORd model. Specifically, extracellular K^+ and Na^+ concentrations are 151 versus 140 and 5.4 versus 5.0 mM in the Paci2020 versus ToR-ORd model, respectively. Considering that ionic concentrations are variable spatially and over time, in our simulations, we did not change these, also allowing for comparability to previous studies. Comparison on the effect of changing the Paci2020 concentrations to the ToR-ORd values, shown in Figure S11, showed small changes in the spontaneous beating frequency (35 bpm before versus 36 bpm after changing concentrations) and the paced APD_{90} (455 before versus 440 ms after changing concentrations).

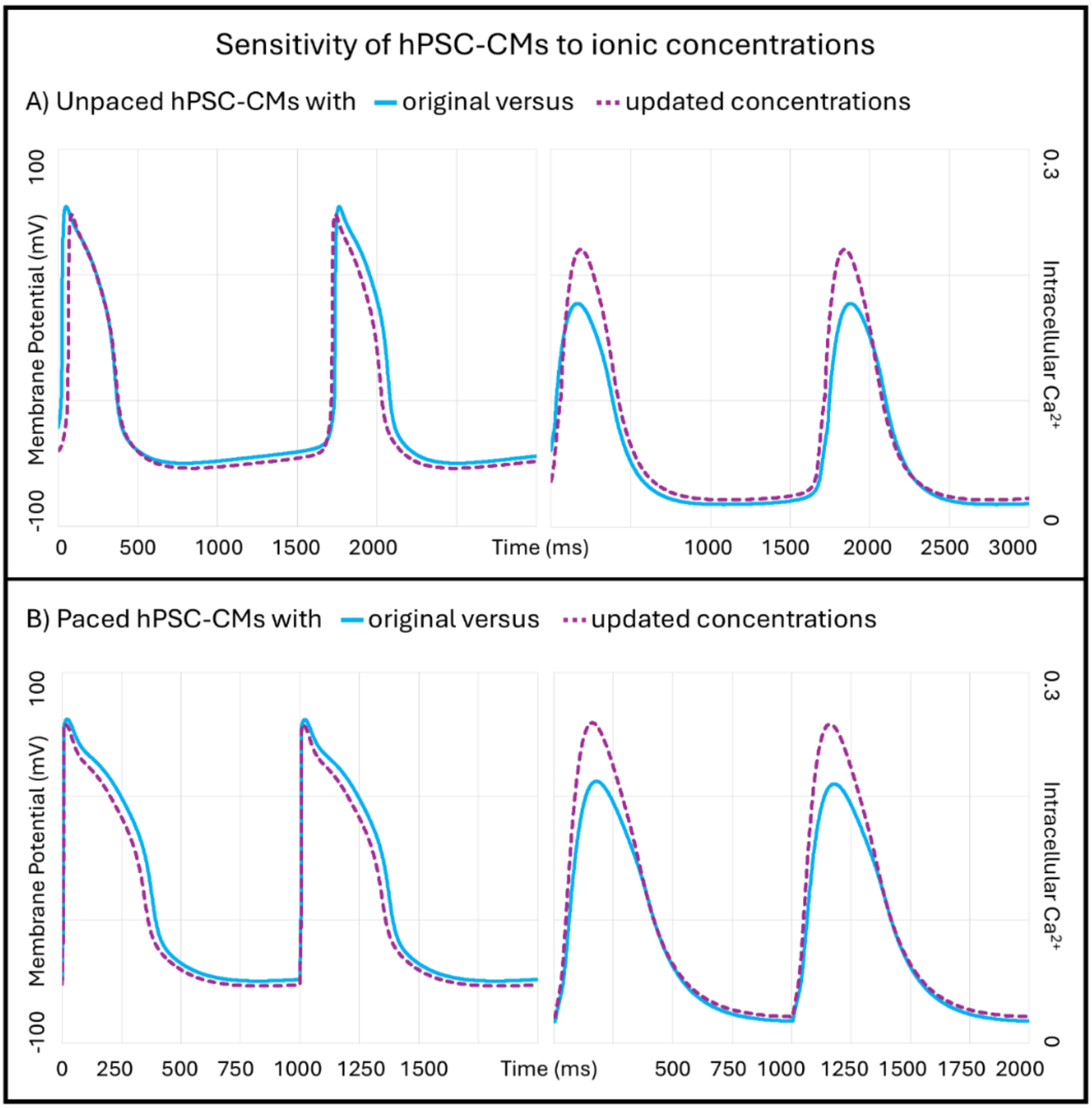

Figure S11. Sensitivity of D0 hPSC-CMs in isolation to ionic concentrations. Original hPSC-CM Paci2020 model (Paci et al., 2020) with extracellular Na^+ of 151 mM and extracellular K^+ of 5.4 mM (solid blue traces) or using ionic concentrations from the adult ventricular ToR-ORd model (Tomek et al., 2019, 2020), i.e., extracellular Na^+ of 140 mM and extracellular K^+ of 5 mM (dashed purple traces). A) 1,000 s of spontaneous beating were simulated and B) followed by 3 s of 1 Hz pacing.

Supplemental References

Berg, L. A., Oliveira, R. S., Camps, J., de Lima, L. M. R., de Oliveira Campos, J., Wang, Z. J., Doste, R., Bueno-Orovio, A., dos Santos, R. W., & Rodriguez, B. (2026). Toward cardiac electrophysiology digital twins with an efficient open source scalable solver on GPU clusters. *Scientific Reports*, *16*(1), 9619. https://doi.org/10.1038/s41598-025-33709-w

Hegyi, B., Bossuyt, J., Griffiths, L. G., Shimkunas, R., Coulibaly, Z., Jian, Z., Grimsrud, K. N., Sondergaard, C. S., Ginsburg, K. S., Chiamvimonvat, N., Belardinelli, L., Varró, A., Papp, J. G., Pollesello, P., Levijoki, J., Izu, L. T., Boyd, W. D., Bányász, T., Bers, D. M., & Chen-Izu, Y. (2018). Complex electrophysiological remodeling in postinfarction ischemic heart failure. *Proceedings of the National Academy of Sciences*, *115*(13), E3036–E3044. https://doi.org/10.1073/pnas.1718211115

Hoch, B., Meyer, R., Hetzer, R., Krause, E.-G., & Karczewski, P. (1999). Identification and Expression of δ-Isoforms of the Multifunctional Ca^{2+}/Calmodulin-Dependent Protein Kinase in Failing and Nonfailing Human Myocardium. *Circulation Research*, *84*(6), 713–721. https://doi.org/10.1161/01.RES.84.6.713

Høydal, M. A., Kirkeby-Garstad, I., Karevold, A., Wiseth, R., Haaverstad, R., Wahba, A., Stølen, T. L., Contu, R., Condorelli, G., Ellingsen, Ø., Smith, G. L., Kemi, O. J., & Wisløff, U. (2018). Human cardiomyocyte calcium handling and transverse tubules in mid-stage of post-myocardial-infarction heart failure. *ESC Heart Failure*, *5*(3), 332–342. https://doi.org/10.1002/ehf2.12271

Jiang, M. T., Lokuta, A. J., Farrell, E. F., Wolff, M. R., Haworth, R. A., & Valdivia, H. H. (2002). Abnormal Ca^{2+} Release, but Normal Ryanodine Receptors, in Canine and Human Heart Failure. *Circulation Research*, *91*(11), 1015–1022. https://doi.org/10.1161/01.RES.0000043663.08689.05

Maier, L. S., Zhang, T., Chen, L., DeSantiago, J., Brown, J. H., & Bers, D. M. (2003). Transgenic CaMKIIδ_C Overexpression Uniquely Alters Cardiac Myocyte Ca^{2+} Handling. *Circulation Research*, *92*(8), 904–911. https://doi.org/10.1161/01.RES.0000069685.20258.F1

Marchiano, S., Nakamura, K., Reinecke, H., Neidig, L., Lai, M., Kadota, S., Perbellini, F., Yang, X., Klaiman, J. M., Blakely, L. P., Karbassi, E., Fields, P. A., Fenix, A. M., Beussman, K. M., Jayabalu, A., Kalucki, F. A., Potter, J. C., Futakuchi-Tsuchida, A., Weber, G. J., … Murry, C. E. (2023). Gene editing to prevent ventricular arrhythmias associated with cardiomyocyte cell therapy. *Cell Stem Cell*, *30*(4), 396-414.e9. https://doi.org/10.1016/j.stem.2023.03.010

Mincholé, A., Zacur, E., Ariga, R., Grau, V., & Rodriguez, B. (2019). MRI-Based Computational Torso/Biventricular Multiscale Models to Investigate the Impact of Anatomical Variability on the ECG QRS Complex. *Frontiers in Physiology*, *10*. https://doi.org/10.3389/fphys.2019.01103

Niederer, S. A., Kerfoot, E., Benson, A. P., Bernabeu, M. O., Bernus, O., Bradley, C., Cherry, E. M., Clayton, R., Fenton, F. H., Garny, A., Heidenreich, E., Land, S., Maleckar, M., Pathmanathan, P., Plank, G., Rodríguez, J. F., Roy, I., Sachse, F. B., Seemann, G., … Smith, N. P. (2011). Verification of cardiac tissue electrophysiology simulators using an N-version benchmark. *Philosophical Transactions of the Royal Society A: Mathematical, Physical and Engineering Sciences*, *369*, 4331–4351. https://doi.org/10.1098/rsta.2011.0139

Okada, J., Washio, T., Maehara, A., Momomura, S., Sugiura, S., & Hisada, T. (2011). Transmural and apicobasal gradients in repolarization contribute to T-wave genesis in human surface ECG. *American Journal of Physiology-Heart and Circulatory Physiology*, *301*(1), H200–H208. https://doi.org/10.1152/ajpheart.01241.2010

Paci, M., Passini, E., Klimas, A., Severi, S., Hyttinen, J., Rodriguez, B., & Entcheva, E. (2020). All-Optical Electrophysiology Refines Populations of In Silico Human iPSC-CMs for Drug Evaluation. *Biophysical Journal*, *118*(10), 2596–2611. https://doi.org/10.1016/j.bpj.2020.03.018

Poch, C. M., Foo, K. S., De Angelis, M. T., Jennbacken, K., Santamaria, G., Bähr, A., Wang, Q.-D., Reiter, F., Hornaschewitz, N., Zawada, D., Bozoglu, T., My, I., Meier, A., Dorn, T., Hege, S., Lehtinen, M. L., Tsoi, Y. L., Hovdal, D., Hyllner, J., … Laugwitz, K.-L. (2022). Migratory and anti-fibrotic programmes define the regenerative potential of human cardiac progenitors. *Nature Cell Biology*, *24*, 659–671. https://doi.org/10.1038/s41556-022-00899-8

Ringenberg, J., Deo, M., Filgueiras-Rama, D., Pizarro, G., Ibañez, B., Peinado, R., Merino, J. L., Berenfeld, O., & Devabhaktuni, V. (2014). Effects of Fibrosis Morphology on Reentrant Ventricular Tachycardia Inducibility and Simulation Fidelity in Patient-Derived Models. *Clinical Medicine Insights: Cardiology*, *8s1*. https://doi.org/10.4137/CMC.S15712

Romero, L., Pueyo, E., Fink, M., & Rodríguez, B. (2009). Impact of ionic current variability on human ventricular cellular electrophysiology. *American Journal of Physiology-Heart and Circulatory Physiology*, *297*(4), H1436–H1445. https://doi.org/10.1152/ajpheart.00263.2009

Sachetto Oliveira, R., Martins Rocha, B., Burgarelli, D., Meira, W., Constantinides, C., & Weber dos Santos, R. (2018). Performance evaluation of GPU parallelization, space-time adaptive algorithms, and their combination for simulating cardiac electrophysiology. *International Journal for Numerical Methods in Biomedical Engineering*, *34*(2), e2913. https://doi.org/10.1002/cnm.2913

Tomek, J., Bueno-Orovio, A., Passini, E., Zhou, X., Minchole, A., Britton, O., Bartolucci, C., Severi, S., Shrier, A., Virag, L., Varro, A., & Rodriguez, B. (2019). Development, calibration, and validation of a novel human ventricular myocyte model in health, disease, and drug block. *eLife*, *8*, e48890. https://doi.org/10.7554/eLife.48890

Tomek, J., Bueno-Orovio, A., & Rodriguez, B. (2020). ToR-ORd-dynCl: an update of the ToR-ORd model of human ventricular cardiomyocyte with dynamic intracellular chloride. *BioRxiv [Preprint]*. https://doi.org/10.1101/2020.06.01.127043

Valdivia, C. R., Chu, W. W., Pu, J., Foell, J. D., Haworth, R. A., Wolff, M. R., Kamp, T. J., & Makielski, J. C. (2005). Increased late sodium current in myocytes from a canine heart failure model and from failing human heart. *Journal of Molecular and Cellular Cardiology*, *38*(3), 475–483. https://doi.org/10.1016/j.yjmcc.2004.12.012

Zhou, X., Wang, Z. J., Camps, J., Tomek, J., Santiago, A., Quintanas, A., Vazquez, M., Vaseghi, M., & Rodriguez, B. (2024). Clinical phenotypes in acute and chronic infarction explained through human ventricular electromechanical modelling and simulations. *eLife*, *13*, RP93002. https://doi.org/https://doi.org/10.7554/eLife.93002.3
