# Supplementary material for: In silico optimization of regenerative cell therapy in the infarcted human ventricles to mitigate arrhythmic burden
Source: Stem Cell Reports. 2026 Jul 9;21(8):103007. doi: 10.1016/j.stemcr.2026.103007 (PMC13476901; doi:10.1016/j.stemcr.2026.103007)
Supplement: Supplementary file 2 [file mmc6.pdf]

STEM CELL REPORTS
Article
OPEN ACCESS

In silico optimization of regenerative cell therapy in the infarcted human ventricles to mitigate arrhythmic burden

Leto Luana Riebel,[1,3,*] Zhinuo Jenny Wang,[1] Xin Zhou,[1] Lucas Arantes Berg,[1] Cristian Trovato,[2] and Blanca Rodriguez[1,*]
[1]Department of Computer Science, British Heart Foundation Centre of Research Excellence, University of Oxford, Oxford, UK
[2]Systems Medicine, Clinical Pharmacology & Safety Science, R&D, AstraZeneca, Cambridge, UK
[3]Lead contact
*Correspondence: leto.riebel@cs.ox.ac.uk (L.L.R.), blanca.rodriguez@cs.ox.ac.uk (B.R.)
https://doi.org/10.1016/j.stemcr.2026.103007

SUMMARY

Myocardial infarction remains a frequent cause of heart failure and mortality. Cell therapy has shown promise in regenerating the damaged tissue, but delivered cells may beat spontaneously and produce ventricular arrhythmias, hindering clinical application. Here, we conducted multiscale computer simulations of the electrical activity of the infarcted human ventricles including the cardiac conduction system to identify and mitigate pro-arrhythmic mechanisms following cell delivery. Firstly, our simulations show how arrhythmic risk increases from before to after cell injection and further during the first two weeks post-delivery. Secondly, we suggest that concurrently targeting the funny current, the inward rectifier potassium current, the sodium-potassium pump current, and the rapid delayed outward rectifier potassium current may reduce automaticity and re-entry while maximizing calcium transient amplitude and thus contractility. Our study demonstrates how modeling and simulation enables the design of anti-arrhythmic strategies to improve therapy safety while preserving efficacy.

INTRODUCTION

Myocardial infarction (MI) and its long-term consequences, particularly heart failure, remain among the leading causes of death worldwide. Since adult human hearts do not sufficiently regenerate on their own, myocardial damage is largely irreversible. To ease the global burden of heart failure and improve cardiac function post-MI, delivery of stem cells has been explored (Jebran et al., 2025; Liu et al., 2018; Shiba et al., 2016). While pre-clinical studies have shown promising effects, ventricular arrhythmias, particularly during the first weeks after cell injection, have been observed and are suspected to arise from the immature cells' spontaneous beating (Chong et al., 2014; Liu et al., 2018; Marchiano et al., 2023; Romagnuolo et al., 2019).

Previous studies have identified upregulation of the inward rectifier potassium current (I_{K1}) and suppression of the funny current (I_f) as crucial anti-automaticity targets (Goversen et al., 2018; Kim et al., 2015; Verkerk and Wilders, 2023). One recent study found a combination of these together with a block of the T-type calcium current and the sodium-calcium exchanger current (I_{NaCa}) necessary to suppress spontaneous beating (Marchiano et al., 2023), highlighting a complex mechanism behind the cells' automaticity. While these strategies offer a vital way to reduce arrhythmias, it is unclear how they might impact the cells' calcium dynamics and, consequently, their efficacy. Particularly the sodium-calcium exchanger is crucial for regulating intracellular calcium levels throughout the action potential and may hence affect physiological calcium-induced calcium release and contractility (Henderson et al., 2004; Neco et al., 2010). In addition to automaticity-induced arrhythmias, previous human-based *in silico* studies have highlighted a variety of re-entry pathways caused by the cells' immature tissue conductivity and action potential morphology (Fassina et al., 2023; Riebel et al., 2024; Yu et al., 2022); however, due to limited clinical trial data, these findings remain to be corroborated. To enable safe clinical application of cell therapy post-MI, effective anti-automaticity as well as anti-re-entry targets need to be identified.

The development and testing of new treatments, whether they reach the clinic or not, is expensive (Sertkaya et al., 2024). Multiscale human modeling and simulation offers a powerful technology to augment the drug discovery and development process by identifying safety risks early on (Passini et al., 2021; Viceconti et al., 2021). Furthermore, simulation studies present an efficient tool to design anti-arrhythmic strategies that improve therapy success (Dasí et al., 2024; Rosales et al., 2024).

In this study, we harness the powers of human-based modeling and simulation to identify and optimize ionic targets that reduce both automaticity-induced and re-entrant arrhythmias after cell delivery, as a post-MI therapy. For this, we conduct computer simulations from the action potential to the electrical excitation and relaxation of the ventricles to evaluate pro-arrhythmic mechanisms following cell delivery in the infarcted human ventricles including the Purkinje conduction system (Riebel et al., 2024). To computationally model electrical maturation of

Published by Elsevier Inc. on behalf of International Society for Stem Cell Research.
This is an open access article under the CC BY license (http://creativecommons.org/licenses/by/4.0/).

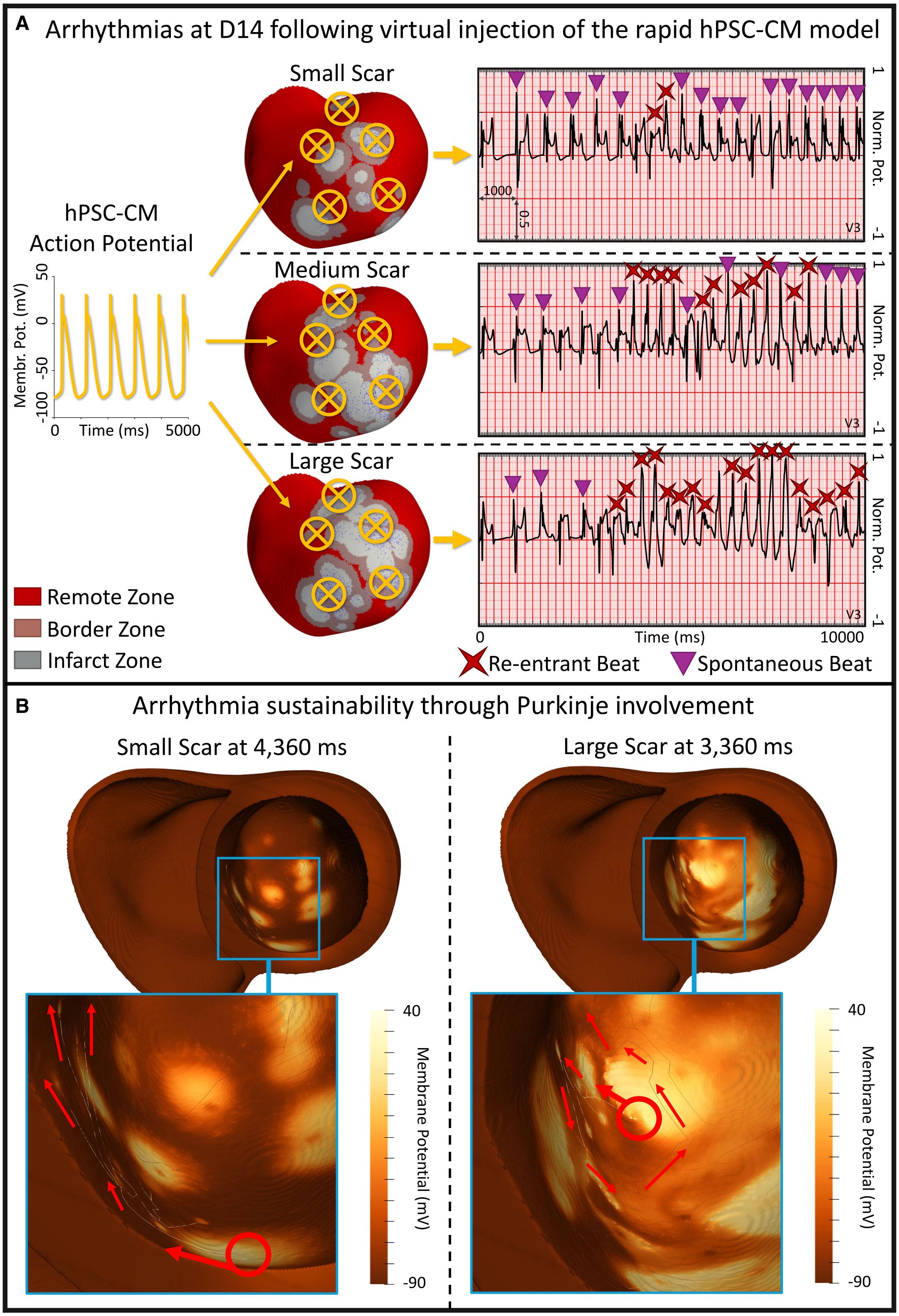

(legend on next page)

injected cells from day 0 (D0) to day 14 (D14), we consulted experimentally reported ion channel gene expression data of *in vivo* maturing injected cells from Marchiano et al. (2023). We then set out to determine: (1) the risk of automaticity-induced arrhythmias at D0 and D14 after cell injection, also considering variability in scar size, (2) the risk of re-entry before and at D0 and D14 after delivery, (3) novel ionic targets that supress spontaneous beats while maintaining physiological calcium transients, and (4) a combination of ionic targets to optimize therapy safety by reducing automaticity-induced as well as re-entrant arrhythmias.

RESULTS

Establishing credibility: Computer simulations of automaticity-induced arrhythmias two weeks after cell delivery

As expected from experimental observations (Chong et al., 2014; Marchiano et al., 2023; Romagnuolo et al., 2019) and in line with our previous study (Riebel et al., 2024), human stem cell-derived cardiomyocytes (hPSC-CMs) immediately after delivery (D0) did not produce spontaneous depolarizations in the ventricles (see Figure S1A). To investigate variability in hPSC-CM electrophysiology and corresponding automaticity, we created a rapid hPSC-CM model by upregulating the sodium-calcium exchanger, the funny current, and the sarco-endoplasmic reticulum calcium ATPase (SERCA) in our baseline hPSC-CM model (see STAR Methods for further detail). While at D0 this rapid hPSC-CM model caused spontaneous beats at 46 beats per minute (bpm) in the ventricles, it was overridden by 1 Hz sinus pacing (see the lack of automaticity in Figure S1C).

At D14 post-delivery, the baseline hPSC-CM model caused ectopy in the ventricles with 42 bpm but, again, was suppressed by sinus pacing (see Figure S1B). However, as shown in Figure 1A, the rapid hPSC-CM model, which beat spontaneously with a frequency of 66 bpm at D14 in isolation, produced spontaneous ventricular depolarizations in all three investigated scars. This spontaneous beating increased over time and facilitated ventricular tachycardia-like arrhythmias with intermittent re-entrant wavefronts in all three scars. As highlighted in the annotated ECGs of Figure 1A, the proportion of re-entrant wavefronts compared to spontaneous depolarizations decreased with decreasing scar size (from 82% in the large scar over 52% in the medium scar to 12% in the small scar). While re-entry dominated over spontaneous activity in the large scar, automaticity appeared to reach a steady-state toward the end of the 10 s simulations in the medium and small scar (see Video S1). Spontaneous depolarizations occurred up to every 420 ms in the ventricles corresponding to a tachycardia frequency of approximately 140 bpm, similar to pre-clinical observations (Marchiano et al., 2023). hPSC-CM automaticity increased in the ventricles compared to isolation (140 versus 66 bpm); as we showed previously (Riebel et al., 2024), hPSC-CM automaticity in the ventricles is favored by depolarized chronic infarcts and may further increase under fast heartrates, which here were facilitated through re-entrant rotors (see ECGs and membrane potentials in Figure 1).

Quantifying re-entry risk: Arrhythmic burden increases from before to after virtual cell delivery and from day 0 to day 14

We next set out to quantify the re-entry risk after cell delivery by applying ectopic stimuli in the host border zone (S1-S2 stimulus protocol, further described in the STAR Methods). As shown in Figure 2, we observed few re-entries before virtual injection of the baseline hPSC-CM model. In the small scar, which contained spread out scar islands, no re-entries were observed. In the medium and large scar each, two non-sustained and one sustained re-entry occurred. All re-entries in the large scar and two out of three in the medium scar were induced by ectopic stimulus locations close to the apex and followed a regular pattern. As the scar cores were unexcitable and only slowly conducting, the wavefront traveled through isthmuses between the dense scar cores and re-entered at the site of the applied ectopic stimulus. The third re-entry in the medium scar was facilitated through a Purkinje-myocyte junction on the anterior left ventricular wall on the edge between infarct and border zone.

As shown in Figure 3, the vulnerable windows (i.e., the range of time intervals between sinus rhythm and applied ectopic stimulus during which re-entry was inducible),

Figure 1. Automaticity-induced arrhythmia burden at D14 after virtual injection of rapidly beating hPSC-CMs in a human biventricular model with Purkinje and three different anteroseptal MI scars

(A) Left: action potential traces of the rapid D14 hPSC-CMs beating spontaneously in virtual isolation. Middle: five out of six virtual hPSC-CM injection locations highlighted in the biventricular mesh (remaining one is located in the septum). Right: ECG lead V3 traces of arrhythmias after virtual cell injection during 1 Hz stimulation at the bundle of His.

(B) Snapshots from Video S1 corresponding to the ECGs in A highlighting the involvement of the Purkinje system in sustaining the arrhythmias. The red circles identify the Purkinje-myocyte junctions through which the wavefront re-entered; red arrows indicate the subsequent path of the wavefront through the Purkinje conduction network. Also see Video S1 and Figure S1.

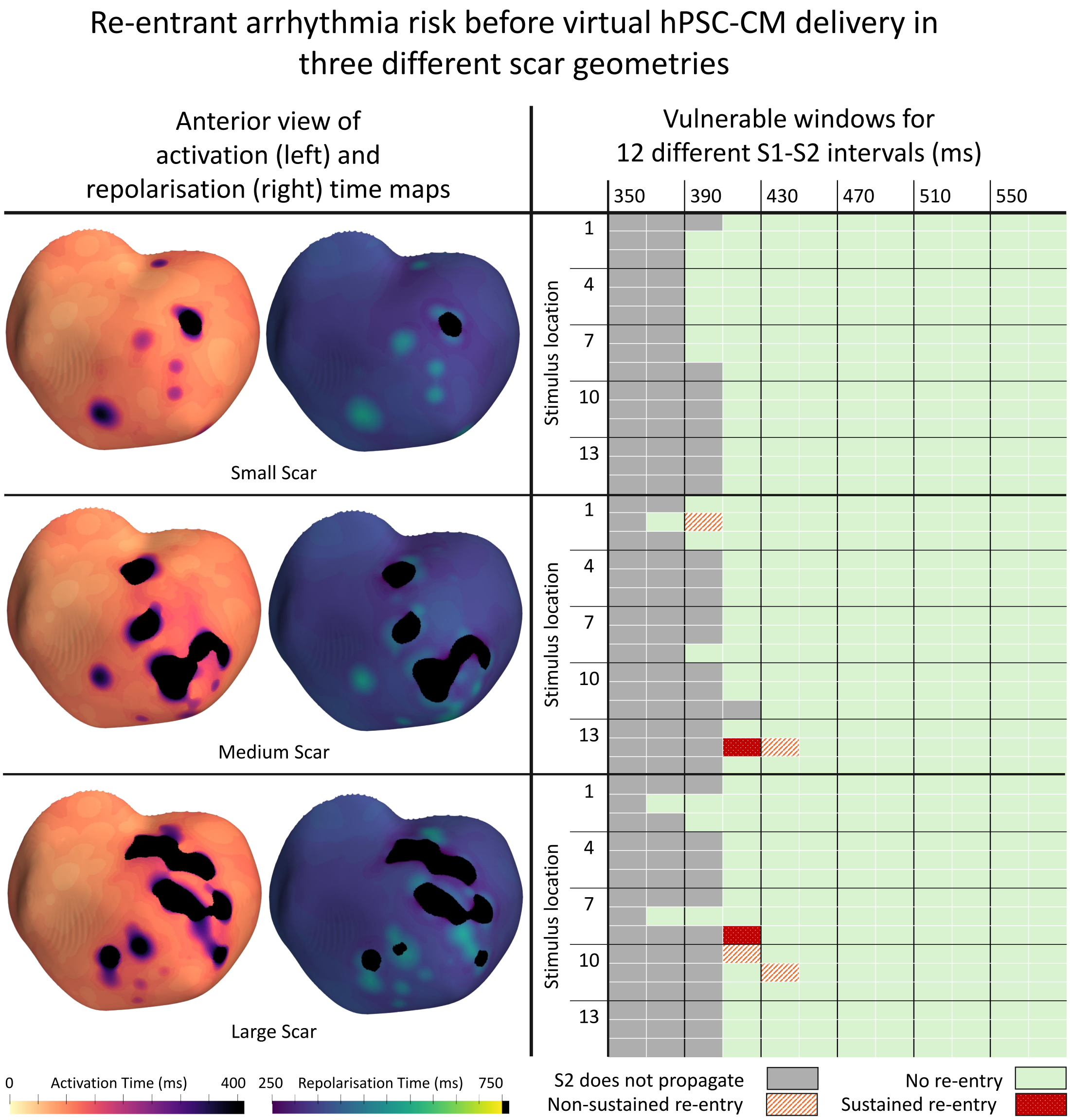

Figure 2. Re-entry risk in a human-based biventricular model with three scars before virtual delivery of baseline hPSC-CMs
Left: activation and repolarization time maps. Right: vulnerable windows for different S1-S2 intervals and S2 stimulus locations. Note, ectopic stimulus locations differ in each scar and were ordered from most anterior-basal to most inferior-apical.

increased from before to after virtual cell delivery in all three investigated scars: from 0 to 640 ms in the small scar, from 60 to 100 ms in the medium scar, and from 60 to 760 ms in the large scar (compare Figures 2 and 3). While re-entry risk rose in all three scars, it did not linearly increase with larger scar size—i.e., re-entry risk at D0 was highest in the large scar but lowest in the medium scar. Re-entry sustainability, however, was substantially lower in the small scar (3%, i.e., 1 out of 32 re-entries after cell delivery) than in the medium (33% before and 80% after delivery) and large scar (33% before and 76% after delivery). We observed that re-entries in the small scar relied on Purkinje involvement with two Purkinje-myocyte junctions isolated in the center of the scar cores emerging as particularly pro-arrhythmic (see Video S2). In the medium and large scar, the infarct cores were larger and instead, re-entries relied on a mix of Purkinje and tissue pathways. Thus, the large scar provided several routes for conduction block and re-entry, with no single pro-arrhythmic area emerging. In the medium scar, the tissue pathways for re-entry were more limited and 80% of re-entries involved the same large apical scar core (visible as not fully repolarizing in the repolarization map of Figure 3). As highlighted when comparing the activation time maps in Figures 2

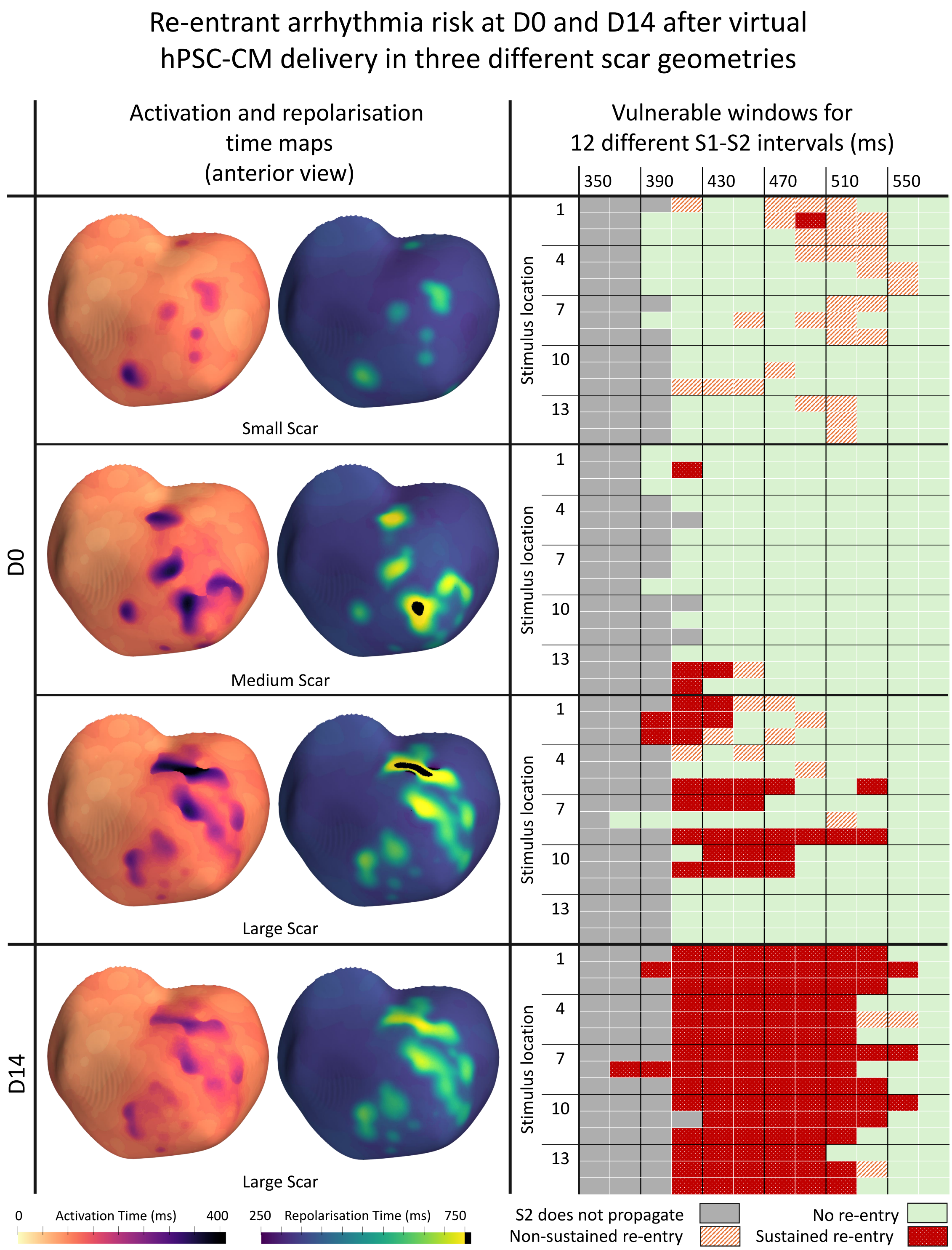

Figure 3. Re-entry risk in a human-based biventricular model with three different scars at D0 and D14 after virtual delivery of the baseline hPSC-CM model

Left: activation and repolarization time maps. Right: vulnerable windows for different S1-S2 intervals and S2 stimulus locations. Note, ectopic stimulus locations differ in each scar and were ordered from most anterior-basal to most inferior-apical. Also see Videos S2 and S3.

and 3, hPSC-CMs improved conductivity and excitability of the infarct and allowed for wavefronts to travel through scar cores, which before cell delivery were inaccessible (see Video S3).

Next, we investigated the re-entry risk at D14 post-delivery in the large scar (as this was the most pro-arrhythmic scenario at D0); the vulnerable window increased from 760 to 2,080 ms, as shown in the bottom of Figure 3. 97% (101 out of 104) of those D14 re-entries sustained for at least two re-entry cycles. As highlighted by the repolarization time maps in Figure 3, hPSC-CMs further improved conductivity within the infarct from D0 to D14. This was due to an increase of graft conduction velocity from 10 cm/s at D0 to 14.5 cm/s at D14—as conductivities were maintained, this was caused by maturation of ionic currents, particularly a 2.5-fold increase in the fast sodium current's (I_{NaF}) conductance (see STAR Methods section). The improved excitability within the infarct enabled new paths for the re-entrant wavefront; slow transmural conduction pathways emerged as particularly pro-arrhythmic at D14.

In summary, while virtually delivered cells improved conduction through the infarct and reduced activation time gradients across the ventricles, they also enabled new re-entry pathways transmurally and across the scar, particularly at D14 after delivery. This pro-arrhythmic substrate was due to the virtually injected cells having a longer action potential duration than the host cardiomyocytes, thus promoting heterogeneous ventricular repolarization, refractoriness, and pro-arrhythmic conduction block.

Virtual target discovery: Regulating the sodium-potassium pump to suppress automaticity-induced arrhythmias

To identify ionic targets that suppress hPSC-CM automaticity, we carried out a cellular sensitivity analysis by scaling ion channel conductances and recording their effects on the cells' spontaneous beating rate in virtual isolation. Our results, shown in Figure S2, identified the spontaneous beating frequency to be most sensitive to the sodium-potassium pump, with both a 45% increase as well as a 30% decrease in its maximum value (model parameter p_{NaK}) abolishing automaticity at D0. As shown in Figure 4A, reducing the maximum pump current caused intracellular sodium accumulation leading to an increase in the reverse mode of the sodium-calcium exchanger (I_{NaCa}, first spike in Figure 4A, right panel) and, ultimately, intracellular calcium accumulation and action potential duration shortening, as discussed in other studies (Bueno-Orovio et al., 2014; Faber and Rudy, 2000). Counterintuitively, and as depicted in Figure S3A, intracellular sodium accumulation over time resulted in an ultimately increased pump current, even though its initial maximum value was reduced. Further investigation in our model also showed an increase in the sarcoplasmic reticulum's calcium concentration. In the absence of applied pacing, this resulted in an impaired recovery of the ryanodine receptor-sensitive release current's (I_{rel}) inactivation gate (model parameter RyRc) leading to a reduced calcium-induced calcium release through the ryanodine receptors and a lack of spontaneous activity. While 1 Hz pacing caused physiological action potentials at D0 (see Figure S3A), calcium overload occurred in the sarcoplasmic reticulum at D14, where only diminished calcium transients and short action potentials could be produced (see Figure S4A). As such action potential heterogeneity may be pro-arrhythmic, we discarded lowering the maximum sodium-potassium pump current and considered its upregulation further.

Upregulation of the maximum sodium-potassium pump current suppressed spontaneous beats through the opposite effect, i.e., intracellular sodium and the reverse mode of the sodium-calcium exchanger were reduced (I_{NaCa}, first spike in Figure 4B, right panel) therefore leading to lower intracellular and sarcoplasmic reticulum calcium concentrations impairing the calcium clock (see Figures 4B and S3B). While increasing the maximum pump current produced physiological (but longer) action potentials in virtual isolation, the calcium transient amplitude was reduced by approximately 55% (see Figure S3B). At D14, increased maximum pump current on its own was not sufficient to abolish automaticity; hence, we combined it with a full block of the funny current and upregulation of the inward rectifier potassium current, as suggested also in other studies (Goversen et al., 2018; Marchiano et al., 2023). For a 50% increase in the conductance of the inward rectifier potassium current, a 40% and 65% increase in maximum sodium-potassium pump current was enough to stop spontaneous beating in the baseline and rapid hPSC-CM model, respectively. Pacing produced physiological action potentials and calcium transients in virtual isolation at D0 and D14, as shown in Figures S5 and S4B, respectively.

We further compared our suggested upregulation of maximum sodium-potassium pump current with the state-of-the-art approach of knocking out the sodium-calcium exchanger to inhibit stem cell automaticity (Marchiano et al., 2023). As depicted in Figure 4C, this comparison showed that (together with an increased inward rectifier potassium current and blocked funny current) fully blocking the exchanger caused intracellular calcium accumulation and small calcium transient amplitudes. We identified the main mechanism behind this as a substantially reduced ryanodine receptor-sensitive calcium release current, similar to when we reduced the maximum sodium-potassium pump current. Knock-out of the sodium-calcium exchanger, which is the main mechanism for calcium extrusion, caused accumulation of intracellular but also sarcoplasmic reticulum calcium. This led to insufficient relaxation of the ryanodine

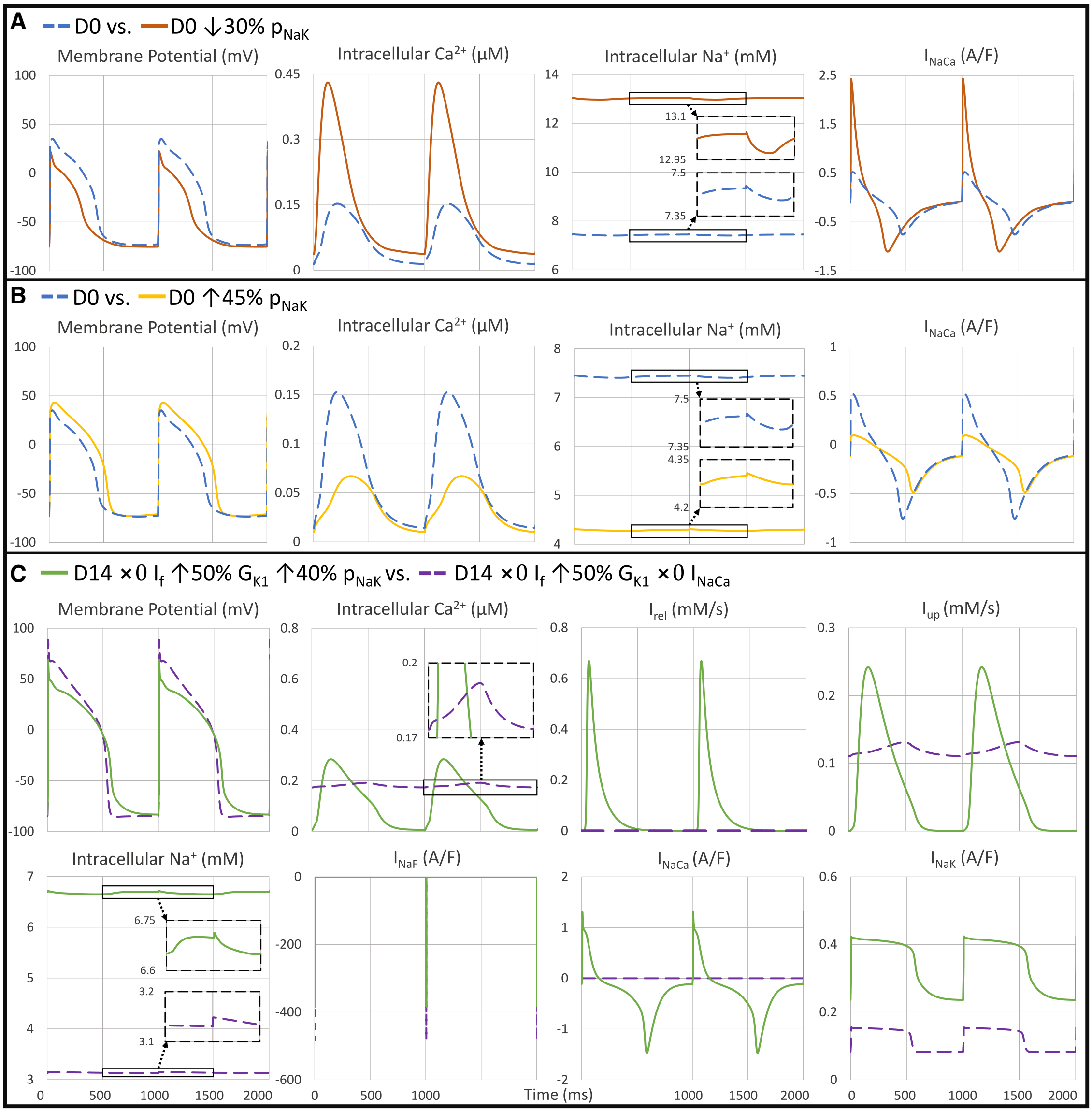

Figure 4. Action potential and ionic current traces of different hPSC-CM models virtually beating in isolation under 1 Hz pacing; beat 1,000 shown

(A) hPSC-CMs at D0 with and without downregulating the maximum sodium-potassium pump current (p_{NaK}).

(B) hPSC-CMs at D0 with and without upregulating p_{NaK}.

(C) hPSC-CMs at D14 with full funny current (I_f) block, upregulation of potassium currents' conductances (G_{K1} and G_{Kr}) and either full block of the sodium-calcium exchanger or p_{NaK} upregulation. Also shown are intracellular calcium (Ca^{2+}) and sodium (Na^{+}), the ryanodine receptor-sensitive release current (I_{rel}), the sarco-endoplasmic reticulum calcium ATPase (SERCA) current (I_{up}), and the fast sodium current (I_{NaF}). Also see Figures S3–S5.

receptors' activation and inactivation gates (RyRo and RyRc model parameters). In comparison, even though the calcium transient amplitude was reduced compared to the baseline hPSC-CM model with no ionic current modifications, upregulating the maximum sodium-potassium pump current produced more physiological calcium transients at both D0 and D14 (see Figures S5 and 4C, respectively).

Virtual target optimization: Regulating multiple potassium currents to reduce automaticity-induced as well as re-entrant arrhythmias

Next, we investigated how to combine the identified anti-automaticity targets, analyzed above, with anti-re-entry targets. Our cellular hPSC-CM sensitivity analysis, shown in Figure S2, as well as previous studies (Fassina et al.,

2023; Riebel et al., 2021), highlighted the rapid delayed outward rectifier potassium current (I_{Kr}) as a promising target to modulate the longer hPSC-CM action potential toward adult cardiomyocyte values. Hence, we created an automated algorithm aiming to determine scaling factors for three potassium currents (inward rectifier potassium, rapid delayed outward rectifier potassium, and sodium-potassium pump current), in addition to full funny current block. As described in the STAR Methods, this algorithm aimed to optimize calcium transient amplitude as well as action potential duration by maximizing an optimization index that considers both. First, we used a target hPSC-CM action potential duration at 90% repolarization of 330 ms (approximately ¾ of the action potential at D0). Achieving the closest match to this target (while optimizing calcium transient amplitude), our algorithm suggested scaling the maximum sodium-potassium pump current and the conductances of the inward and rapid delayed outward rectifier potassium currents by 1.75, 1.5, and 3.0, respectively (see Figure S6). At D0, this ionic current scaling combination decreased hPSC-CM action potential duration at 90% repolarization to 284 ms in virtual isolation (see Figure 5A) and increased graft conduction velocity in tissue from 10 cm/s to 12.5 cm/s. Higher conduction improved activation of the scar (see repolarization time maps in Figures 3 and 5A) and, similarly to the unoptimized hPSC-CM model at D14, enabled new re-entry pathways. Accordingly, in our biventricular simulations, this set of ionic scalings reduced the vulnerable window only marginally from 760 to 680 ms at D0 (see Figure 6A).

As ventricular repolarization was not sufficiently homogenized, we reduced the target hPSC-CM action potential duration in our algorithm by approximately half (see Figure S7). As shown in Figures 6B and 6C, after scaling the maximum sodium-potassium pump current and potassium currents' conductances (both inward and rapid delayed outward rectifier) by 1.75, 1.25, and 5 (respectively, and in addition to full funny current block), vulnerable windows decreased from 760 to 340 ms at D0 and from 2,080 to 100 ms at D14. Decreased arrhythmogenicity was the result of an increased graft conduction and reduced hPSC-CM action potential duration, which homogenized both activation and repolarization gradients, respectively (see Figures 5B and 5C and Video S4). Optimization increased graft conduction velocity by 25% and 24% at D0 and D14, respectively. Since conductivities were kept constant, this was caused by regulating these four potassium currents, all of which we previously showed to individually improve action potential upstroke velocity in the computational hPSC-CMs (Riebel et al., 2021). As shown in the calcium transient traces in Figures 5B and 5C, while our algorithm aimed to maximize the calcium transient amplitude, it was still substantially lower than before optimization.

DISCUSSION

In this study we present a novel *in silico* multiscale human electrophysiological framework to optimize new treatments, such as regenerative cell therapy, through biophysically detailed computer simulations. First, in a human biventricular model with three different post-MI scars, validated against experimental and clinical data, we showed that while cells remained quiescent immediately after delivery, more rapidly beating cells produced automaticity two weeks later, causing monomorphic ectopy-driven ventricular tachycardia in the small and medium scar, and polymorphic re-entry driven tachycardia in the large scar. Next, in the absence of spontaneous beats, we quantified the re-entry risk, which increased from before to after cell delivery and further from day 0 to day 14. Finally, we demonstrated how our framework enables identifying and optimizing anti-arrhythmic ionic target combinations—blocking the funny current and upscaling the maximum sodium-potassium pump current as well as the inward and rapid delayed outward rectifier potassium currents emerged as a novel strategy to suppress spontaneous beating and reduce re-entrant arrhythmia susceptibility while maximizing calcium transient amplitude.

Our *in silico* framework of MI, including also the Purkinje conduction network, was constructed, calibrated, and evaluated using human-based experimental and clinical evidence and incorporates extensive biophysical knowledge gained throughout the development of modeling and simulation technologies (Camps et al., 2024; Mincholé et al., 2019; Tomek et al., 2019; Zhou et al., 2024). As described in the STAR Methods section, credibility of our human-based simulation framework is provided by addressing verification, validation, and uncertainty quantification principles (Musuamba et al., 2021; Viceconti et al., 2021) in the present and previous studies, specifically in Riebel et al. (2024).

In our simulations, fast-beating virtual hPSC-CMs produced spontaneous depolarizations in the ventricles two weeks after delivery and facilitated ventricular tachycardia-like arrhythmias with a frequency of up to 140 bpm, as observed experimentally (Marchiano et al., 2023). While, hPSC-CM automaticity has been identified as the main driver of ventricular tachycardia in non-human primate studies (Liu et al., 2018), it has been noted that arrhythmias after cell delivery are rarely observed in small animal models and that heartrate and size may be a crucial factor (Chong et al., 2014). In our study, hPSC-CM automaticity initiated ventricular tachycardia but wave breakup and re-entry rotors were involved in sustaining it. In our small scar, automaticity prevailed as the dominant arrhythmia mechanism, highlighted by largely monomorphic tachycardia patterns in the ECG. With increasing scar

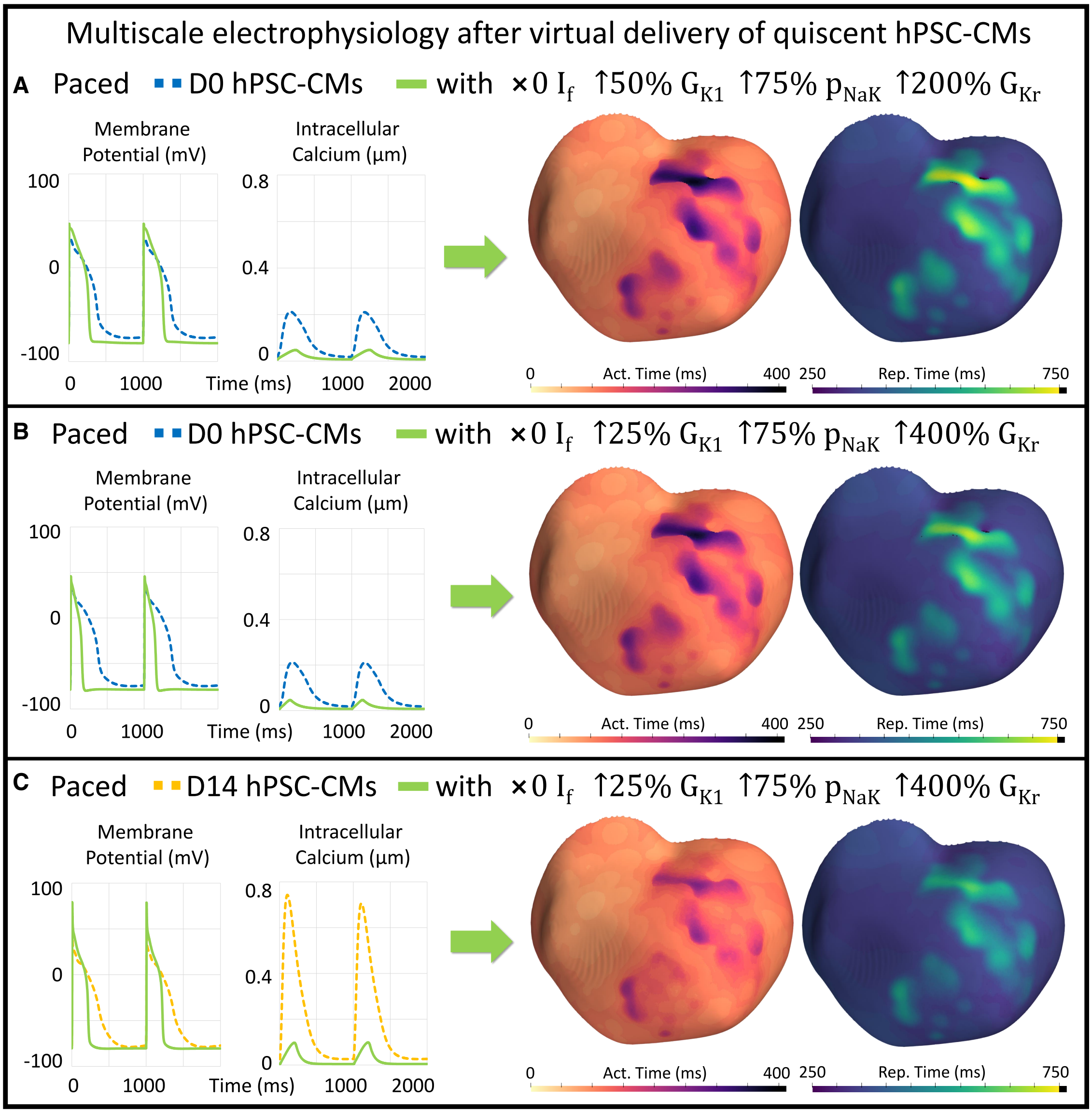

Figure 5. Left: action potentials and calcium transients of the baseline hPSC-CM model before and after optimisation in virtual isolation under 1 Hz pacing. Right: activation and repolarisation time maps after virtual delivery of optimized hPSC-CMs (green traces from the left) into a human-based biventricular chronic post-MI model

Compare to activation and repolarization time maps before optimization in Figure 3. (A and B) Optimised hPSC-CM model at D0. (C) Optimised hPSC-CM model at D14. Note, the optimized hPSC-CMs (green traces on the left) did not beat spontaneously. I_f: funny current; G_{K1}: conductance of the inward rectifier potassium current; p_{NaK}: maximum sodium-potassium pump current; G_{Kr}: conductance of the rapid delayed outward rectifier potassium current.

size, wave breakup into re-entry rotors increased; in our large scar, the ECG was more chaotic, highlighting the absence of a single ectopic tachycardia source. Overall, our human-based model with clinically reported infarct masses suggests that while automaticity may indeed produce regular ventricular tachycardia-like patterns in small scars, spontaneous depolarizations may break into re-entrant rotors in large scars and facilitate more chaotic arrhythmia patterns—which may not surface in animal models with smaller scars. Delivery modality may also affect arrhythmic risk; recent experiments of cell sheet transplantation, rather than cell injection, showed no arrhythmias in non-human primates (Jebran et al., 2025). Additionally, the Purkinje conduction system has been shown crucial in ventricular tachycardia sustainability in human (Bayer et al., 2025; Haissaguerre et al., 2016) and

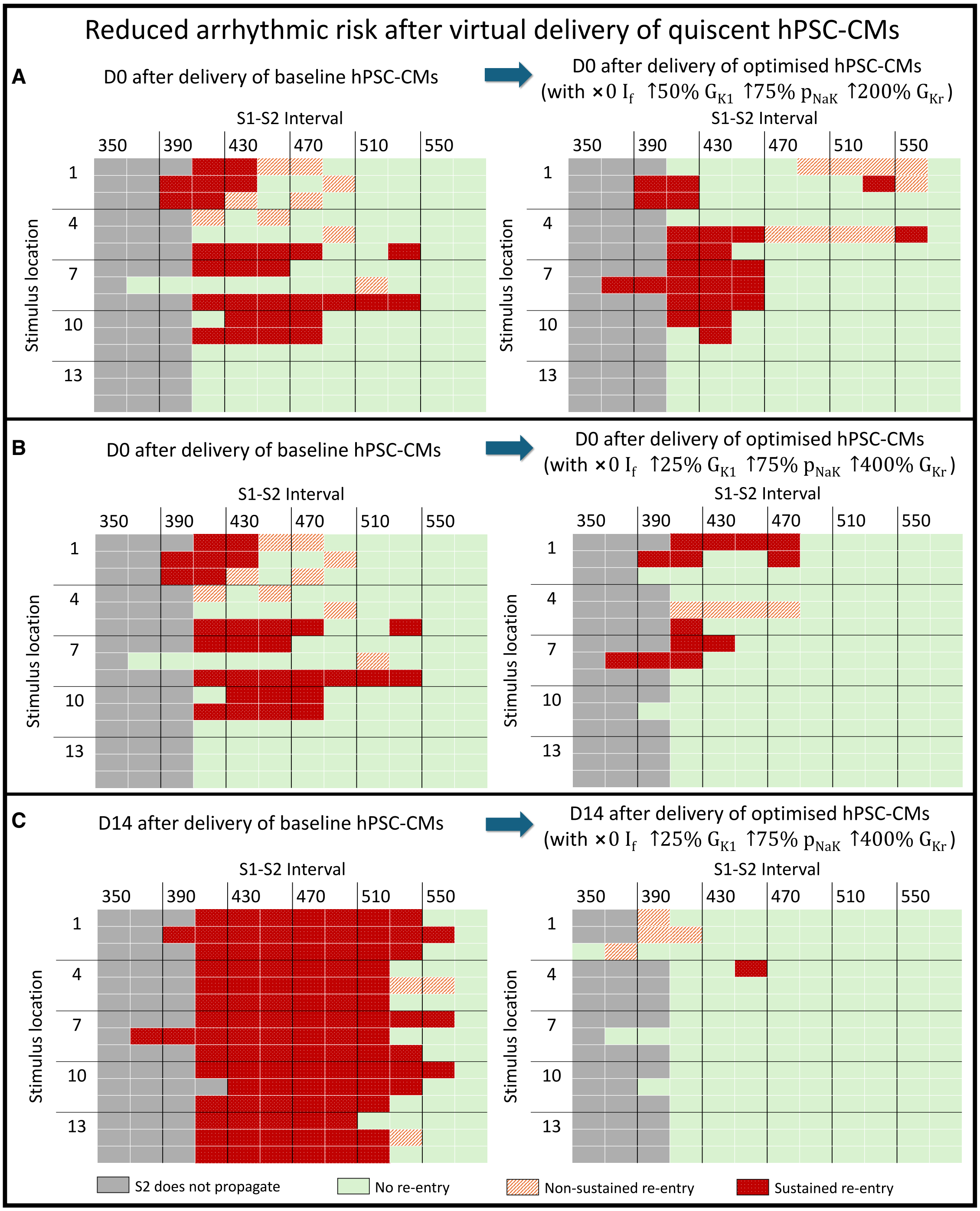

Figure 6. Comparison of re-entry risk after virtual delivery of baseline versus optimized hPSC-CMs into a human-based biventricular model with a large chronic infarct

(A and B) Vulnerable windows for different S1-S2 intervals and S2 stimulus locations at D0 after virtual cell delivery.

(C) Vulnerable windows for different S1-S2 intervals and S2 stimulus locations at D14 after virtual cell delivery. The ectopic stimulus for location 3 and S1-S2 interval of 330 ms (not shown in the right table) did not propagate. Note, ectopic stimulus locations were ordered from most anterior-basal to most inferior-apical. Also see Video S4. I_f: funny current; G_{K1}: conductance of the inward rectifier potassium channel; p_{NaK}: maximum sodium-potassium pump current; G_{Kr}: conductance of the rapid delayed outward rectifier potassium current.

also played a critical role in arrhythmia sustainability in our study.

Maturation of delivered cells was a crucial factor for arrhythmic risk in our study (which showed no spontaneous depolarizations at day 0 after virtual delivery) and has also been highlighted experimentally (Marchiano et al., 2023). In another *in silico* study, Paci et al. (2012) used experimental evidence to construct early and late maturation cells. Similar to our study, their matured model had increased inward rectifier and transient outward potassium, fast sodium, sodium-calcium exchanger, and L-type calcium currents but, contrarily, decreased funny and rapid delayed outward rectifier potassium currents (Paci et al., 2012). According to Marchiano et al. (2023), while the genes encoding the funny and rapid delayed outward rectifier potassium currents increase initially, possibly contributing to the surge of arrhythmias two weeks after injection, they ultimately decrease by day 84. It should be noted, that Marchiano et al. (2023) investigated gene expression of hPSC-CMs that had matured for 18–20 days *in vitro* before delivery. Furthermore, to estimate ion channel conductances in our hPSC-CM models from experimental hPSC-CM gene expression data (Marchiano et al., 2023), we consulted experimental insights relating gene expression to functional change in human cell line studies (Stevens and Brown, 2013). However, as other factors may impact the final ion channel function, future work may create D0 and D14 hPSC-CM populations or consult multi-omics data to assess this uncertainty. While we scaled the sarco-endoplasmic reticulum calcium ATPase, sodium-calcium exchanger, and funny currents by 2.5 to create rapidly beating D0 and D14 hPSC-CM models, Gibbs and Boyle (2026) simulated fast hPSC-CM automaticity by increasing the funny, rapid and slow delayed outward rectifier potassium, and T-type calcium current conductances 11.5-fold and by blocking the inward rectifier potassium current. Their fast hPSC-CM model beat every 250 ms and produced automaticity-induced tachycardia-like arrhythmias in human ventricular slice models (Gibbs and Boyle, 2026).

Electrophysiological maturation, particularly of the fast sodium current, also caused an increase in our hPSC-CM model's conduction velocity from 10 cm/s at D0 to 14.5 cm/s at D14. It should be noted that while experimental studies record similar D14 conduction velocities, they may show substantially slower conduction at earlier time points; however, they often investigate cell fibers without transplanting them (Hansen et al., 2018). In addition to electrophysiological maturation, maturing connectivity between the graft and host, as suggested experimentally (Dhahri et al., 2022), may further increase conduction. *In silico* studies have suggested a link between this evolving graft-to-host coupling and arrhythmic risk (Fassina et al., 2023; Gibbs et al., 2023; Rosales et al., 2024; Yu et al., 2022).

To suggest anti-arrhythmic strategies, we utilized the mechanistic nature of modeling and simulation with the sodium-potassium pump emerging as a novel target. Interestingly, experimental studies have suggested that the sodium-potassium pump current may increase as hPSC-CMs mature (Otsu et al., 2005), which could contribute to the reduction of automaticity after three weeks post injection and support our findings. The effects of regulating the sodium-potassium pump on spontaneous beating, although to a lesser extent, have also been highlighted in a sensitivity analysis by Paci et al. (2012). While our results indicate that for some hPSC-CM phenotypes targeting the sodium-potassium pump may be sufficient, modulating also the inward rectifier potassium and funny currents may be required in others. The latter two currents have long been known to impact the immaturity and automaticity of hPSC-CMs (Goversen et al., 2018; Marchiano et al., 2023; Verkerk and Wilders, 2023). While in our D0 (but not D14) model, funny current block and sufficient inward rectifier potassium current upregulation stopped automaticity, this may be insufficient in some cell types due to the calcium clock acting as a second depolarizing mechanism (Kim et al., 2015; Marchiano et al., 2023).

Next, we systematically identified ionic current combinations that minimize arrhythmic risk while allowing for physiological calcium transients. Current state-of-the-art agrees to block the funny current and upregulate the inward rectifier potassium current but, instead of targeting the sodium-potassium pump, further suggests knockout of the T-type calcium channel and the sodium-calcium exchanger (Marchiano et al., 2023). In our simulations, a full sodium-calcium exchanger block resulted in calcium accumulation and a reduced calcium transient amplitude, which, although to a lesser extent, has been suggested also in the supplemental material of Marchiano et al. (2023). However, as it is a main source of calcium extrusion (Ottolia et al., 2013), suppressing the sodium-calcium exchanger is generally associated with an increase in the calcium transient amplitude despite a decreased L-type calcium current (Ismaili et al., 2022; Ozdemir et al., 2008). Nevertheless, full sodium-calcium exchanger knock-out can facilitate intracellular and sarcoplasmic reticulum calcium accumulation and may ultimately hinder effective contractility (Henderson et al., 2004; Koivumäki et al., 2018; Figure 3; Koushik et al., 2001). Ultimately, the sodium-calcium exchanger and sodium-potassium pump depend on each other's regulation of sodium concentrations—modulating either will affect the other. Interestingly, amiodarone has been suggested as an anti-arrhythmic strategy in pre-clinical trials of cell delivery

(Nakamura et al., 2021) and is known to impact both, the sodium-calcium exchanger and the sodium-potassium pump (Gray et al., 1998; Watanabe and Kimura, 2000).

Finally, our simulations showed that ventricular arrhythmias may also be induced in the absence of spontaneous hPSC-CM depolarizations. Due to increasing conduction and action potential heterogeneity, re-entry risk increased from D0 to D14 after virtual cell delivery. To reduce this re-entry burden, in our previous suggested quiescent hPSC-CMs (blocking the funny current and upregulating inward rectifier potassium and maximum sodium-potassium pump currents), we further suggest an increase of the rapid delayed outward rectifier potassium current. Targeting this current to make hPSC-CM action potentials more adult-like has been put forward also in other *in silico* studies, which further identified the fast sodium channel as a potential target (Fassina et al., 2022, 2023). It should be noted that while we aimed to maximize the calcium transient amplitude, some reduction appeared necessary to avoid automaticity-induced arrythmias, linked to the cells' calcium clock (Kim et al., 2015). Hence, our virtual hPSC-CMs showed a reduced calcium transient after optimization. This was largely caused by a block in the funny current, which on its own reduced the calcium transient amplitude by approximately 40%. Furthermore, blocking the inward rectifier potassium channel has been linked to larger calcium transients and active tension (Telle et al., 2025)—hence, increasing this current may further reduce contractility. While our suggested optimized ionic target combination includes such an increase in the inward rectifier potassium current, our results show that spontaneous beating may also be inhibited by funny current block and sufficient rapid delayed outward rectifier potassium and maximum sodium-potassium pump current upregulation alone, but will likely be affected by the underlying hPSC-CM type.

Overall, our study proposes targeting several potassium currents (including the funny, inward rectifier potassium, rapid delayed outward rectifier potassium, and sodium-potassium pump currents) to produce quiescent hPSC-CMs, which are also less likely to facilitate re-entries. These ion channels may be regulated through gene editing before cell delivery, which has shown successful in modulating several ionic currents concurrently in hPSC-CMs (Marchiano et al., 2023). Ultimately, optimal scaling factors will vary between cell types, maturation protocols, and the patient, e.g., patients with a slower repolarization may require the hPSC-CMs' action potential to be shortened less. The computational pipeline we have presented here allows for fast and efficient optimization and testing of new treatments for a range of different cell lines, delivery modalities, and patient substrates.

RESOURCE AVAILABILITY

Lead contact

Requests for further information and resources should be directed to the lead contact, Leto Luana Riebel (leto.riebel@cs.ox.ac.uk).

Materials availability

This study did not generate new unique reagents.

Data and code availability

Customized code for the 3D model solver is available at https://github.com/LLRiebel/MonoAlg3D_C-2023. The Purkinje network, biventricular meshes before and after virtual cell delivery, and ECG electrode locations are as in our previous study Riebel et al. (2024) and can be found in https://doi.org/10.5281/zenodo.14699735. Requests for any additional information and resources should be directed to the lead contact, Leto Luana Riebel (leto.riebel@cs.ox.ac.uk).

ACKNOWLEDGMENTS

This project was funded by: a BBSRC PhD iCASE (BB/V509395/1) and Russell Studentship Agreement with AstraZeneca (R67719/CN001) for L.L.R. awarded to B.R., an Oxford-Bristol Myers Squibb Fellowship to X.Z. (R39207/CN063), a grant from the National Council for Scientific and Technological Development (CNPq) to L.A.B. (446127/2024-8), a Wellcome Trust Senior Research Fellowship in Basic Biomedical Sciences to B.R. (214290/Z/18/Z), an EPSRC Impact Acceleration Account Technology Fund (0016113), the Oxford BHF Center of Research Excellence (RE/24/130024), and the EPSRC project CompBioMed X (EP/X019446/1).

The Paci2020 model was provided by Dr. Michelangelo Paci and the Computational Biophysics and Imaging Group (CBIG) at Tampere University Foundation. An award for computer time was provided by the U.S. Department of Energy's (DOE) Innovative and Novel Computational Impact on Theory and Experiment (INCITE) Program; this research used supporting resources at the Argonne Leadership Computing Facilities at Argonne National Laboratory, which is supported by the Office of Science of the U.S. DOE under contract no. DE-AC02-06CH11357. Simulations were further enabled by a grant from the Swiss National Supercomputing Centre (CSCS) under project ID lp107 on Alps.

We would like to thank Dr. Max Cumberland and Dr. Albert Dasi for feedback and discussions.

Parts of this study were included in L.L.R's DPhil thesis at the University of Oxford, UK, titled Human-Based Multiscale Modeling and Simulation to Investigate Arrhythmias and Treatments in Ischemic Heart Disease (2024).

For the purpose of open access, the author has applied a Creative Commons Attribution (CC BY) public copyright license to any Author Accepted Manuscript version arising from this submission.

AUTHOR CONTRIBUTIONS

Conceptualization, methodology, investigation, writing – original draft, L.L.R.; conceptualization, methodology, writing – review and editing, Z.J.W.; methodology, writing – review and editing,

X.Z.; software, writing – review and editing, L.A.B.; conceptualization, writing – review and editing, supervision, C.T. and B.R.

DECLARATION OF INTERESTS

C.T. is an employee of AstraZeneca and may hold shares and/or stock options in the company.

STAR★METHODS

Detailed methods are provided in the online version of this paper and include the following:

- KEY RESOURCES TABLE
- METHOD DETAILS
  - Multiscale electrophysiological modelling and simulation of the chronically infarcted human ventricles with Purkinje
  - Simulating injection of day 0, day 14, and rapid stem cell-derived cardiomyocytes
  - Identifying and optimizing anti-arrhythmic ionic targets
  - Stimulation protocols to simulate sinus rhythm and quantify arrhythmic risk
  - Verification, validation, and uncertainty quantification

SUPPLEMENTAL INFORMATION

Supplemental information can be found online at https://doi.org/10.1016/j.stemcr.2026.103007.

Received: October 6, 2025
Revised: June 9, 2026
Accepted: June 10, 2026
Published: July 9, 2026

REFERENCES

Aronis, K.N., Ali, R.L., Prakosa, A., Ashikaga, H., Berger, R.D., Hakim, J.B., Liang, J., Tandri, H., Teng, F., Chrispin, J., and Trayanova, N.A. (2020). Accurate Conduction Velocity Maps and Their Association With Scar Distribution on Magnetic Resonance Imaging in Patients With Postinfarction Ventricular Tachycardias. Circ. Arrhythm. Electrophysiol. *13*, e007792. https://doi.org/10.1161/CIRCEP.119.007792.

Bayer, J.D., Gillette, K., Coronel, R., Plank, G., and Vigmond, E.J. (2025). To reconnect or not reconnect distal Purkinje fibers, that is the question when modeling the Purkinje fiber network. Front. Physiol. *16*, 1657611. https://doi.org/10.3389/fphys.2025.1657611.

Berg, L.A., Oliveira, R.S., Camps, J., de Lima, L.M.R., de Oliveira Campos, J., Wang, Z.J., Doste, R., Bueno-Orovio, A., dos Santos, R.W., and Rodriguez, B. (2026). Toward cardiac electrophysiology digital twins with an efficient open source scalable solver on GPU clusters. Sci. Rep. *16*, 9619. https://doi.org/10.1038/s41598-025-33709-w.

Berg, L.A., Rocha, B.M., Oliveira, R.S., Sebastian, R., Rodriguez, B., de Queiroz, R.A.B., Cherry, E.M., and Dos Santos, R.W. (2023). Enhanced optimization-based method for the generation of patient-specific models of Purkinje networks. Sci. Rep. *13*, 11788. https://doi.org/10.1038/s41598-023-38653-1.

Boukens, B.J., Sulkin, M.S., Gloschat, C.R., Ng, F.S., Vigmond, E.J., and Efimov, I.R. (2015). Transmural APD gradient synchronizes repolarization in the human left ventricular wall. Cardiovasc. Res. *108*, 188–196. https://doi.org/10.1093/cvr/cvv202.

Bueno-Orovio, A., Sánchez, C., Pueyo, E., and Rodriguez, B. (2014). Na/K pump regulation of cardiac repolarization: insights from a systems biology approach. Pflugers Arch - Eur J Physiol *466*, 183–193. https://doi.org/10.1007/s00424-013-1293-1.

Camps, J., Berg, L.A., Wang, Z.J., Sebastian, R., Riebel, L.L., Doste, R., Zhou, X., Sachetto, R., Coleman, J., Lawson, B., et al. (2024). Digital twinning of the human ventricular activation sequence to Clinical 12-lead ECGs and magnetic resonance imaging using realistic Purkinje networks for in silico clinical trials. Med. Image Anal. *94*, 103108. https://doi.org/10.1016/j.media.2024.103108.

Cardone-Noott, L., Bueno-Orovio, A., Minchole, A., Burrage, K., Wallman, M., Zemzemi, N., Dall'Armellina, E., and Rodriguez, B. (2014). A computational investigation into the effect of infarction on clinical human electrophysiology biomarkers. Computing in Cardiology (CinC) *2014*, 673–676.

Chong, J.J.H., Yang, X., Don, C.W., Minami, E., Liu, Y.-W., Weyers, J.J., Mahoney, W.M., Van Biber, B., Cook, S.M., Palpant, N.J., et al. (2014). Human embryonic-stem-cell-derived cardiomyocytes regenerate non-human primate hearts. Nature *510*, 273–277. https://doi.org/10.1038/nature13233.

Dasí, A., Nagel, C., Pope, M.T.B., Wijesurendra, R.S., Betts, T.R., Sachetto, R., Loewe, A., Bueno-Orovio, A., and Rodriguez, B. (2024). *In Silico* TRials guide optimal stratification of ATrIal FIbrillation patients to Catheter Ablation and pharmacological medicaTION: the i-STRATIFICATION study. Europace *26*, euae150. https://doi.org/10.1093/europace/euae150.

Dhahri, W., Sadikov Valdman, T., Wilkinson, D., Pereira, E., Ceylan, E., Andharia, N., Qiang, B., Masoudpour, H., Wulkan, F., Quesnel, E., et al. (2022). In Vitro Matured Human Pluripotent Stem Cell–Derived Cardiomyocytes Form Grafts With Enhanced Structure and Function in Injured Hearts. Circulation *145*, 1412–1426. https://doi.org/10.1161/CIRCULATIONAHA.121.053563.

Doste, R., Camps, J., Wang, Z.J., Berg, L.A., Holmes, M., Smith, H., Beetz, M., Li, L., Banerjee, A., Grau, V., and Rodriguez, B. (2026). An Automated Computational Pipeline for Generating Large-Scale Cohorts of Patient-Specific Ventricular Models in Electromechanical In Silico Trials. Comput. Methods Programs Biomed. *279*, 109290. https://doi.org/10.1016/j.cmpb.2026.109290.

Doste, R., Soto-Iglesias, D., Bernardino, G., Alcaine, A., Sebastian, R., Giffard-Roisin, S., Sermesant, M., Berruezo, A., Sanchez-Quintana, D., and Camara, O. (2019). A rule-based method to model myocardial fiber orientation in cardiac biventricular geometries with outflow tracts. Int. J. Numer. Method. Biomed. Eng. *35*, e3185. https://doi.org/10.1002/cnm.3185.

Faber, G.M., and Rudy, Y. (2000). Action Potential and Contractility Changes in $[Na^+]_i$ Overloaded Cardiac Myocytes: A Simulation Study. Biophys. J. *78*, 2392–2404. https://doi.org/10.1016/S0006-3495(00)76783-X.

Fassina, D., Costa, C.M., Longobardi, S., Karabelas, E., Plank, G., Harding, S.E., and Niederer, S.A. (2022). Modelling the interaction between stem cells derived cardiomyocytes patches and host myocardium to aid non-arrhythmic engineered heart tissue design. PLoS Comput. Biol. *18*, e1010030. https://doi.org/10.1371/journal.pcbi.1010030.

Fassina, D., M. Costa, C., Bishop, M., Plank, G., Whitaker, J., Harding, S.E., and Niederer, S.A. (2023). Assessing the arrhythmogenic risk of engineered heart tissue patches through in silico application on infarcted ventricle models. Comput. Biol. Med. *154*, 106550. https://doi.org/10.1016/j.compbiomed.2023.106550.

Franz, M.R., Bargheer, K., Rafflenbeul, W., Haverich, A., and Lichtlen, P.R. (1987). Monophasic action potential mapping in human subjects with normal electrocardiograms: direct evidence for the genesis of the T wave. Circulation *75*, 379–386. https://doi.org/10.1161/01.CIR.75.2.379.

Gibbs, C.E., and Boyle, P.M. (2026). Accelerated Intrinsic Beating Rate in Heterogeneously Coupled Human Pluripotent Stem Cell-Derived Cardiomyocytes Can Underlie Focal Ventricular Tachycardia in Regenerative Therapy. The Journal of Precision Medicine: Health and Disease *6*, 100035. https://doi.org/10.1016/j.premed.2026.100035.

Gibbs, C.E., Marchianó, S., Zhang, K., Yang, X., Murry, C.E., and Boyle, P.M. (2023). Graft-host coupling changes can lead to engraftment arrhythmia: A computational study. J. Physiol. *601*, 2733–2749. https://doi.org/10.1113/JP284244.

Goversen, B., van der Heyden, M.A.G., van Veen, T.A.B., and de Boer, T.P. (2018). The immature electrophysiological phenotype of iPSC-CMs still hampers in vitro drug screening: Special focus on I_{K1}. Pharmacol. Ther. *183*, 127–136. https://doi.org/10.1016/j.pharmthera.2017.10.001.

Gray, D.F., Mihailidou, A.S., Hansen, P.S., Buhagiar, K.A., Bewick, N.L., Rasmussen, H.H., and Whalley, D.W. (1998). Amiodarone inhibits the Na^+-K^+ pump in rabbit cardiac myocytes after acute and chronic treatment. J. Pharmacol. Exp. Ther. *284*, 75–82. https://doi.org/10.1016/S0022-3565(24)37204-0.

Haissaguerre, M., Vigmond, E., Stuyvers, B., Hocini, M., and Bernus, O. (2016). Ventricular arrhythmias and the His–Purkinje system. Nat. Rev. Cardiol. *13*, 155–166. https://doi.org/10.1038/nrcardio.2015.193.

Hansen, K.J., Laflamme, M.A., and Gaudette, G.R. (2018). Development of a Contractile Cardiac Fiber From Pluripotent Stem Cell Derived Cardiomyocytes. Front. Cardiovasc. Med. *5*, 52. https://doi.org/10.3389/fcvm.2018.00052.

He, J.-Q., Ma, Y., Lee, Y., Thomson, J.A., and Kamp, T.J. (2003). Human Embryonic Stem Cells Develop Into Multiple Types of Cardiac Myocytes. Circ. Res. *93*, 32–39. https://doi.org/10.1161/01.RES.0000080317.92718.99.

Henderson, S.A., Goldhaber, J.I., So, J.M., Han, T., Motter, C., Ngo, A., Chantawansri, C., Ritter, M.R., Friedlander, M., Nicoll, D.A., et al. (2004). Functional Adult Myocardium in the Absence of Na^+-Ca^{2+} Exchange. Circ. Res. *95*, 604–611. https://doi.org/10.1161/01.RES.0000142316.08250.68.

Hill, Y.R., Child, N., Hanson, B., Wallman, M., Coronel, R., Plank, G., Rinaldi, C.A., Gill, J., Smith, N.P., Taggart, P., and Bishop, M.J. (2016). Investigating a Novel Activation-Repolarisation Time Metric to Predict Localised Vulnerability to Reentry Using Computational Modelling. PLoS One *11*, e0149342. https://doi.org/10.1371/journal.pone.0149342.

Ismaili, D., Gurr, K., Horváth, A., Yuan, L., Lemoine, M.D., Schulz, C., Sani, J., Petersen, J., Reichenspurner, H., Kirchhof, P., et al. (2022). Regulation of APD and Force by the Na^+/Ca^{2+} Exchanger in Human-Induced Pluripotent Stem Cell-Derived Engineered Heart Tissue. Cells *11*, 2424. https://doi.org/10.3390/cells11152424.

Jamil-Copley, S., Vergara, P., Carbucicchio, C., Linton, N., Koa-Wing, M., Luther, V., Francis, D.P., Peters, N.S., Davies, D.W., Tondo, C., et al. (2015). Application of Ripple Mapping to Visualize Slow Conduction Channels Within the Infarct-Related Left Ventricular Scar. Circ. Arrhythm. Electrophysiol. *8*, 76–86. https://doi.org/10.1161/CIRCEP.114.001827.

Jebran, A.-F., Seidler, T., Tiburcy, M., Daskalaki, M., Kutschka, I., Fujita, B., Ensminger, S., Bremmer, F., Moussavi, A., Yang, H., et al. (2025). Engineered heart muscle allografts for heart repair in primates and humans. Nature *639*, 503–511. https://doi.org/10.1038/s41586-024-08463-0.

Kim, J.J., Yang, L., Lin, B., Zhu, X., Sun, B., Kaplan, A.D., Bett, G.C.L., Rasmusson, R.L., London, B., and Salama, G. (2015). Mechanism of automaticity in cardiomyocytes derived from human induced pluripotent stem cells. J. Mol. Cell. Cardiol. *81*, 81–93. https://doi.org/10.1016/j.yjmcc.2015.01.013.

Koivumäki, J.T., Naumenko, N., Tuomainen, T., Takalo, J., Oksanen, M., Puttonen, K.A., Lehtonen, Š., Kuusisto, J., Laakso, M., Koistinaho, J., and Tavi, P. (2018). Structural Immaturity of Human iPSC-Derived Cardiomyocytes: In Silico Investigation of Effects on Function and Disease Modeling. Front. Physiol. *9*, 80. https://doi.org/10.3389/fphys.2018.00080.

Koushik, S.V., Wang, J., Rogers, R., Moskophidis, D., Lambert, N.A., Creazzo, T.L., and Conway, S.J. (2001). Targeted inactivation of the sodium-calcium exchanger (Ncx1) results in the lack of a heartbeat and abnormal myofibrillar organization. FASEB J. *15*, 1209–1211. https://doi.org/10.1096/fj.00-0696fje.

Liu, Y.-W., Chen, B., Yang, X., Fugate, J.A., Kalucki, F.A., Futakuchi-Tsuchida, A., Couture, L., Vogel, K.W., Astley, C.A., Baldessari, A., et al. (2018). Human embryonic stem cell–derived cardiomyocytes restore function in infarcted hearts of non-human primates. Nat. Biotechnol. *36*, 597–605. https://doi.org/10.1038/nbt.4162.

Ma, J., Guo, L., Fiene, S.J., Anson, B.D., Thomson, J.A., Kamp, T.J., Kolaja, K.L., Swanson, B.J., and January, C.T. (2011). High purity human-induced pluripotent stem cell-derived cardiomyocytes: electrophysiological properties of action potentials and ionic currents. Am. J. Physiol. Heart Circ. Physiol. *301*, H2006–H2017. https://doi.org/10.1152/ajpheart.00694.2011.

Marchiano, S., Nakamura, K., Reinecke, H., Neidig, L., Lai, M., Kadota, S., Perbellini, F., Yang, X., Klaiman, J.M., Blakely, L.P., et al. (2023). Gene editing to prevent ventricular arrhythmias associated with cardiomyocyte cell therapy. Cell Stem Cell *30*, 396–414.e9. https://doi.org/10.1016/j.stem.2023.03.010.

Mincholé, A., Zacur, E., Ariga, R., Grau, V., and Rodriguez, B. (2019). MRI-Based Computational Torso/Biventricular Multiscale Models to Investigate the Impact of Anatomical Variability on

the ECG QRS Complex. Front. Physiol. *10*, 1103. https://doi.org/10.3389/fphys.2019.01103.

Musuamba, F.T., Skottheim Rusten, I., Lesage, R., Russo, G., Bursi, R., Emili, L., Wangorsch, G., Manolis, E., Karlsson, K.E., Kulesza, A., et al. (2021). Scientific and regulatory evaluation of mechanistic in silico drug and disease models in drug development: Building model credibility. CPT Pharmacometrics Syst. Pharmacol. *10*, 804–825. https://doi.org/10.1002/psp4.12669.

Nakamura, K., Neidig, L.E., Yang, X., Weber, G.J., El-Nachef, D., Tsuchida, H., Dupras, S., Kalucki, F.A., Jayabalu, A., Futakuchi-Tsuchida, A., et al. (2021). Pharmacologic therapy for engraftment arrhythmia induced by transplantation of human cardiomyocytes. Stem Cell Rep. *16*, 2473–2487. https://doi.org/10.1016/j.stemcr.2021.08.005.

Neco, P., Rose, B., Huynh, N., Zhang, R., Bridge, J.H.B., Philipson, K.D., and Goldhaber, J.I. (2010). Sodium-Calcium Exchange Is Essential for Effective Triggering of Calcium Release in Mouse Heart. Biophys. J. *99*, 755–764. https://doi.org/10.1016/j.bpj.2010.04.071.

Otsu, K., Kuruma, A., Yanagida, E., Shoji, S., Inoue, T., Hirayama, Y., Uematsu, H., Hara, Y., and Kawano, S. (2005). Na^+/K^+ ATPase and its functional coupling with Na^+/Ca^{2+} exchanger in mouse embryonic stem cells during differentiation into cardiomyocytes. Cell Calcium *37*, 137–151. https://doi.org/10.1016/j.ceca.2004.08.004.

Ottolia, M., Torres, N., Bridge, J.H.B., Philipson, K.D., and Goldhaber, J.I. (2013). Na/Ca exchange and contraction of the heart. J. Mol. Cell. Cardiol. *61*, 28–33. https://doi.org/10.1016/j.yjmcc.2013.06.001.

Ozdemir, S., Bito, V., Holemans, P., Vinet, L., Mercadier, J.-J., Varro, A., and Sipido, K.R. (2008). Pharmacological Inhibition of Na/Ca Exchange Results in Increased Cellular Ca^{2+} Load Attributable to the Predominance of Forward Mode Block. Circ. Res. *102*, 1398–1405. https://doi.org/10.1161/CIRCRESAHA.108.173922.

Paci, M., Koivumäki, J.T., Lu, H.R., Gallacher, D.J., Passini, E., and Rodriguez, B. (2021). Comparison of the Simulated Response of Three in Silico Human Stem Cell-Derived Cardiomyocytes Models and in Vitro Data Under 15 Drug Actions. Front. Pharmacol. *12*, 604713. https://doi.org/10.3389/fphar.2021.604713.

Paci, M., Passini, E., Klimas, A., Severi, S., Hyttinen, J., Rodriguez, B., and Entcheva, E. (2020). All-Optical Electrophysiology Refines Populations of In Silico Human iPSC-CMs for Drug Evaluation. Biophys. J. *118*, 2596–2611. https://doi.org/10.1016/j.bpj.2020.03.018.

Paci, M., Sartiani, L., Del Lungo, M., Jaconi, M., Mugelli, A., Cerbai, E., and Severi, S. (2012). Mathematical modelling of the action potential of human embryonic stem cell derived cardiomyocytes. Biomed. Eng. Online *11*, 61. https://doi.org/10.1186/1475-925X-11-61.

Passini, E., Zhou, X., Trovato, C., Britton, O.J., Bueno-Orovio, A., and Rodriguez, B. (2021). The virtual assay software for human in silico drug trials to augment drug cardiac testing. J. Comput. Sci. *52*, 101202. https://doi.org/10.1016/j.jocs.2020.101202.

Poch, C.M., Foo, K.S., De Angelis, M.T., Jennbacken, K., Santamaria, G., Bähr, A., Wang, Q.-D., Reiter, F., Hornaschewitz, N., Zawada, D., et al. (2022). Migratory and anti-fibrotic programmes define the regenerative potential of human cardiac progenitors. Nat. Cell Biol. *24*, 659–671. https://doi.org/10.1038/s41556-022-00899-8.

Reindl, M., Holzknecht, M., Tiller, C., Lechner, I., Schiestl, M., Simma, F., Pamminger, M., Henninger, B., Mayr, A., Klug, G., et al. (2020). Impact of infarct location and size on clinical outcome after ST-elevation myocardial infarction treated by primary percutaneous coronary intervention. Int. J. Cardiol. *301*, 14–20. https://doi.org/10.1016/j.ijcard.2019.11.123.

Riebel, L.L., Passini, E., Margara, F., Paci, M., Biasetti, J., and Rodriguez, B. (2021). In Silico Identification of the Key Ionic Currents Modulating Human Pluripotent Stem Cell-Derived Cardiomyocytes Towards an Adult Phenotype. Computing in Cardiology (CinC) *2021*, 1–4. https://doi.org/10.23919/CinC53138.2021.9662683.

Riebel, L.L., Wang, Z.J., Martinez-Navarro, H., Trovato, C., Camps, J., Berg, L.A., Zhou, X., Doste, R., Sachetto Oliveira, R., Weber dos Santos, R., et al. (2024). In silico evaluation of cell therapy in acute versus chronic infarction: role of automaticity, heterogeneity and Purkinje in human. Sci. Rep. *14*, 21584. https://doi.org/10.1038/s41598-024-67951-5.

Ringenberg, J., Deo, M., Filgueiras-Rama, D., Pizarro, G., Ibañez, B., Peinado, R., Merino, J.L., Berenfeld, O., and Devabhaktuni, V. (2014). Effects of Fibrosis Morphology on Reentrant Ventricular Tachycardia Inducibility and Simulation Fidelity in Patient-Derived Models. Clin. Med. Insights Cardiol. *8*, 8s1. https://doi.org/10.4137/CMC.S15712.

Rog-Zielinska, E.A., Norris, R.A., Kohl, P., and Markwald, R. (2016). The Living Scar – Cardiac Fibroblasts and the Injured Heart. Trends Mol. Med. *22*, 99–114. https://doi.org/10.1016/j.molmed.2015.12.006.

Romagnuolo, R., Masoudpour, H., Porta-Sánchez, A., Qiang, B., Barry, J., Laskary, A., Qi, X., Massé, S., Magtibay, K., Kawajiri, H., et al. (2019). Human Embryonic Stem Cell-Derived Cardiomyocytes Regenerate the Infarcted Pig Heart but Induce Ventricular Tachyarrhythmias. Stem Cell Rep. *12*, 967–981. https://doi.org/10.1016/j.stemcr.2019.04.005.

Romero, L., Pueyo, E., Fink, M., and Rodríguez, B. (2009). Impact of ionic current variability on human ventricular cellular electrophysiology. Am. J. Physiol. Heart Circ. Physiol. *297*, H1436–H1445. https://doi.org/10.1152/ajpheart.00263.2009.

Rosales, R.M., Mountris, K.A., Oliván-Viguera, A., Pérez-Zabalza, M., Cedillo-Servin, G., Iglesias-García, O., Hrynevich, A., Castilho, M., Malda, J., Prósper, F., et al. (2024). Experimentally-guided in silico design of engineered heart tissues to improve cardiac electrical function after myocardial infarction. Comput. Biol. Med. *171*, 108044. https://doi.org/10.1016/j.compbiomed.2024.108044.

Sachetto Oliveira, R., Martins Rocha, B., Burgarelli, D., Meira, W., Constantinides, C., and Weber dos Santos, R. (2018). Performance evaluation of GPU parallelization, space-time adaptive algorithms, and their combination for simulating cardiac electrophysiology. Int. J. Numer. Method. Biomed. Eng. *34*, e2913. https://doi.org/10.1002/cnm.2913.

Sertkaya, A., Beleche, T., Jessup, A., and Sommers, B.D. (2024). Costs of Drug Development and Research and Development

Intensity in the US, 2000-2018. JAMA Netw. Open *7*, e2415445. https://doi.org/10.1001/jamanetworkopen.2024.15445.

Shiba, Y., Gomibuchi, T., Seto, T., Wada, Y., Ichimura, H., Tanaka, Y., Ogasawara, T., Okada, K., Shiba, N., Sakamoto, K., et al. (2016). Allogeneic transplantation of iPS cell-derived cardiomyocytes regenerates primate hearts. Nature *538*, 388–391. https://doi.org/10.1038/nature19815.

Spath, N.B., Singh, T., Papanastasiou, G., Baker, A., Janiczek, R.J., McCann, G.P., Dweck, M.R., Kershaw, L., Newby, D.E., and Semple, S. (2021). Assessment of stunned and viable myocardium using manganese-enhanced MRI. Open Heart *8*, e001646. https://doi.org/10.1136/openhrt-2021-001646.

Stevens, S.G., and Brown, C.M. (2013). In Silico Estimation of Translation Efficiency in Human Cell Lines: Potential Evidence for Widespread Translational Control. PLoS One *8*, e57625. https://doi.org/10.1371/journal.pone.0057625.

Taggart, P., Sutton, P.M., Opthof, T., Coronel, R., Trimlett, R., Pugsley, W., and Kallis, P. (2000). Inhomogeneous Transmural Conduction During Early Ischaemia in Patients with Coronary Artery Disease. J. Mol. Cell. Cardiol. *32*, 621–630. https://doi.org/10.1006/jmcc.2000.1105.

Telle, Å., Kassar, A., Chamoun, N., Haykal, R., Gonzalo, A., Hensley, T., Chahine, Y., Flores, O., Del Álamo, J.C., Akoum, N., et al. (2025). Systematic computational assessment of atrial function impairment due to fibrotic remodeling in electromechanical properties. PLoS Comput. Biol. *21*, e1013265. https://doi.org/10.1371/journal.pcbi.1013265.

Tomek, J., Bueno-Orovio, A., Passini, E., Zhou, X., Minchole, A., Britton, O., Bartolucci, C., Severi, S., Shrier, A., Virag, L., et al. (2019). Development, calibration, and validation of a novel human ventricular myocyte model in health, disease, and drug block. eLife *8*, e48890. https://doi.org/10.7554/eLife.48890.

Trovato, C., Longobardi, S., Passini, E., Beattie, K.A., Holmes, M., Chaudhary, K.W., Rossman, E.I., and Rodriguez, B. (2025). In silico predictions of drug-induced changes in human cardiac contractility align with experimental recordings. Front. Pharmacol. *16*, 1500668. https://doi.org/10.3389/fphar.2025.1500668.

Trovato, C., Mohr, M., Schmidt, F., Passini, E., and Rodriguez, B. (2022). Cross clinical-experimental-computational qualification of in silico drug trials on human cardiac purkinje cells for proarrhythmia risk prediction. Front. Toxicol. *4*, 992650. https://doi.org/10.3389/ftox.2022.992650.

Trovato, C., Passini, E., Nagy, N., Varró, A., Abi-Gerges, N., Severi, S., and Rodriguez, B. (2020). Human Purkinje in silico model enables mechanistic investigations into automaticity and proarrhythmic abnormalities. J. Mol. Cell. Cardiol. *142*, 24–38. https://doi.org/10.1016/j.yjmcc.2020.04.001.

Verkerk, A.O., and Wilders, R. (2023). Injection of I_{K1} through dynamic clamp can make all the difference in patch-clamp studies on hiPSC-derived cardiomyocytes. Front. Physiol. *14*, 1326160. https://doi.org/10.3389/fphys.2023.1326160.

Viceconti, M., Emili, L., Afshari, P., Courcelles, E., Curreli, C., Famaey, N., Geris, L., Horner, M., Jori, M.C., Kulesza, A., et al. (2021). Possible Contexts of Use for In Silico Trials Methodologies: A Consensus-Based Review. IEEE J. Biomed. Health Inform. *25*, 3977–3982. https://doi.org/10.1109/JBHI.2021.3090469.

Viceconti, M., Pappalardo, F., Rodriguez, B., Horner, M., Bischoff, J., and Musuamba Tshinanu, F. (2021). In silico trials: Verification, validation and uncertainty quantification of predictive models used in the regulatory evaluation of biomedical products. Methods *185*, 120–127. https://doi.org/10.1016/j.ymeth.2020.01.011.

Watanabe, Y., and Kimura, J. (2000). Inhibitory effect of amiodarone on Na^+/Ca^{2+} exchange current in guinea-pig cardiac myocytes. Br. J. Pharmacol. *131*, 80–84. https://doi.org/10.1038/sj.bjp.0703527.

Yu, J.K., Liang, J.A., Franceschi, W.H., Huang, Q., Pashakhanloo, F., Sung, E., Boyle, P.M., and Trayanova, N.A. (2022). Assessment of arrhythmia mechanism and burden of the infarcted ventricles following remuscularization with pluripotent stem cell-derived cardiomyocyte patches using patient-derived models. Cardiovasc. Res. *118*, 1247–1261. https://doi.org/10.1093/cvr/cvab140.

Zhou, X., Wang, Z.J., Camps, J., Tomek, J., Santiago, A., Quintanas, A., Vazquez, M., Vaseghi, M., and Rodriguez, B. (2024). Clinical phenotypes in acute and chronic infarction explained through human ventricular electromechanical modelling and simulations. eLife *13*, RP93002. https://doi.org/10.7554/eLife.93002.3.

STAR★METHODS

KEY RESOURCES TABLE

REAGENT or RESOURCE	SOURCE	IDENTIFIER
**Deposited data**		
Healthy biventricular human mesh with Purkinje tree and fiber directions	Camps et al. (2024)	https://doi.org/10.5281/zenodo.14046617
Human biventricular meshes with synthetic scars	Riebel et al. (2024)	https://doi.org/10.5281/zenodo.14699735
Human biventricular meshes after virtual cell injection	Riebel et al. (2024)	https://doi.org/10.5281/zenodo.14699735 (using the mesh with homogeneous ventricular-like virtual cell delivery)
**Software and algorithms**		
MATLAB	MathWorks	Version R2022b
ToR-ORd model of human ventricular cardiomyocytes	Tomek et al. (2019)	https://github.com/jtmff/torord (version model_Torord_dynCl_updatedPhiCaL.m)
Post-MI ToR-ORd model of human ventricular cardiomyocytes	Zhou et al. (2024)	https://github.com/xinzhoujoy/HumanPostMIVentricularCellElectromechanics
Trovato model of human Purkinje cells	Trovato et al. (2020)	https://www.cs.ox.ac.uk/insilicocardiotox/purkinje-models and https://github.com/rsachetto/MonoAlg3D_C/tree/master/src/models_library/trovato
Paci2020 model of stem cell-derived cardiomyocytes	Paci et al. (2020)	www.mcbeng.it/en/category/software.html
MonoAlg3D simulation software	Berg et al., 2026; Sachetto Oliveira et al. 2018	https://github.com/rsachetto/MonoAlg3D_C
Custom MonoAlg3D functions	This study and Riebel et al. (2024)	https://github.com/LLRiebel/MonoAlg3D_C-2023
Digital twinning pipeline to add fiber fields, transmurality, and apicobasal gradient in the mesh	Doste et al., 2026	https://github.com/rdoste/InSilicoHeartGen
Purkinje network generation tool	Berg et al. (2023)	https://github.com/bergolho/Shocker
ParaView	Kitware Inc	Version 5.13.2

METHOD DETAILS

Multiscale electrophysiological modelling and simulation of the chronically infarcted human ventricles with Purkinje

Electrophysiological activity of the infarcted human ventricles was simulated from subcellular dynamics to the ECG as in Riebel et al. (2024), see Figure S8. Briefly, a healthy human biventricular geometry was reconstructed from MRI by Mincholé et al. (2019). Next, fibre orientations were introduced as described in Doste et al. (2019) and conduction velocity in fibre orientation calibrated to 65 cm/s, as observed clinically (Taggart et al., 2000). Sheet and sheet-normal conduction

velocity was inferred (38 and 47 cm/s, respectively) and a Purkinje network created to reproduce the patient's QRS complex (Camps et al., 2024), see Figure S8A.

Membrane kinetics were simulated with the state-of-the-art human ventricular cardiomyocyte and Purkinje cell ToR-ORd and Trovato models, respectively (Tomek et al., 2019; Trovato et al., 2020). Based on clinical observations (Boukens et al., 2015; Franz et al., 1987), biventricular electrophysiological gradients of 35 ms transmurally and 10 ms from apex to base were introduced by scaling key ionic current conductances (summarised in Table S1).

To model MI, three physiological scar geometries (available at https://doi.org/10.5281/zenodo.14699735) were introduced into the healthy geometry using an established algorithm (Cardone-Noott et al., 2014; Hill et al., 2016). Briefly, to model anteroseptal infarction, six landmarks were selected to model the path of the left anterior descending artery along the anterior and septal walls and the apex. By keeping these landmark coordinates constant but changing the volume of infarction in the mesh, we created three different geometries of anteroseptal infarction. In line with clinically reported scar sizes (Reindl et al., 2020; Spath et al., 2021), resulting scars and border zones composed 14%, 23%, and 27% of the ventricles (referred to as the small, medium, and large scar, respectively).

For remote zone and border zone electrophysiology, key ion channel conductances and time constants were scaled based on experimental observations as described by Zhou et al. (2024) and summarised in Table S2; resulting action potentials are shown in Figure S8A. The scar was modelled as electrically inactive but conducting (Rog-Zielinska et al., 2016). Hence, the membrane potential was initialised to -60 mV and allowed to change only through terms of diffusivity (Ringenberg et al., 2014). Electrophysiological changes caused remote zone conduction velocity to decrease to 49, 27, and 34 cm/s in fibre, sheet, and sheet-normal direction, respectively. Compared to the remote zone, conductivities were calibrated to achieve an approximately 60% and 80% reduced conduction velocity in the border and infarct zone, respectively (see Table S3), based on clinical observations (Aronis et al., 2020; Jamil-Copley et al., 2015). The resulting simulated ECG traces were compared to clinical data of post-MI subjects in our previous study (Riebel et al., 2024) and reproduced persistent ST-segment elevation, QT-interval prolongation, and normalised T-waves (see Figure S9A).

Multiscale, electrophysiological simulations were performed using the monodomain model with the finite volume GPU-enabled open-source solver MonoAlg3D (Berg et al., 2026; Sachetto Oliveira et al., 2018) with parameters listed in Table S4 and a customised version of the simulator available at https://github.com/LLRiebel/MonoAlg3D_C-2023.

Simulating injection of day 0, day 14, and rapid stem cell-derived cardiomyocytes

As shown in Figure S8B, to investigate transient automaticity-induced arrhythmias, we constructed a D14 hPSC-CM model by modifying ionic current conductances of the baseline Paci2020 model (Paci et al., 2020), which we consider the D0 model. For the D14 model, experimentally reported *in vivo* gene expression data (Marchiano et al., 2023) was consulted to derive ion channel scaling factors. As data to exactly relate ion channel conductance from gene expression is lacking, to qualitatively capture the cell's maturation, we estimated the relative change in gene expression from Marchiano et al. (2023) and multiplied it by 0.25 to give a scaling factor for the corresponding ion channel conductance, as summarised in Table S5. This scaling factor of 0.25 accounts for the differences between the transcription and the translation level, considering factors such as translation efficiency, protein stability, and regulatory feedback (Stevens and Brown, 2013). Since large variability between spontaneous beating frequencies has been reported (He et al., 2003; Ma et al., 2011), we created an additional rapid beating hPSC-CM model by scaling the funny current, the sodium-calcium exchanger, and the sarco-endoplasmic reticulum calcium ATPase by 2.5 (resulting action potential biomarkers are shown in Table S6).

In 3D, cell injection was spatially modelled as in our previous study (Riebel et al., 2024), with meshes available at https://doi.org/10.5281/zenodo.14699735. Briefly, six injection locations were chosen within the infarct and maintained for the small, medium, and large scar. The probability for an adult ventricular cell to be replaced by an hPSC-CM decreased with increasing distance from the injection location, assuming low migration of hPSC-CMs as observed experimentally in chronic scars (Poch et al., 2022). The hPSC-CM distribution transmurally and in the infarct versus border zone was calibrated to experimental measurements (Poch et al., 2022) – particularly, hPSC-CMs were more likely to virtually settle in the subendocardium and in the infarct rather than the border zone. No hPSC-CMs were placed in the right ventricle or the remote zone. For each scar, the spatial hPSC-CM distribution was maintained across simulations including from D0 to D14. Conductivities were calibrated to achieve a 10 cm/s conduction velocity in hPSC-CMs at D0 and maintained for all other hPSC-CM models (see Table S3). This resulted in an increased conduction velocity of 14.5 cm/s at D14, similar to experimental observations (Hansen et al., 2018).

Identifying and optimizing anti-arrhythmic ionic targets

To identify suitable anti-arrhythmic targets, a sensitivity analysis of the spontaneously beating (i.e., unpaced) Paci2020 model was performed as previously described (Riebel et al., 2021). Briefly, each ion channel's conductance was scaled by +/- 25% and the relative sensitivities on key action potential biomarkers including upstroke velocity, peak membrane potential, action potential duration at 90% repolarisation (APD_{90}), maximum diastolic potential, and spontaneous beating frequency were computed as in Romero et al. (2009). The resulting relative sensitivities varied between -1 (strong negative correlation) and +1 (strong positive correlation).

Once targets to suppress automaticity were identified in the sensitivity analysis, we aimed to determine optimal combinations of ionic targets that also reduce re-entry susceptibility while preserving calcium transient amplitude. For this, a population of 1,458 D0 and D14 hPSC-CM models was created by simultaneously 1) blocking the funny current, 2) upregulating the inward rectifier potassium current's conductance and maximum sodium-potassium pump current between 1 and 3 in steps of 0.25, and 3) upregulating the rapid delayed outward rectifier potassium current's conductance between 1 and 5 in steps of 0.5. Spontaneously beating models were discarded and in the remaining population, an optimisation index incorporating both safety (measured by action potential duration) and efficacy (measured by calcium transient amplitude (CaT_{Amp})) was calculated as:

$$optimisation\ index\ =$$

$$\frac{w_{safety} * \left(1 - \frac{\left|APD_{90,target} - APD_{90}\right|}{APD_{90,target}}\right) + w_{efficacy} * \left(1 - \frac{\left|CaT_{Amp,target} - CaT_{Amp}\right|}{CaT_{Amp,target}}\right)}{w_{safety} + w_{efficacy}}$$

Here, the weights w_{safety} and $w_{efficacy}$ were set to 1.5 and 1, respectively, to prioritise safety. Target action potential duration can be used to create different hPSC-CM action potential models; here we aimed towards adult values to reduce repolarisation gradients. Target calcium transient amplitude was always set to the maximum calcium transient amplitude achieved across the population (excluding any spontaneously beating models).

Modifying ionic current expression in hPSC-CMs has been achieved through gene editing techniques before injection (Marchiano et al., 2023). Hence, we combined optimisation indices at D0 and D14 for each ionic scaling combination, using also the maximum optimisation indices $index_{D0,max}$ and $index_{D14,max}$:

$$combined\ optimisation\ index\ =\ \left(\frac{index_{D0}}{index_{D0,max}} + \frac{index_{D14}}{index_{D14,max}}\right) \bigg/ 2$$

Stimulation protocols to simulate sinus rhythm and quantify arrhythmic risk

The adult ventricular cardiomyocyte ToR-ORd model (Tomek et al., 2019), with remote, border, or infarct zone remodelling applied (Zhou et al., 2024), and the Purkinje cell Trovato2020 model (Trovato et al., 2020) were paced in virtual isolation at 1 Hz until steady state for 500 and 1,000 s, respectively, and then introduced into the biventricular model. As full coupling and therefore direct pacing of the hPSC-CMs in the ventricles could not be assumed, the hPSC-CM Paci2020 model (Paci et al., 2020), including D0, D14, rapid, and optimised variations, were simulated in isolation under spontaneous (i.e., unpaced) conditions for 1,000 s before being introduced into the ventricles. The biventricular model was then paced for three 1 Hz beats, applied at the top 25 elements of the bundle of His.

To assess automaticity-induced arrhythmias, biventricular models with either D0, rapid D0, D14, or rapid D14 hPSC-CMs were simulated without pacing for two seconds. If spontaneous beats occurred, ten 1 Hz beats were simulated to assess whether automaticity-induced arrhythmias would emerge under sinus pacing. To quantify re-entry burden, an S1-S2 protocol was employed: after the initial three 1 Hz beats (i.e., S1 stimuli), an S2 stimulus was applied in 15 different border zone locations between 350 to 570 ms after the S1. The different S2 locations were selected manually around the core of the small, medium, and large scars. Re-entry risk was measured as the vulnerable window (i.e., the range of S1-S2 intervals for which arrhythmias could be induced); considering S1-S2 intervals were increased in 20 ms increments, vulnerable windows were a multiple of 20.

This set up resulted in 1,458 single cell simulations to determine optimal ionic target combinations (9 scalings for the inward rectifier potassium current's conductance * 9 scalings of the maximum sodium-potassium pump current * 9 scalings for the rapid delayed outward rectifier potassium current's conductance * 2 hPSC-CM models, i.e., D0 and D14) and 1,806 biventricular simulations (12 S1-S2 intervals * 15 S2 locations * (3 scars before cell injection + 3 scars at D0 + large scar at D14 + large scar with 3 (2 at D0, 1 at D14) optimised hPSC-CM models) + 6 simulations of spontaneous activity).

Verification, validation, and uncertainty quantification

To establish trust in our modelling and simulation framework to mechanistically investigate cell therapy in chronic MI, we considered verification, validation, and uncertainty quantification principles (Musuamba et al., 2021; Viceconti et al., 2021). Verification of the monodomain solver was provided through benchmark tests (Berg et al., 2026; Sachetto Oliveira et al., 2018) and comparison to cell model solvers (Riebel et al., 2024).

Validation of the cellular models was demonstrated through incorporation of biophysiological knowledge and experimental evidence of human cardiac cells in the original publications (Paci et al., 2020; Tomek et al., 2019; Trovato et al., 2020). Furthermore, predictive accuracy of these models has been established through *in silico* drug trials on contractility as well as arrhythmogenicity (Paci et al., 2021; Passini et al., 2021; Trovato et al., 2022, 2025). Electrophysiological and structural remodelling post-MI was informed through experimental and clinical evidence as outlined above. Additionally, trust in the biventricular post-MI model was provided through comparison of simulated ECGs with clinical data (Riebel et al., 2024; Zhou et al., 2024).

In addition to previous sensitivity and convergence analyses (Riebel et al., 2024), we carried out further uncertainty quantification into the effects of steady state pacing duration and ionic concentrations on hPSC-CM action potentials at D0 and D14, as shown in Figures S10 and S11. These analyses showed small changes (less than 10%) in action potential duration at 90% repolarisation.

Stem Cell Reports, Volume *21*

Supplemental Information

In silico optimization of regenerative cell therapy in the infarcted human ventricles to mitigate arrhythmic burden

Leto Luana Riebel, Zhinuo Jenny Wang, Xin Zhou, Lucas Arantes Berg, Cristian Trovato, and Blanca Rodriguez

Supplementary Material

Contents

S1. Arrhythmic risk after virtual cell delivery 1
S2. Identifying anti-arrhythmic targets for hPSC-CMs 2
S3. Human electrophysiological modelling and simulation of chronic MI 8
S4. Modelling and simulation of cell therapy 9
S5. Verification, validation, and uncertainty quantification...12
Supplemental References 14

S1. Arrhythmic risk after virtual cell delivery

Figure S1 below supplements Figure 1 in the main text to highlight that no automaticity-induced arrhythmias were observed after virtual injection of the baseline hPSC-CMs at D0 or D14 as well as the rapidly beating hPSC-CMs at D0.

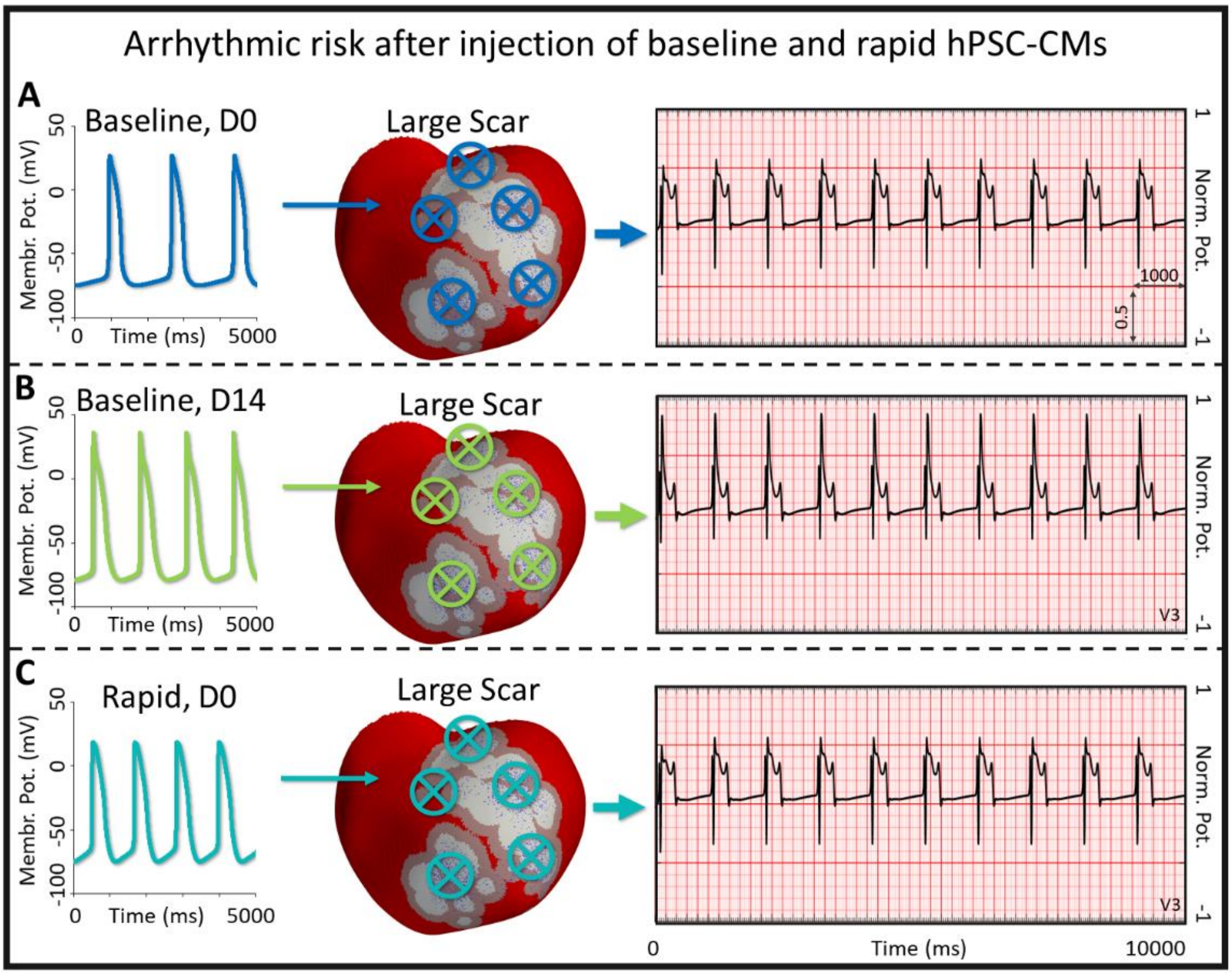

Figure S1. Absence of automaticity-induced arrhythmia burden after virtual injection of baseline hPSC-CMs at D0 (panel A) and D14 (panel B) as well as rapid hPSC-CMs at D14 (panel C). Simulations were performed in a human biventricular model

with Purkinje and a large synthetic anteroseptal MI scar. Left: AP traces of the different hPSC-CM models beating spontaneously in virtual isolation. Middle: five out of six virtual hPSC-CM injection locations highlighted with crosses in the biventricular mesh (the remaining one is located in the septum). Right: ECG lead V3 traces after virtual cell injection during 1 Hz stimulation at the bundle of His (note the absence of arrhythmias). Related to main text Figure 1.

S2. Identifying anti-arrhythmic targets for hPSC-CMs

As shown in Figure S2 below, sensitivity analyses showed the D0 model's spontaneous beating rate most sensitive to p_{NaK} (maximum sodium potassium pump current). Scaling p_{NaK} by 0.7 and 0.85 was sufficient to abolish spontaneous activity in the D0 and D14 phenotype, respectively.

Sensitivity analysis of unpaced hPSC-CMs

Parameter	MDP	dV/dt_{max}	V_{peak}	APD_{90}	Frequency
$V_{max,up}$		0.67			0.51
G_{CaL}		0.84	0.89	1.00	
k_{NaCa}			0.26		0.37
G_{NaF}		0.99			
G_{NaL}					
P_{NaK}		-1.00	1.00	0.25	-1.00
G_{to}					
G_{Kr}			-0.29	-0.90	
G_{Ks}					
G_{K1}	1.00	0.40	-0.41	-0.74	
G_f		-0.46		-0.29	0.35

Relative sensitivity < ±0.25 | Strong negative relation | Strong positive relation

Figure S2. Sensitivity analysis of the spontaneous computational D0 (i.e., baseline Paci2020 (Paci et al., 2020)) hPSC-CM model to changes in ionic current conductances. Shown are relative sensitivities (1: strong positive relation, -1: strong negative relation), calculated as in Romero et al. (2009). Conductances were scaled by +/- 25% and hPSC-CMs simulated to beat spontaneously for 1,000 s.

Figure S3, Figure S4, and Figure S5 below show ionic current and concentration traces underlying hPSC-CMs at D0 and D14 with and without scaling p_{NaK}, G_{K1}, and G_f. Note, in the hPSC-CM model with reduced p_{NaK} at D14 (Figure S4 A), the model had to be paced for 2000 rather than 1000 beats to reach steady state.

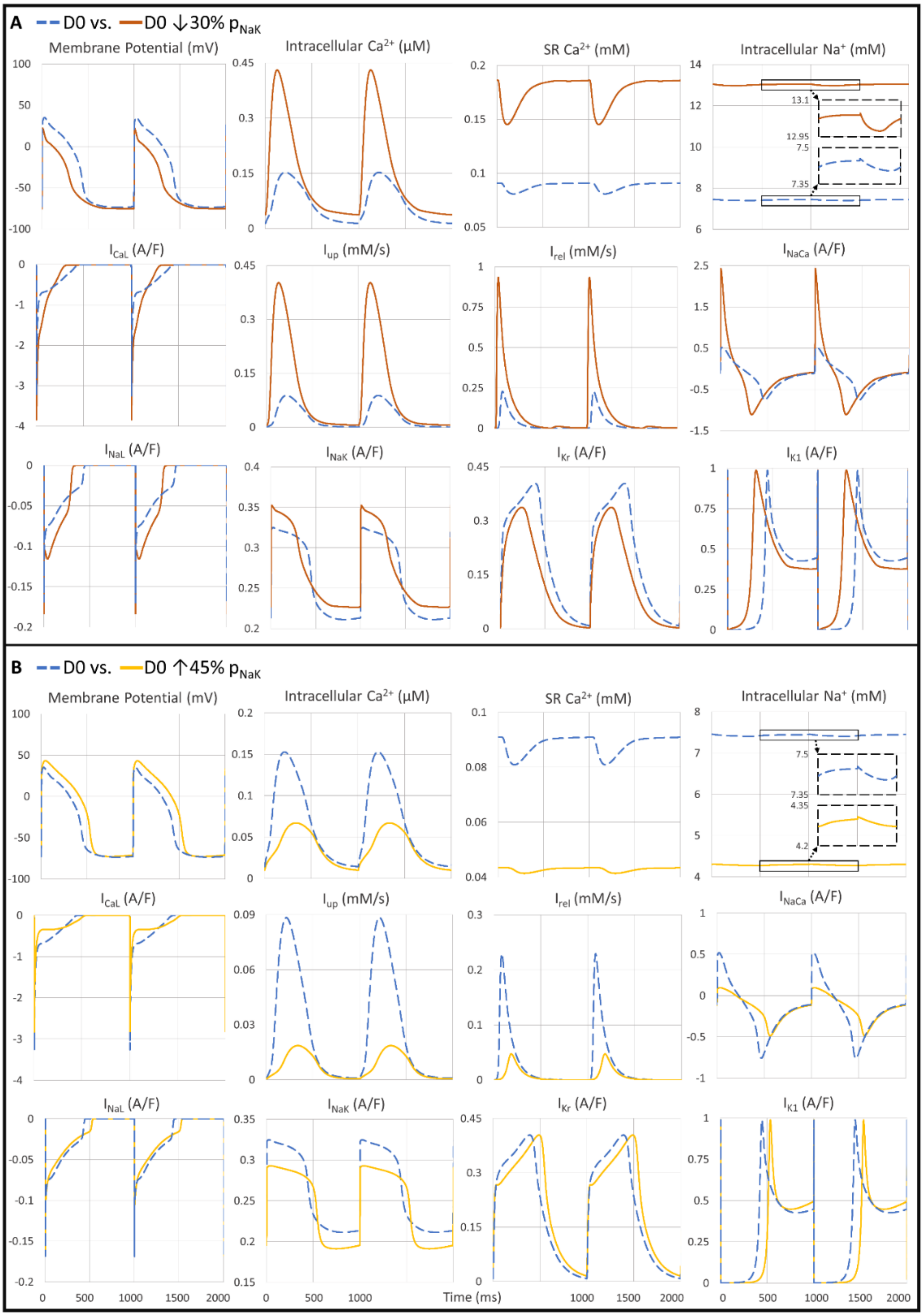

Figure S3. Simulated action potential and ionic current traces of baseline D0 hPSC-CMs in isolation after 1,000 paced 1 Hz beats. A) With and without downregulation of p_{NaK} (maximum Na^{+}-K^{+} pump current (I_{NaK})). B) With and without upregulation of p_{NaK}. Further shown are intracellular and SR Ca^{2+} concentrations, intracellular Na^{+} concentrations, I_{CaL}: L-type Ca^{2+} current, I_{up}: SERCA pump current, I_{rel}: SR Ca^{2+} release current, I_{NaCa}: Na^{+}-Ca^{2+} exchanger current, I_{NaL}: late Na^{+} current, I_{Kr}: rapid delayed outward K^{+} current, and I_{K1}: inward rectifier K^{+} current. Related to main text Figure 4.

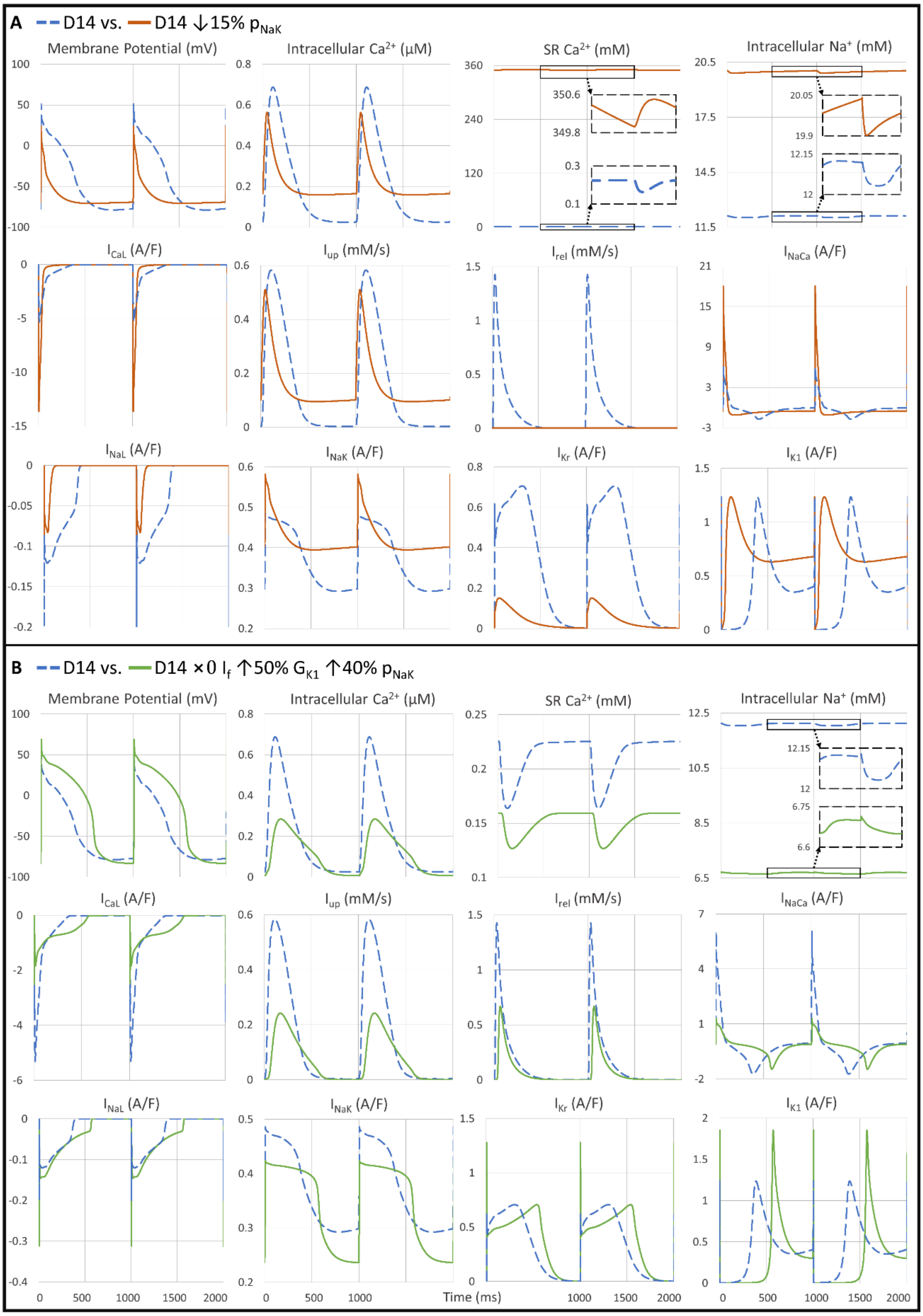

Figure S4. Simulated action potential and ionic current traces of baseline D14 hPSC-CMs in isolation after 1,000 paced 1 Hz beats. A) With and without downregulation of p_{NaK} (maximum Na^+-K^+ pump current (I_{NaK})); note that 2,000 beats were needed to achieve steady state in the D14 model with low p_{NaK} with the last beat shown here. B) With and without upregulation of p_{NaK} plus full block of the funny current (I_f) and upregulation of the inward rectifier K^+ current's (I_{K1}) conductance. Further shown are intracellular and SR Ca^{2+} concentrations, intracellular Na^+ concentrations, I_{CaL}: L-type Ca^{2+} current, I_{up}: SERCA pump current, I_{rel}: SR Ca^{2+} release current (effectively 0 when reducing p_{NaK}), I_{NaCa}: Na^+-Ca^{2+} exchanger current, I_{NaL}: late Na^+ current, and I_{Kr}: rapid delayed outward K^+ current. Related to main text Figure 4.

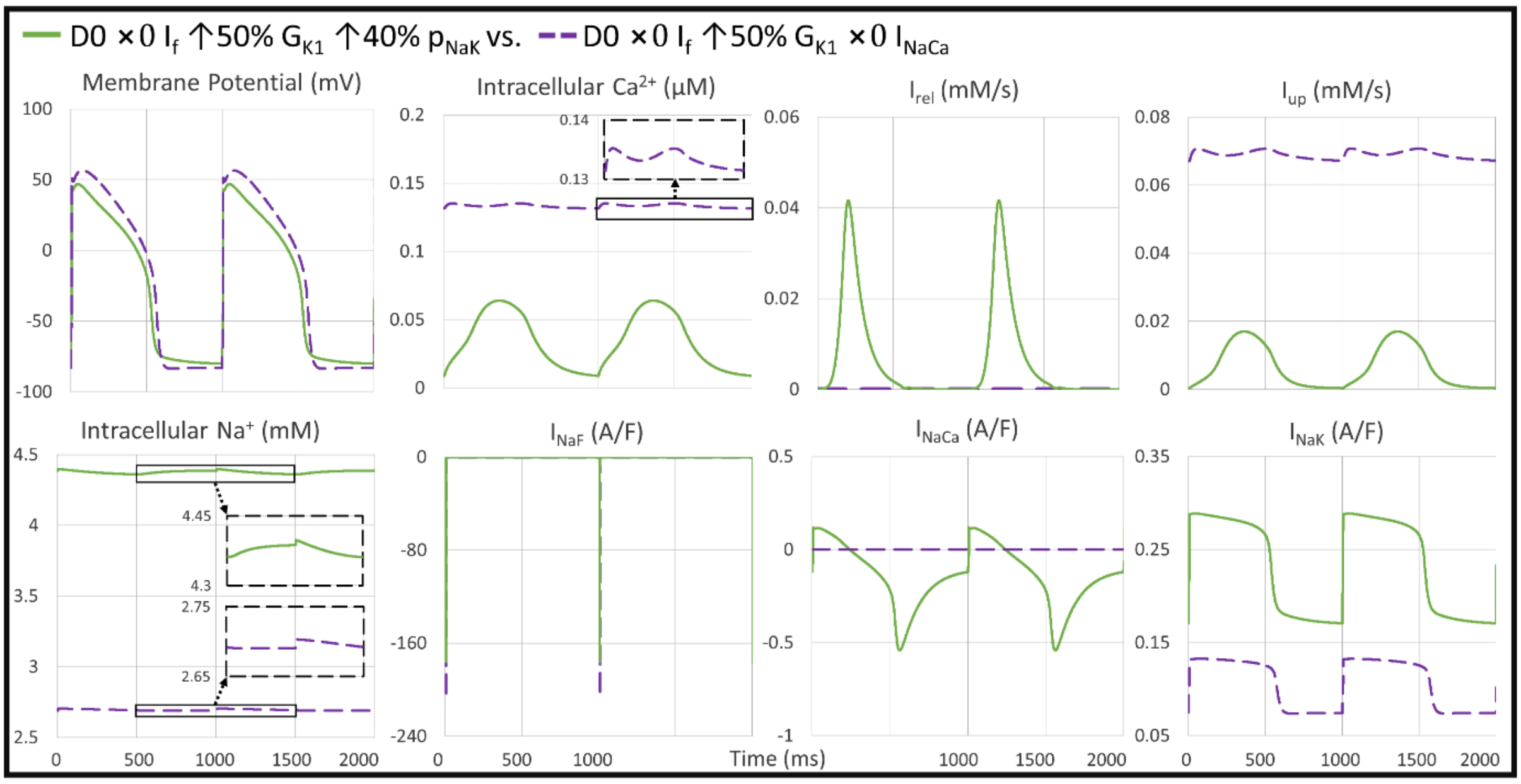

Figure S5. Simulated action potential and ionic current traces after 1,000 paced 1 Hz beats of baseline D0 hPSC-CMs with full block of the funny current (I_f) and upregulation of the inward rectifier K⁺ current's (I_{K1}) conductance plus either upregulation of p_{NaK} (maximum Na⁺-K⁺ pump current (I_{NaK})), green solid traces, or full block of the Na⁺-Ca²⁺ exchanger current (I_{NaCa}, as suggested by Marchiano et al. (2023)), purple dotted traces. Further shown are intracellular Ca²⁺ and Na⁺ concentrations, I_{rel}: SR Ca²⁺ release current (effectively 0 for I_{NaCa} block), I_{up}: SERCA pump current, and I_{NaF}: fast Na⁺ current. Related to main text Figure 4.

From the results presented in Figure S6, we chose the best performing ionic target combination. For Figure S7, we chose the ionic scaling combination with the third best result: fully blocking I_f together with upregulating G_{K1} by 1.25, p_{NaK} by 1.75, and G_{Kr} by 5, referred to in the text as "optimised" hPSC-CMs. We eliminated the top performing combination because it showed small beat-to-beat variations. We further discarded the second-best combination as it suggested that no G_{K1} scaling was necessary, which may be specific to our virtual cell types. As the differences in action potential duration at 90% repolarisation (APD_{90}) and calcium transient amplitude between the second and third-best options were only 2 ms and 0.001 μM, respectively, we finally chose to proceed with the third option that maintained G_{K1} scaling at 1.25. The optimised cells at D0 and D14 had an APD_{90} of 169 ms and 230 ms, respectively.

GK1	GKr \ PNaK	1.5	1.75	2	2.25	2.5	2.75	3
1	1							
	1.5							
	2							
	2.5		0.86	0.84	0.83	0.81	0.81	0.80
	3		0.872	0.86	0.85	0.84	0.84	0.83
	3.5		0.85	0.84	0.84	0.83	0.82	0.82
	4		0.80	0.79	0.78	0.78	0.77	0.77
	4.5	0.77	0.75					
	5							
1.25	1							
	1.5							
	2							
	2.5		0.86	0.83	0.82	0.81	0.80	0.80
	3		0.872	0.86	0.85	0.84	0.84	0.83
	3.5		0.86	0.84	0.83	0.83	0.83	0.82
	4		0.80	0.79	0.78	0.78	0.77	0.77
	4.5		0.75					
	5							
1.5	1							
	1.5							
	2	0.79						
	2.5		0.86	0.84	0.82	0.81	0.80	0.79
	3		**0.874**	0.86	0.85	0.84	0.84	0.83
	3.5		0.86	0.84	0.84	0.83	0.83	0.82
	4			0.79	0.78	0.78	0.78	0.77
	4.5			0.75				
	5							
1.75	1							
	1.5							
	2	0.80	0.75					
	2.5		0.86	0.84	0.82	0.81	0.80	0.79
	3			0.86	0.85	0.84	0.84	0.84
	3.5			0.84	0.84	0.83	0.83	0.83
	4				0.79	0.78	0.78	0.77
	4.5							
	5							
2	1							
	1.5							
	2		0.75					
	2.5			0.84	0.82	0.81	0.80	0.79
	3				0.85	0.84	0.84	0.84
	3.5					0.83	0.83	0.83
	4					0.78	0.78	0.77
	4.5							
	5							
2.25	1							
	1.5							
	2							
	2.5							
	3							
	3.5							
	4							
	4.5							
	5							
2.5	1							
	1.5							
	2							
	2.5							
	3							
	3.5							
	4							
	4.5							
	5							

Figure S6. Optimisation index for baseline D0 and D14 hPSC-CMs with a target APD_{90} of 330 ms, computed for full block of the funny current (I_f) and different combinations of upscaling the inward rectifier K^+ current conductance (G_{K1}), the maximum Na^+-K^+ pump current (P_{NaK}), and the rapid delayed outward rectifier K^+ current conductance (G_{Kr}). Dark green indicates a high scoring. Scores below 0.75 are not numbered. Note, no score was computed if the cells were still beating spontaneously or could not be paced. Highlighted is the best performing scaling combination.

	PNaK	1.5	1.75	2	2.25	2.5	2.75	3
GK1	GKr							
	1							
	1.5							
	2							
	2.5							
1	3							
	3.5							
	4							
	4.5	0.87	0.84	0.82	0.81	0.80	0.79	0.79
	5	0.95	0.9270	0.92	0.91	0.90	0.89	0.89
	1							
	1.5							
	2							
	2.5							
1.25	3							
	3.5							
	4							
	4.5		0.83	0.81	0.80	0.79	0.78	0.77
	5		0.9266	0.91	0.90	0.89	0.88	0.87
	1							
	1.5							
	2							
	2.5							
1.5	3							
	3.5							
	4							
	4.5			0.81	0.79	0.78	0.77	0.76
	5			0.90	0.89	0.88	0.87	0.87
	1							
	1.5							
	2							
	2.5							
1.75	3							
	3.5							
	4							
	4.5				0.79	0.78	0.77	0.76
	5				0.89	0.88	0.87	0.86
	1							
	1.5							
	2							
	2.5							
2	3							
	3.5							
	4							
	4.5						0.77	0.76
	5							0.86
	1							
	1.5							
	2							
	2.5							
2.25	3							
	3.5							
	4							
	4.5							
	5							
	1							
	1.5							
	2							
	2.5							
2.5	3							
	3.5							
	4							
	4.5							
	5							

Figure S7. Optimisation index for baseline D0 and D14 hPSC-CMs with a target APD_{90} of 170 ms, computed for full block of the funny current (I_f) and different combinations of upscaling the inward rectifier K^+ current conductance (G_{K1}), the maximum Na^+-K^+ pump current (p_{NaK}), and the rapid delayed outward rectifier K^+ current conductance (G_{Kr}). Dark green indicates a high score. Scores below 0.75 are not numbered. Note, no score was computed if the cells were still beating spontaneously or could not be paced. Highlighted are the top three performing scalings with the selected one in yellow.

S3. Human electrophysiological modelling and simulation of chronic MI

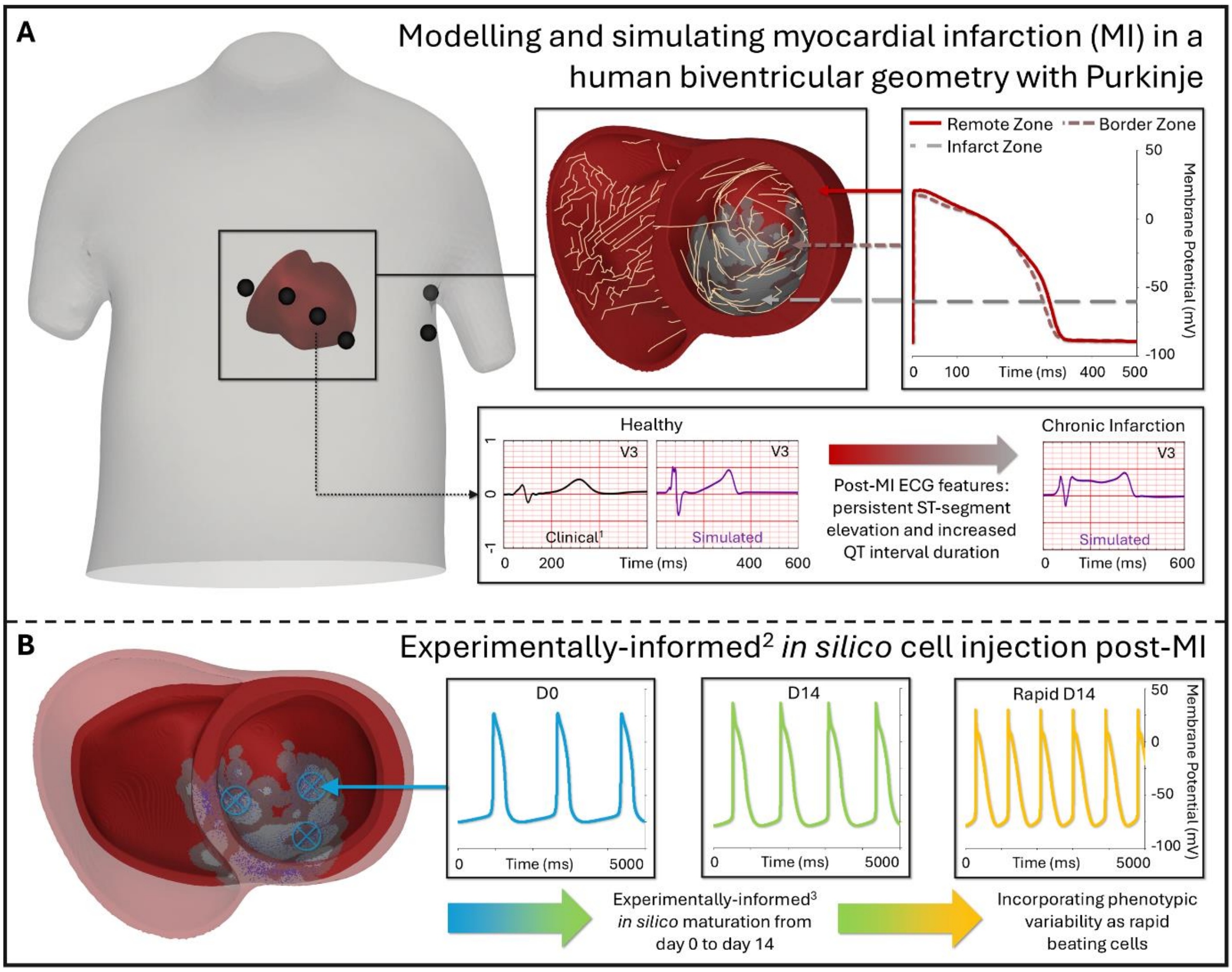

Figure S8. Multiscale modelling and simulation of human ventricular electrophysiology in A) chronic MI and B) cell therapy post-MI using a D0, D14, and rapid beating hPSC-CM model. [1]Biventricular geometry and ECG recordings from Mincholé et al. (2019). [2]Spatial distribution of hPSC-CMs was informed by Poch et al. (2022). [3]Maturation from D0 to D14 was simulated based on in vivo gene expression data from Marchiano et al. (2023).

Table S1 below summarises ionic scaling factors that were applied to model epicardial (based on the baseline endocardial) human ventricular cardiomyocytes and an apicobasal gradient.

Table S1. Ionic current scaling factors to produce I) epicardial human ventricular cardiomyocytes from the baseline endocardial phenotype as in [1]Tomek et al. (2019, 2020) resulting in a transmural action potential duration gradient of 35 ms, and II) a ventricular apex to base gradient as established by [2]Okada et al. (2011) and Mincholé et al. (2019) resulting in an apicobasal repolarisation gradient of 10 ms. V_m denotes the membrane voltage.

**Model parameter**	**Epicardial cell type[1]**	**Apex to base gradient[2]**
G_{NaL}	*0.6	
P_{NaK}	*0.9	
G_{to}	*2.0	
$delta_{epi}$	$= 1 - \frac{0.95}{1 + e^{\frac{V_m + 70}{5}}}$	
G_{Kr}	*1.3	
G_{Ks}	*1.4	*gradient between 0.2 at the base and 5.0 at the apex
G_{K1}	*1.2	

G_{Kb}	*0.6	
G_{NaCa}	*1.1	
P_{Ca}	*1.2	
J_{upnp}	*1.3	
J_{upp}	*1.3	
$cmdn_{max}$	*1.3	

Table S2 below outlines remodelling of cellular ion channel conductances and time constants that was applied to simulate chronically infarcted human cardiomyocytes in the infarct, border, and remote zone.

Table S2. Border and remote zone remodelling as established by Zhou et al. (2024). Infarct zone modelling as suggested by [7]Ringenberg et al. (2014). Supporting experimental data from [1]Valdivia et al. (2005), [2]Hegyi et al. (2018), [3]Jiang et al. (2002), [4]Høydal et al. (2018), [5]Maier et al. (2003), and [6]Hoch et al. (1999).

**Model Parameter**	**Remote Zone**	**Border Zone**	**Infarct Zone**
V_m (set not scaled)			-60 mV[7]
G_{Na}	0.43[1]	0.43[1]	
G_{NaL}	1.413[2]	1.275[2]	
G_{Kr}	0.87[2]	0.89[2]	
G_{K1}		0.76[2]	
G_{KCa}	2[2]	2[2]	
P_{Ca}		0.7[2]	
J_{up}	0.4[3,4]	0.4[3,4]	
tau_{relp}	6[5]	6[5]	
aCaMK	1.5[6]	1.5[6]	
G_{ClCa}	1.25[2]	1.25[2]	

S4. Modelling and simulation of cell therapy

Table S3 below shows conduction velocities that were achieved in the adult myocardium as well as the virtually injected hPSC-CMs.

Table S3. Conduction velocities of adult ventricular tissue and hPSC-CMs. Using conductivity tensors of (2.60, 1.05, 1.60) mS/cm in the remote zone, (0.60, 0.35, 0.45) mS/cm in the border and infarct zone, and (0.2, 0.2, 0.2) mS/cm in hPSC-CMs. Measured in a 20x7x3 cm tissue slab stimulated in the bottom corner, as suggested in benchmark tests by Niederer et al. (2011). Note, hPSC-CM connectivity was modelled as isotropic.

**Cell type**		**Conduction Velocity (cm/s)**		
		Fibre	Sheet	Normal
Adult ventricular myocardium	Remote Zone	49	27	34
	Border Zone	19	12	15
	Infarct Zone	9	7	8
Stem cell-derived cardiomyocytes	D0 (baseline Paci2020 model)	10		
	Rapid D0	9		
	D0 with ×0 I_f ↑50% G_{K1} ↑75% p_{NaK} ↑200% G_{Kr}	12.5		

D0 with ×0 I_f ↑25% G_{K1} ↑75% p_{NaK} ↑400% G_{Kr}	12.5
D14	14.5
Rapid D14	12.5
D14 with ×0 I_f ↑25% G_{K1} ↑75% p_{NaK} ↑400% G_{Kr}	18

Simulated ECGs from before and after virtual cell injection are shown in Figure S9 below. Delivery of hPSC-CMs at D0 caused ST-elevation in leads V1 to V3 as well as T-wave depression in leads V4 and V5. Virtual injection of hPSC-CMs at D14 increased QRS fragmentation and J-point elevation in leads V1 to V3 as well as depressed T-waves in leads V4 and V5. Furthermore, as conduction velocity increased in hPSC-CMs from D0 to D14 (see Table S3), activation of the scar improved, which is visible in the ECG as reduced QRS-prolongation. Virtual delivery of optimised hPSC-CMs at D0 and D14 further depressed the T-wave, also in leads V2 and V3.

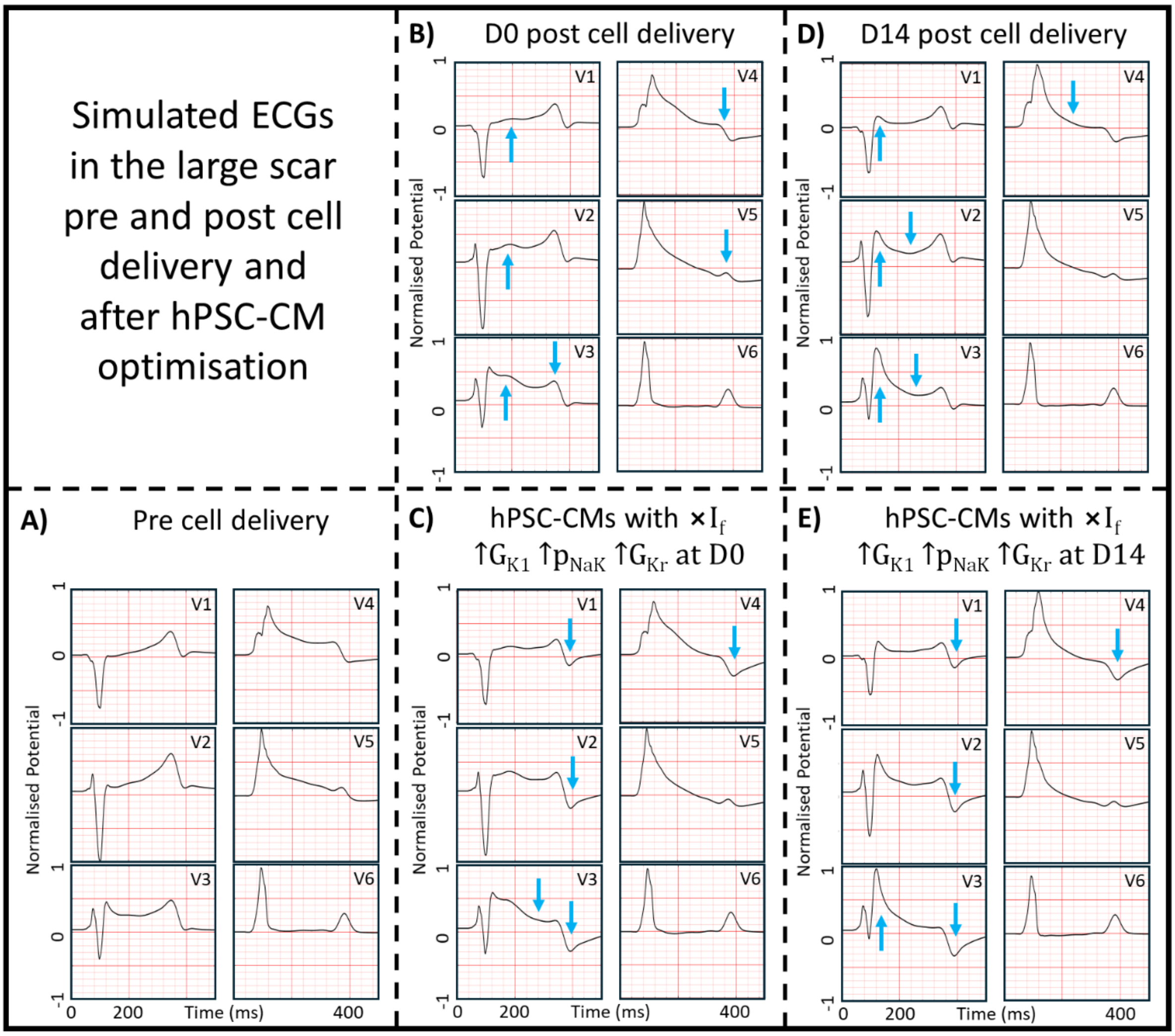

Figure S9. Simulated ECG before and at D0 and D14 after virtual delivery of stem cell-derived cardiomyocytes (hPSC-CMs) before and after optimisation (i.e., I_f block and G_{K1}, p_{NaK}, and G_{Kr} upregulation). Blue arrows indicate prominent changes from panels A) to B), B) to C), B) to D), and D) to E).

Table S4 below details input parameters for the Monodomain solver MonoAlg3D (Berg et al., 2026; Sachetto Oliveira et al., 2018).

Table S4. Parameter files to configure MonoAlg3D (Berg et al., 2026; Sachetto Oliveira et al., 2018) for 3D simulations.

MonoAlg3D Parameter	ToR-ORd	Paci2020	Trovato
Space adaptivity	false		
PDE timestep	0.01		
PDE solved in the…	GPU		
ODE time step	0.01		
ODE solver method	Rush-Larsen + Euler		
ODE adaptivity	false		
ODE solved in the…	GPU		CPU
Linear system solver gradient function	Biconjugate		Conjugate
Linear system solver tolerance	1e-12		1e-16

Table S5 and Table S6 below introduce scaling factors for ionic current conductances to simulate baseline and rapid hPSC-CM phenotypes at D0 and D14 and further show their resulting action potential biomarkers.

Table S5. Ion channel conductance scaling factors to produce D14 (based on experimental gene expression data from Marchiano et al. (2023, Figure 1F)) and rapid beating hPSC-CMs using Paci2020 (Paci et al., 2020) as the baseline D0 model.

Model Parameter	D14	Rapid D0	Rapid D14
G_f	1.25	2.50	3.125
G_{K1}	1.25		1.250
G_{to}	1.50		1.500
G_{Ks}	2.00		2.000
G_{Kr}	1.75		1.750
G_{NaF}	2.50		2.500
k_{NaCa}	2.00	2.50	5.000
G_{CaL}	3.00		3.000
$V_{max,up}$		2.50	2.500

Table S6. AP biomarkers for D0, D14, and rapid hPSC-CM phenotypes after 1,000 seconds of spontaneous activity using Paci2020 (Paci et al., 2020). DDR: diastolic depolarisation rate, MDP: maximum diastolic potential, dV/dt_{max}: maximum upstroke velocity, $V_{m,peak}$: peak V_m, APD_{90}: APD at 90% repolarisation, F: spontaneous beating frequency..

hPSC-CM phenotype	DDR (V/s)	MDP (mV)	dV/dt_{max} (V/s)	$V_{m,peak}$ (mV)	APD_{90} (mV)	F (bpm)
D0	0.01	-74.87	20.54	27.14	402.42	35
Rapid D0	0.02	-74.90	26.49	18.76	424.27	52
D14	0.02	-78.79	172.26	36.53	446.31	47
Rapid D14	0.03	-78.57	168.01	29.94	414.57	66

S5. Verification, validation, and uncertainty quantification

To quantify uncertainty, we carried out further sensitivity analyses. First, as mentioned in the stimulation protocols in the main text, biventricular simulations used the unpaced hPSC-CM models after 1,000 s as a starting state. We chose unpaced rather than paced activity, as coupling between hPSC-CMs and surrounding tissue, particularly within the scar, may be incomplete impacting their pacing. However, in isolation, the optimised hPSC-CMs' spontaneous beating was suppressed and only a few APs occurred at the start of the unpaced 1,000 s. This leads to uncertainty regarding the models' action potential after possible pacing in the ventricles, especially as we changed I_{NaK}, which may substantially affect ionic concentrations. Hence, we compared the action potentials of optimised hPSC-CMs (full I_f block and 25%, 75%, and 400% I_{K1} I_{NaK}, and I_{Kr} upregulation, respectively) after 1,000 s of unpaced activity followed by 3 paced beats (mimicking the biventricular simulation setting) versus 1,000 paced beats. As shown in Figure S10 A) below, this produced small changes in the D0 model (APD_{90} difference of approximately 10 ms). At D14 (panel B), the calcium transient amplitude increased about 3-fold over time and the action potential shape changed more substantially (reduced peak and steeper repolarisation). However, APD_{90} changed only from 230 to 245 ms with increased pacing duration.

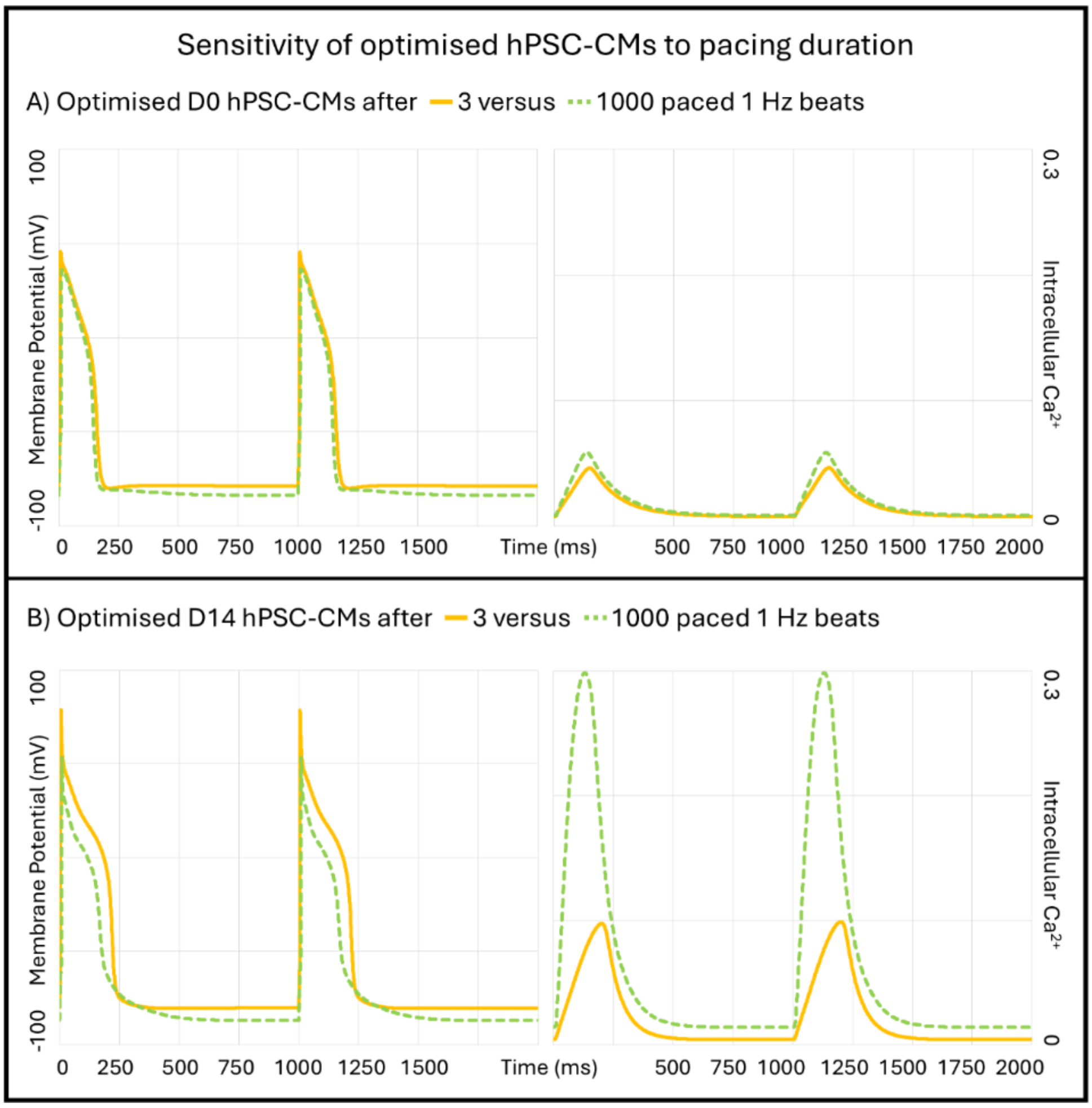

Figure S10. Sensitivity of optimised stem cell-derived cardiomyocytes (hPSC-CMs) to pacing duration. Isolated hPSC-CMs at A) D0 and B) D14 with full block of the funny current, 25% upregulation of the inward rectifier K^+ current's conductance, 75%

upregulation of maximum Na^+-K^+ pump current, and 400% upregulation of the rapid delayed outward rectifier K^+ current's conductance. Simulated in single cell for 1,000 s of unpaced activity followed by 3 s (solid yellow traces) or 1,000 s (dashed green traces) of 1 Hz pacing.

Next, we investigated the sensitivity to differences in ionic concentrations, as these differ slightly in the hPSC-CM Paci2020 model and the adult ventricular cardiomyocyte ToR-ORd model. Specifically, extracellular K^+ and Na^+ concentrations are 151 versus 140 and 5.4 versus 5.0 mM in the Paci2020 versus ToR-ORd model, respectively. Considering that ionic concentrations are variable spatially and over time, in our simulations, we did not change these, also allowing for comparability to previous studies. Comparison on the effect of changing the Paci2020 concentrations to the ToR-ORd values, shown in Figure S11, showed small changes in the spontaneous beating frequency (35 bpm before versus 36 bpm after changing concentrations) and the paced APD_{90} (455 before versus 440 ms after changing concentrations).

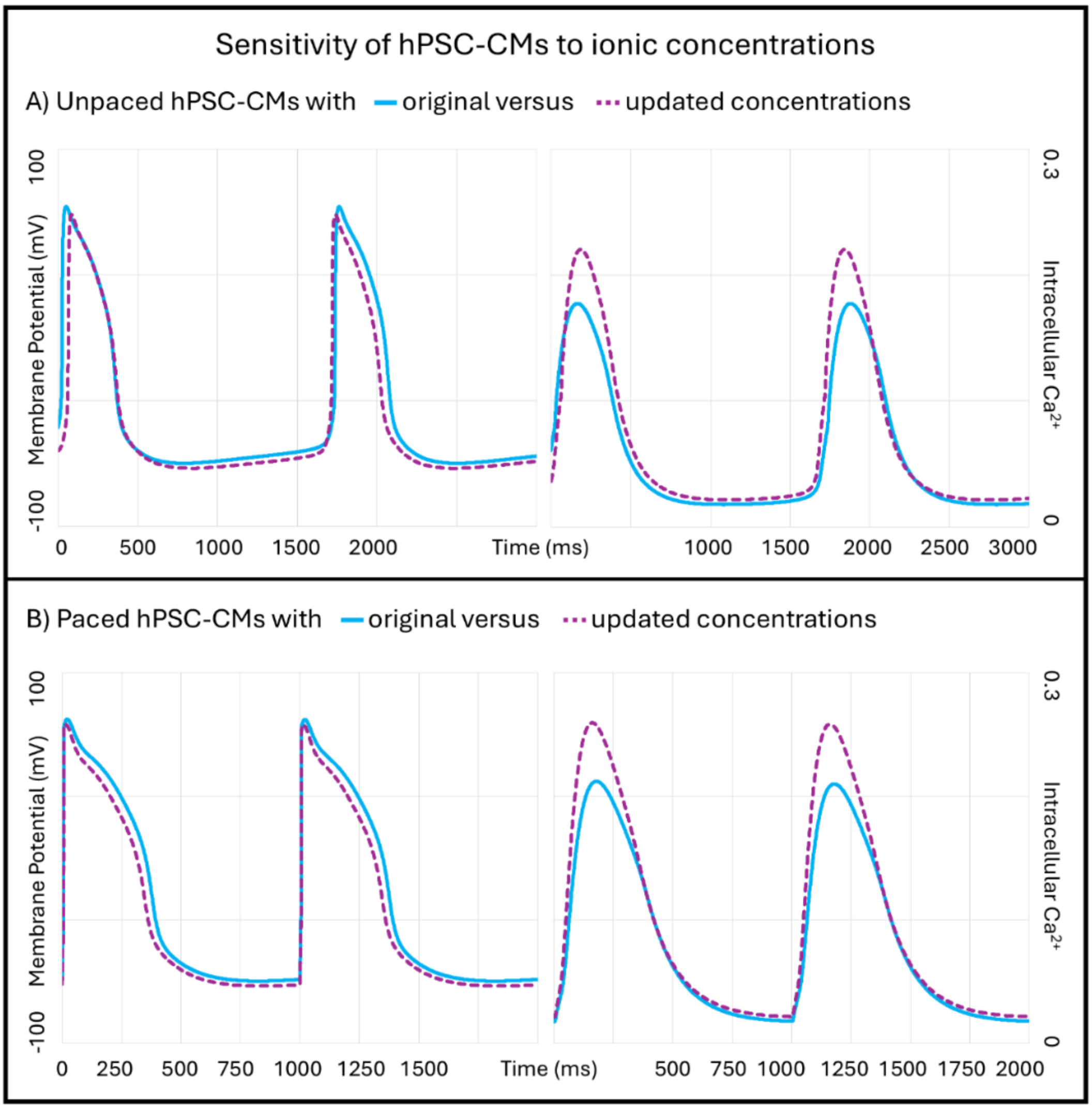

Figure S11. Sensitivity of D0 hPSC-CMs in isolation to ionic concentrations. Original hPSC-CM Paci2020 model (Paci et al., 2020) with extracellular Na^+ of 151 mM and extracellular K^+ of 5.4 mM (solid blue traces) or using ionic concentrations from the adult ventricular ToR-ORd model (Tomek et al., 2019, 2020), i.e., extracellular Na^+ of 140 mM and extracellular K^+ of 5 mM (dashed purple traces). A) 1,000 s of spontaneous beating were simulated and B) followed by 3 s of 1 Hz pacing.

Supplemental References

Berg, L. A., Oliveira, R. S., Camps, J., de Lima, L. M. R., de Oliveira Campos, J., Wang, Z. J., Doste, R., Bueno-Orovio, A., dos Santos, R. W., & Rodriguez, B. (2026). Toward cardiac electrophysiology digital twins with an efficient open source scalable solver on GPU clusters. *Scientific Reports*, *16*(1), 9619. https://doi.org/10.1038/s41598-025-33709-w

Hegyi, B., Bossuyt, J., Griffiths, L. G., Shimkunas, R., Coulibaly, Z., Jian, Z., Grimsrud, K. N., Sondergaard, C. S., Ginsburg, K. S., Chiamvimonvat, N., Belardinelli, L., Varró, A., Papp, J. G., Pollesello, P., Levijoki, J., Izu, L. T., Boyd, W. D., Bányász, T., Bers, D. M., & Chen-Izu, Y. (2018). Complex electrophysiological remodeling in postinfarction ischemic heart failure. *Proceedings of the National Academy of Sciences*, *115*(13), E3036–E3044. https://doi.org/10.1073/pnas.1718211115

Hoch, B., Meyer, R., Hetzer, R., Krause, E.-G., & Karczewski, P. (1999). Identification and Expression of δ-Isoforms of the Multifunctional Ca^{2+}/Calmodulin-Dependent Protein Kinase in Failing and Nonfailing Human Myocardium. *Circulation Research*, *84*(6), 713–721. https://doi.org/10.1161/01.RES.84.6.713

Høydal, M. A., Kirkeby-Garstad, I., Karevold, A., Wiseth, R., Haaverstad, R., Wahba, A., Stølen, T. L., Contu, R., Condorelli, G., Ellingsen, Ø., Smith, G. L., Kemi, O. J., & Wisløff, U. (2018). Human cardiomyocyte calcium handling and transverse tubules in mid-stage of post-myocardial-infarction heart failure. *ESC Heart Failure*, *5*(3), 332–342. https://doi.org/10.1002/ehf2.12271

Jiang, M. T., Lokuta, A. J., Farrell, E. F., Wolff, M. R., Haworth, R. A., & Valdivia, H. H. (2002). Abnormal Ca^{2+} Release, but Normal Ryanodine Receptors, in Canine and Human Heart Failure. *Circulation Research*, *91*(11), 1015–1022. https://doi.org/10.1161/01.RES.0000043663.08689.05

Maier, L. S., Zhang, T., Chen, L., DeSantiago, J., Brown, J. H., & Bers, D. M. (2003). Transgenic CaMKIIδ_C Overexpression Uniquely Alters Cardiac Myocyte Ca^{2+} Handling. *Circulation Research*, *92*(8), 904–911. https://doi.org/10.1161/01.RES.0000069685.20258.F1

Marchiano, S., Nakamura, K., Reinecke, H., Neidig, L., Lai, M., Kadota, S., Perbellini, F., Yang, X., Klaiman, J. M., Blakely, L. P., Karbassi, E., Fields, P. A., Fenix, A. M., Beussman, K. M., Jayabalu, A., Kalucki, F. A., Potter, J. C., Futakuchi-Tsuchida, A., Weber, G. J., … Murry, C. E. (2023). Gene editing to prevent ventricular arrhythmias associated with cardiomyocyte cell therapy. *Cell Stem Cell*, *30*(4), 396-414.e9. https://doi.org/10.1016/j.stem.2023.03.010

Mincholé, A., Zacur, E., Ariga, R., Grau, V., & Rodriguez, B. (2019). MRI-Based Computational Torso/Biventricular Multiscale Models to Investigate the Impact of Anatomical Variability on the ECG QRS Complex. *Frontiers in Physiology*, *10*. https://doi.org/10.3389/fphys.2019.01103

Niederer, S. A., Kerfoot, E., Benson, A. P., Bernabeu, M. O., Bernus, O., Bradley, C., Cherry, E. M., Clayton, R., Fenton, F. H., Garny, A., Heidenreich, E., Land, S., Maleckar, M., Pathmanathan, P., Plank, G., Rodríguez, J. F., Roy, I., Sachse, F. B., Seemann, G., … Smith, N. P. (2011). Verification of cardiac tissue electrophysiology simulators using an N-version benchmark. *Philosophical Transactions of the Royal Society A: Mathematical, Physical and Engineering Sciences*, *369*, 4331–4351. https://doi.org/10.1098/rsta.2011.0139

Okada, J., Washio, T., Maehara, A., Momomura, S., Sugiura, S., & Hisada, T. (2011). Transmural and apicobasal gradients in repolarization contribute to T-wave genesis in human surface ECG. *American Journal of Physiology-Heart and Circulatory Physiology*, *301*(1), H200–H208. https://doi.org/10.1152/ajpheart.01241.2010

Paci, M., Passini, E., Klimas, A., Severi, S., Hyttinen, J., Rodriguez, B., & Entcheva, E. (2020). All-Optical Electrophysiology Refines Populations of In Silico Human iPSC-CMs for Drug Evaluation. *Biophysical Journal*, *118*(10), 2596–2611. https://doi.org/10.1016/j.bpj.2020.03.018

Poch, C. M., Foo, K. S., De Angelis, M. T., Jennbacken, K., Santamaria, G., Bähr, A., Wang, Q.-D., Reiter, F., Hornaschewitz, N., Zawada, D., Bozoglu, T., My, I., Meier, A., Dorn, T., Hege, S., Lehtinen, M. L., Tsoi, Y. L., Hovdal, D., Hyllner, J., … Laugwitz, K.-L. (2022). Migratory and anti-fibrotic programmes define the regenerative potential of human cardiac progenitors. *Nature Cell Biology*, *24*, 659–671. https://doi.org/10.1038/s41556-022-00899-8

Ringenberg, J., Deo, M., Filgueiras-Rama, D., Pizarro, G., Ibañez, B., Peinado, R., Merino, J. L., Berenfeld, O., & Devabhaktuni, V. (2014). Effects of Fibrosis Morphology on Reentrant Ventricular Tachycardia Inducibility and Simulation Fidelity in Patient-Derived Models. *Clinical Medicine Insights: Cardiology*, *8s1*. https://doi.org/10.4137/CMC.S15712

Romero, L., Pueyo, E., Fink, M., & Rodríguez, B. (2009). Impact of ionic current variability on human ventricular cellular electrophysiology. *American Journal of Physiology-Heart and Circulatory Physiology*, *297*(4), H1436–H1445. https://doi.org/10.1152/ajpheart.00263.2009

Sachetto Oliveira, R., Martins Rocha, B., Burgarelli, D., Meira, W., Constantinides, C., & Weber dos Santos, R. (2018). Performance evaluation of GPU parallelization, space-time adaptive algorithms, and their combination for simulating cardiac electrophysiology. *International Journal for Numerical Methods in Biomedical Engineering*, *34*(2), e2913. https://doi.org/10.1002/cnm.2913

Tomek, J., Bueno-Orovio, A., Passini, E., Zhou, X., Minchole, A., Britton, O., Bartolucci, C., Severi, S., Shrier, A., Virag, L., Varro, A., & Rodriguez, B. (2019). Development, calibration, and validation of a novel human ventricular myocyte model in health, disease, and drug block. *eLife*, *8*, e48890. https://doi.org/10.7554/eLife.48890

Tomek, J., Bueno-Orovio, A., & Rodriguez, B. (2020). ToR-ORd-dynCl: an update of the ToR-ORd model of human ventricular cardiomyocyte with dynamic intracellular chloride. *BioRxiv [Preprint]*. https://doi.org/10.1101/2020.06.01.127043

Valdivia, C. R., Chu, W. W., Pu, J., Foell, J. D., Haworth, R. A., Wolff, M. R., Kamp, T. J., & Makielski, J. C. (2005). Increased late sodium current in myocytes from a canine heart failure model and from failing human heart. *Journal of Molecular and Cellular Cardiology*, *38*(3), 475–483. https://doi.org/10.1016/j.yjmcc.2004.12.012

Zhou, X., Wang, Z. J., Camps, J., Tomek, J., Santiago, A., Quintanas, A., Vazquez, M., Vaseghi, M., & Rodriguez, B. (2024). Clinical phenotypes in acute and chronic infarction explained through human ventricular electromechanical modelling and simulations. *eLife*, *13*, RP93002. https://doi.org/https://doi.org/10.7554/eLife.93002.3
